# Integration of imaging-based and sequencing-based spatial omics mapping on the same tissue section via DBiTplus

**DOI:** 10.1038/s41592-025-02948-0

**Published:** 2026-01-15

**Authors:** Archibald Enninful, Zhaojun Zhang, Dmytro Klymyshyn, Matthew Ingalls, Mingyu Yang, Hailing Zong, Zhiliang Bai, Negin Farzad, Graham Su, Alev Baysoy, Jungmin Nam, Yao Lu, Shuozhen Bao, Siyan Deng, Nancy R. Zhang, Oliver Braubach, Mina L. Xu, Zongming Ma, Rong Fan

**Affiliations:** 1 Department of Biomedical Engineering, Yale University, New Haven, CT USA; 2 Department of Statistics and Data Science, The Wharton School, University of Pennsylvania, Philadelphia, PA USA; 3 Akoya Biosciences, Menlo Park, CA USA; 4 Bruker Spatial Biology, St Louis, MO USA; 5 Department of Pathology, Yale School of Medicine, New Haven, CT USA; 6 Department of Statistics and Data Science, Yale University, New Haven, CT USA; 7 Yale Stem Cell Center and Yale Cancer Center, Yale School of Medicine, New Haven, CT USA; 8 Human and Translational Immunology Program, Yale School of Medicine, New Haven, CT USA; 9 Yale Center for Research on Aging (Y-Age), Yale School of Medicine, New Haven, CT USA

**Keywords:** Fluorescence imaging, RNA sequencing, Data integration

## Abstract

Spatially mapping the transcriptome and proteome in the same tissue section can profoundly advance our understanding of cellular heterogeneity and function. Here we present Deterministic Barcoding in Tissue sequencing plus (DBiTplus), an integrative multimodal spatial omics approach combining sequencing-based spatial transcriptomics and multiplexed protein imaging on the same section, enabling both single-cell-resolution cell typing and transcriptome-wide interrogation of biological pathways. DBiTplus utilizes spatial barcoding and RNase H-mediated cDNA retrieval, preserving tissue architecture for multiplexed protein imaging. We developed computational pipelines to integrate these modalities, allowing imaging-guided deconvolution to generate single-cell-resolved spatial transcriptome atlases. We demonstrate DBiTplus across diverse samples including frozen mouse embryos, and formalin-fixed paraffin-embedded human lymph nodes and lymphoma tissues, highlighting its compatibility with challenging clinical specimens. DBiTplus uncovered mechanisms of lymphomagenesis, progression and transformation in human lymphomas. Thus, DBiTplus is a unified workflow for spatially resolved single-cell atlasing and unbiased exploration of biological mechanisms in a cell-by-cell manner at transcriptome scale.

## Abstract (web summary)

DBiTplus represents an integrated experimental and computational workflow that unifies sequencing-based and imaging-based spatial omics, demonstrated by combining transcriptomic profiling and multiplexed protein imaging on the same tissue section.

## Main

The advent of high-throughput single-cell technologies has revolutionized our understanding of biological systems, enabling comprehensive analyses at the molecular level across diverse biological contexts^1^. These approaches lack spatial context, limiting our understanding of intercellular interactions within tissues. Spatial omics addresses this limitation using a wide range of approaches. Array-based approaches such as Slide-seq^2^ utilize spatially barcoded surfaces to capture mRNA transcripts. Tissue barcoding approaches such as DBiT-seq^3^, spatial CITE-seq^4^ and spatial ATAC-seq^5^ use spatially defined delivery of DNA barcodes to profile the transcriptome, proteome or epigenome. Imaging-based methods such as MERFISH^6^ utilize combinatorial labeling and sequential imaging to achieve subcellular resolution. Similarly, technologies such as STARmap^7^ sequence nucleic acids directly within tissues or cells. Broadly, in situ sequencing or imaging techniques offer higher spatial resolution and sensitivity but may lack transcriptome-wide coverage, whereas sequencing-based methods provide transcriptome-wide coverage, often at the expense of high spatial resolution. Multiplexed protein imaging technologies such as CODEX^8^ have also been developed, offering single-cell resolution profiling of protein markers for in situ cell typing.

Currently, most spatial multi-omic technologies involve running single spatial omics assays separately on adjacent or serial tissue sections, followed by computational data integration of the multimodal datasets. Due to heterogeneity in cellular composition and tissue architecture, even between adjacent sections from the same block, integrating multimodal data computationally may be suboptimal, as perfect concordance between tissue sections is almost unattainable. Thus, new methods for spatially resolved multimodal and multi-omics measurements on the same tissue sections are necessary. Single-cell spatial multimodal metabolomics approaches such as REDCAT combine protein and metabolite measurements in the same tissue section^9^. IN-DEPTH was also recently developed for same-slide spatial multi-omics integration^10^.

Here, we developed DBiTplus, which combines spatially resolved transcriptomics sequencing with multiplexed immunofluorescence (mxIF) imaging for the unbiased co-profiling of the whole-transcriptome and protein markers on the same tissue section. We tested several cDNA retrieval methods, settling on an enzymatic approach using RNase H after spatial barcoding, which maintains tissue integrity and morphology before mxIF imaging. A computational approach, modified from the MaxFuse algorithm^11^, was developed to integrate the multi-omic spatial transcriptomic and mxIF datasets, and a robust cell-type decomposition (RCTD)-like approach^12^ used for DBiTplus spot cell-type deconvolution and spot splitting to generate pure cell-type sub-spots. We applied DBiTplus to elucidate the process of embryogenesis in optimal cutting temperature (OCT)-frozen and paraffin-embedded C57BL/6 (C57) mouse embryo sections. Applying DBiTplus to healthy human lymph node and lymphoma tissues demonstrated the capability to generate high-quality single-cell-resolved transcriptome and protein data from the same tissue section, addressing the hurdles associated with data integration and registration from adjacent sections.

## Results

### Design and overview of DBiTplus

**Fig. 1:**
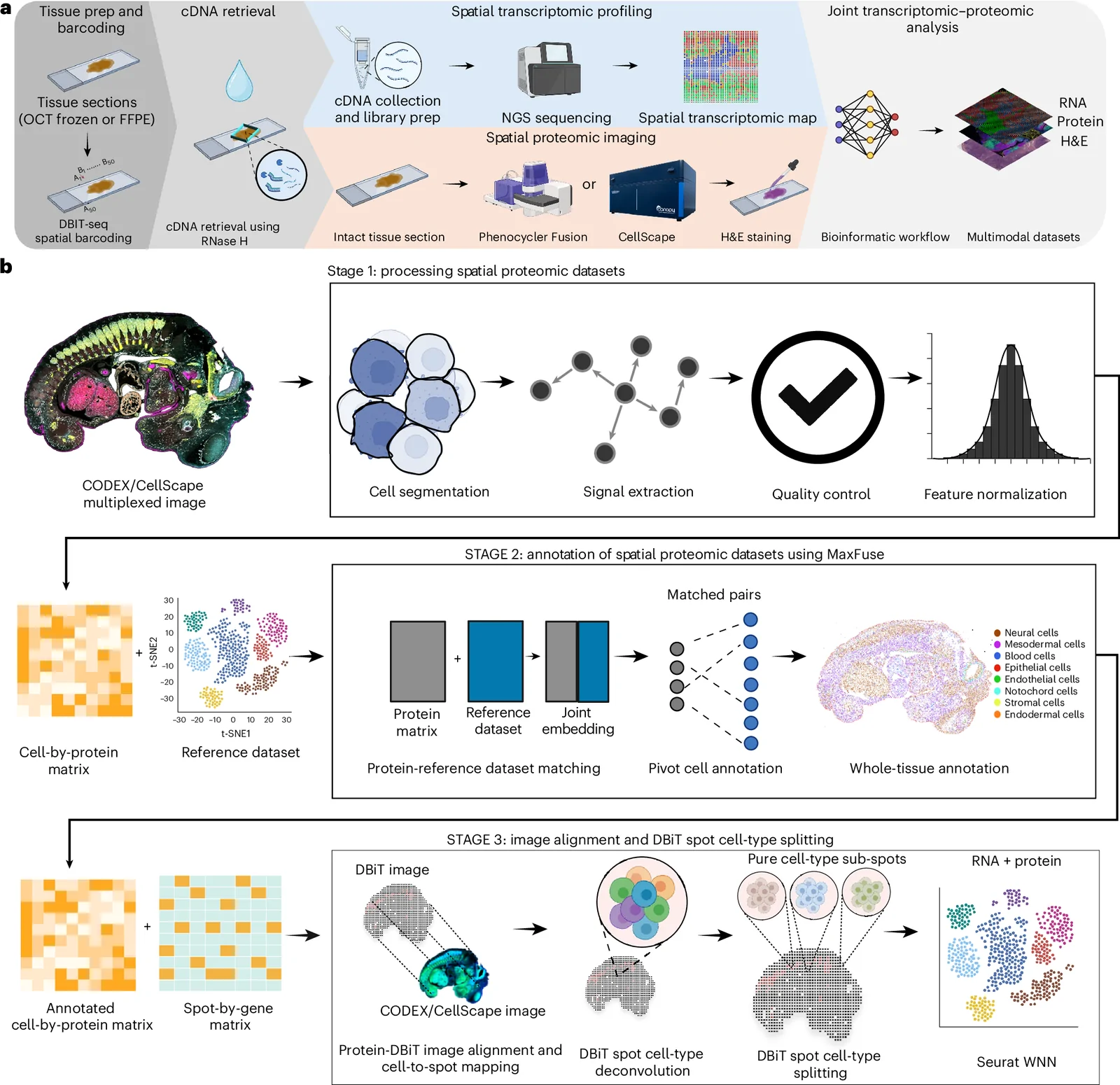
DBiTplus workflow and overview of bioinformatic workflow for integrative multimodal data analysis. **a**, Workflow of DBiTplus technology. **b**, The three steps of the integrative analysis. DBIT-seq, Deterministic Barcoding in Tissue sequencing; NGS, next-generation sequencing. Created with BioRender.com.

**Extended Data Fig. 1:**
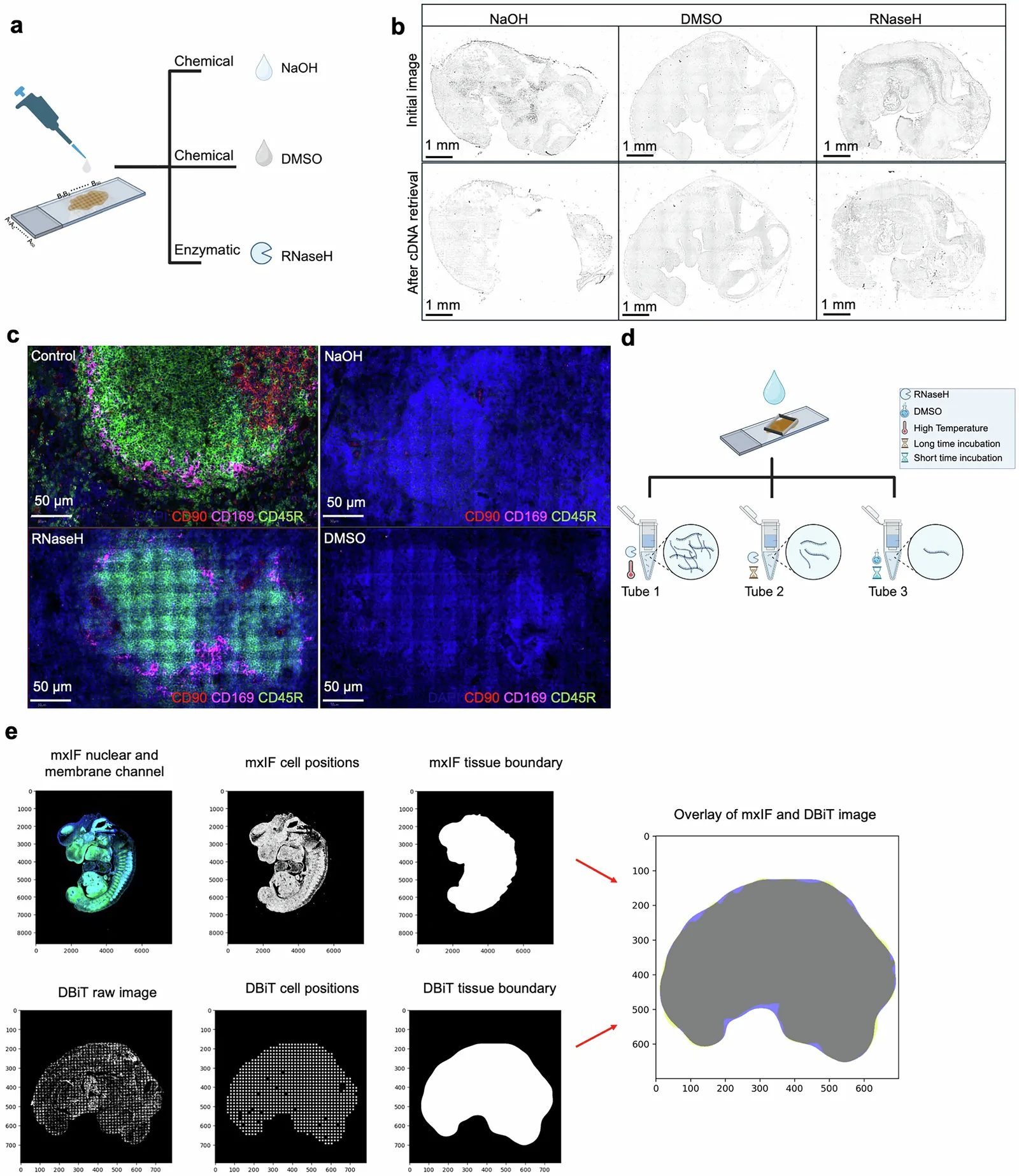
Evaluation of cDNA retrieval strategies for compatibility with DBiTplus and downstream multiplexed immunofluorescence imaging. **a**, Schematic overview of three tested approaches for cDNA release from FF or FFPE tissue sections. Created with BioRender.com. **b**, Brightfield images of E13 mouse embryo FF sections before and after cDNA retrieval using NaOH, DMSO, or RNase H. Morphology is best preserved with the RNase H condition. **c**, Assessing the quality of CODEX imaging on FF mouse spleen after DBiTplus workflow (n = 1). The RNase H-treated sample shows positive staining makers including CD45R (B cell lineage) and CD169 (macrophages) similar to control staining. **d**, Schematic overview for RNase H cDNA retrieval protocol. The three tubes are pooled for cDNA purification, PCR amplification, and library preparation. Created with BioRender.com. **e**, Image registration pipeline between DBiTplus and mxIF datasets.

In the standard DBiT-seq workflow, mRNAs are reverse-transcribed in situ within the tissue matrix, and DNA barcodes A (Ai; i = 1–50) and B (Bj; j = 1–50) delivered perpendicularly through microfluidic chips with 50 parallel channels. The barcodes ligate to form a unique two-dimensional array of barcoded spots. After tissue lysis, barcoded cDNAs are recovered, purified and amplified for paired-end sequencing to generate spatial gene expression maps. This technique has been adapted to profile the transcriptome, epigenome and proteome and, more recently, applied to archival formalin-fixed paraffin-embedded (FFPE) tissue blocks^3–5,13^. Central to the efforts to combine DBiT-seq with mxIF was to develop techniques to release cDNA from tissue sections while maintaining tissue integrity. Two chemical approaches (using sodium hydroxide (NaOH) and dimethylsulfoxide (DMSO)) and an enzymatic approach (using RNase H) were tested (Extended Data Fig. 1a). Following successful retrieval of the cDNA and preparation of a sequencing library, the intact tissue section was imaged using Akoya Biosciences’ PhenoCycler-Fusion platform or Bruker Spatial Biology’s CellScape platform, and routine hematoxylin and eosin (H&E) staining performed on the same tissue section (Fig. 1a). For FFPE samples, the Patho-DBiT workflow^13^ was used (until spatial barcoding was completed), following which the tissue section was incubated at 55 °C with a mix of Triton X-100 and Thermostable RNase H enzyme to break down RNA strands in RNA–DNA hybrids and to facilitate the diffusion through the permeabilized cell membranes. Another overnight incubation at 37 °C was performed to increase cDNA recovery from the tissue. The retrieved cDNAs were pooled and a sequencing library built. The intact tissue section can be stored at −20 °C until mxIF imaging. FFPE tissue sections underwent brief rehydration and antigen retrieval steps before mxIF imaging. After successful imaging, the flow cell could be removed from the slide and routine H&E staining performed on the same section. For fresh frozen samples, the DBiTplus workflow was identical, excluding FFPE-specific steps.

### Development of a computational framework for DBiTplus

We developed a workflow integrating the mxIF cell-by-protein and DBiTplus spot-by-gene matrices into a unified feature matrix with cell-type labels and spatial coordinates (Fig. 1b), starting with whole-cell segmentation of the mxIF data using Mesmer^14^ and quality-control steps. The mxIF datasets were annotated via MaxFuse^11^ integration with a reference single-cell RNA-sequencing (scRNA-seq) dataset such as the Mouse Organogenesis Cell Atlas^15^. Utilizing tissue boundary detection and image transformation, mxIF and DBiT-seq images were co-registered to enable accurate matching and alignment of the same cells across modalities (Extended Data Fig. 1e). Cell-type counts from mxIF segmentation masks were mapped to DBiTplus spots, which were subdivided into pure cell-type sub-spots for cell-type-specific gene expression estimation, enabling single-cell annotation beyond existing deconvolution tools (Fig. 1b).

### Workflow, development and optimization in fresh frozen and FFPE samples

Preliminary experiments on OCT-frozen embryonic day 13 (E13) mouse C57 embryo sections tested cDNA retrieval methods. The barcoded region of the tissue was covered with a clean polydimethylsiloxane (PDMS) well gasket and incubated at room temperature with 50 μl of 0.1 M NaOH for 15 min, which was then collected and neutralized with equimolar hydrochloric acid (HCl). NaOH disrupted tissue morphology (Extended Data Fig. 1b). In mouse spleen serial sections, each section was subjected to a different cDNA retrieval approach: 0.1 M NaOH for 5 min, 90% DMSO for 30 min and 10 U of RNase H at 37 °C for 30 min. The samples were then imaged with a 25-marker mouse immune panel by Akoya Biosciences. DMSO-treated and NaOH-treated sections yielded poor-quality CODEX staining, whereas the RNase H-treated section yielded 14 positively staining markers including CD45R (B cell lineage) and CD169 (macrophages; Extended Data Fig. 1c). Thus, the enzymatic approach was selected for further optimization (Extended Data Fig. 1d).

**Extended Data Fig. 2:**
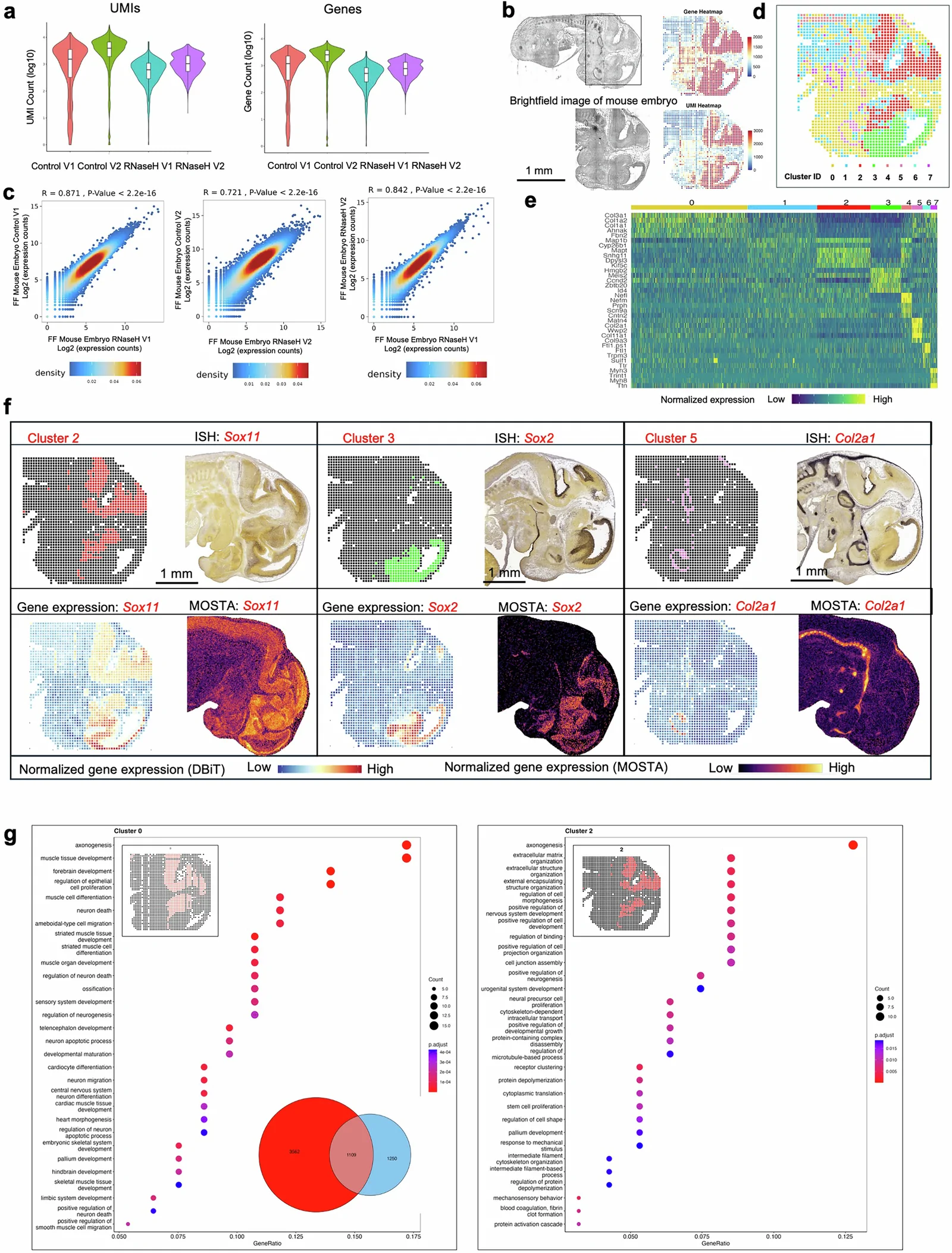
Spatial profiling of gene expression in FF mouse embryo using DBiTplus. **a** Violin plot showing the number of detected genes/UMI counts per spatial spot from from two controls and two replicates FF E13 mouse embryo samples. All samples were downsampled to the same number of reads for comparison (99,000,000 reads). Box plots show the median (center line), the first and third quartiles (box limits) and 1.5x interquartile range (whiskers). **b** Brightfield image of E13 FF mouse embryo. Black box represents the region spatially barcoded during the DBiTplus workflow. Right: spatial mRNA and UMI count maps demonstrates average of 1,029 genes and 1,601 UMIs detected per 25 μm spot. **c** Pairwise correlation plots between replicates (DBiTplus workflow) and control (standard DBiT-seq workflow) showing the reproducibility of the DBiTplus workflow from a two-sided Pearson correlation test, with a Pearson correlation coefficient of 0.87 and 0.72 respectively. Pearson correlation between the two replicates is 0.84. For Replicate 2, 0.5% Triton X-100 was added to the RNase H to further improve cDNA release efficiency. **d** Spatial UMAP reveals 8 transcriptionally distinct clusters that align with anatomical features of the E13 mouse embryo. **e** Heat map of the top 5 differentially expressed genes per spatial cluster. **f** Validation of DBiTplus gene expression using independent reference datasets. In-situ Hybridization (ISH) staining image from the Allen Mouse Brain Atlas (Developing Mouse Brain - Age: E13.5, Theiler Stage: TS21) and gene expression from Mouse Organogenesis Spatiotemporal Transcriptomic Atlas (MOSTA) dataset (E13.5_E1S2.MOSTA.h5ad). Scale bar 1 mm for Allen Mouse Brain Atlas images. **g** Gene Ontology (GO) enrichment analysis showing the biological process associated with E13 mouse embryos (cluster 0 from the standard DBiT-seq workflow and cluster 2 from DBiTplus workflow), both of which are associated with the embryonic diencephalon. Venn diagram shows the overlap of genes between the two clusters.

A new Thermostable RNase H (New England Biolabs, M0523S; optimal activity > 65 °C) was introduced. E13 mouse embryo sections underwent spatial barcoding with proteinase K lysing (control) or RNase H-mediated cDNA release (test). Sequencing detected 24,102 and 20,973 genes, respectively (Extended Data Fig. 2a and Supplementary Table 2). Adding 0.5% Triton X-100 to the RNase H mix did not compromise tissue integrity, but saw an increase in mean gene counts per spot (Extended Data Fig. 2b and Supplementary Table 2). Replicates showed high correlation within and between test and control slides (*R* = 0.87 and *R* = 0.72, respectively), and strong consistency was observed between test replicates (*R* = 0.84; Extended Data Fig. 2c). Spatial clustering identified eight clusters, with clusters 2 and 3 corresponding to the diencephalon, mesencephalon and telencephalon, evidenced by the expression of *Zbtb20* and *Id4* (embryonic neocortex, forebrain; Extended Data Fig. 2d,e). Spatial patterns of select genes—*Sox11*, *Col2a1* and *Sox2*—matched in situ hybridization data from the Allen Brain Atlas and the Mouse Organogenesis Spatiotemporal Transcriptomic Atlas (MOSTA) from STOmicsDB^16^ (Extended Data Fig. 2f). Clusters 0 (control) and 2 (test) showed remarkably similar spatial distributions and 1,109 overlapping genes, underscoring the ability of DBiTplus to generate high-quality spatial transcriptome data and recapitulate tissue biology (Extended Data Fig. 2g).

We assessed the feasibility of the workflow in FFPE tissues, the ‘gold standard’ for preserving clinical tissues. We profiled human cerebellum and lymph node FFPE sections, which retained their architecture after spatial barcoding and cDNA recovery (cDNA size ranged from 200 to 800 base pairs; Supplementary Fig. 1a–c). CODEX imaging resolved distinct cerebellar layers (granular, Purkinje and molecular layers). AQP4^+^ glial cells were observed across all three layers, and the single layer of large pear-shaped CALB1^+^ Purkinje cell bodies was most prominent in the Purkinje cell layer (Supplementary Fig. 1d). Lymph node CODEX staining recapitulated expected architecture, including CD20^+^ B cells and CD21^+^ follicular dendritic cells in the follicle, and CD3ε^+^ T cells in interfollicular regions (Supplementary Fig. 1e).

### Multimodal mapping of mouse embryo

**Fig. 2:**
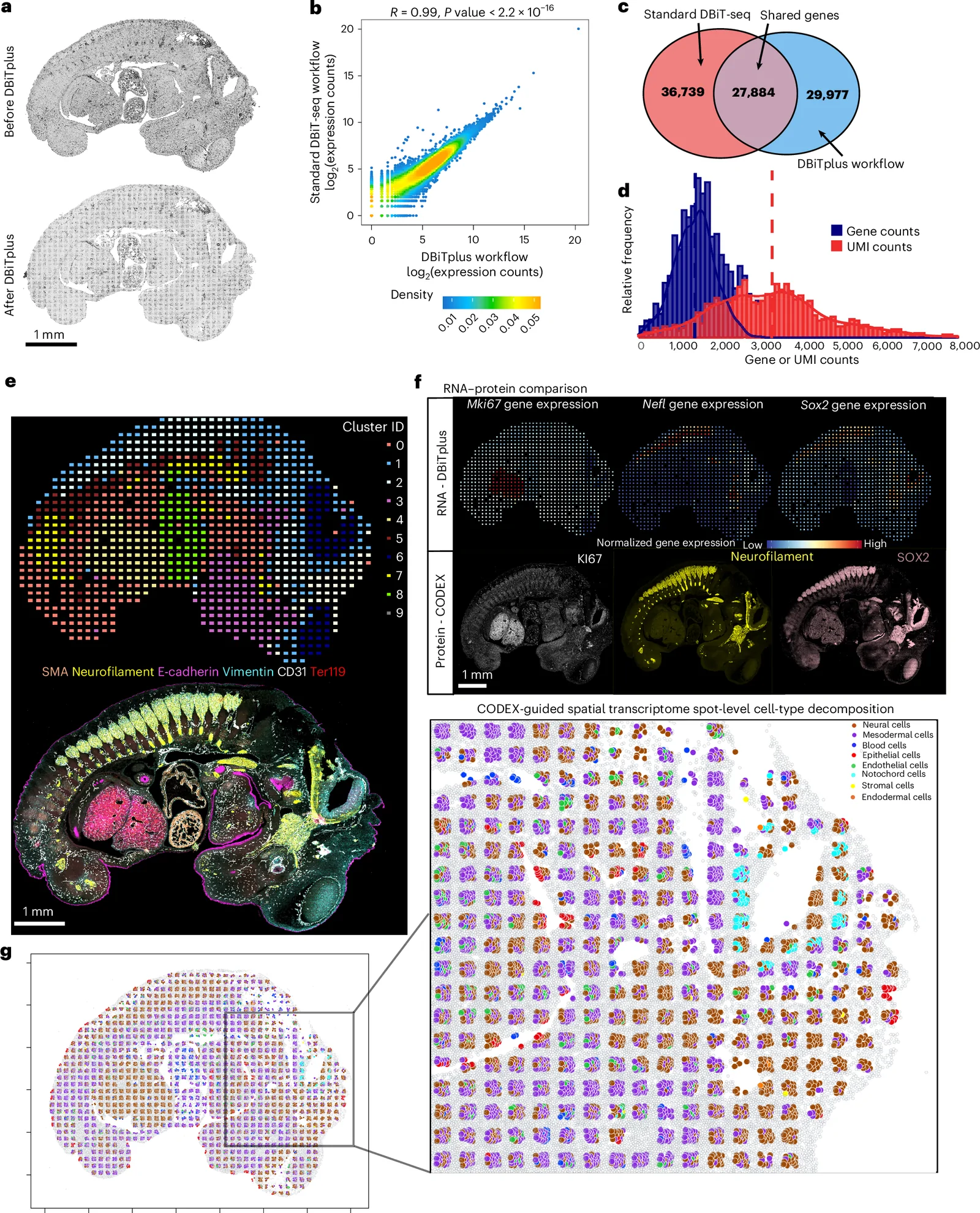
DBiTplus performance and spatial multi-omic analysis of FFPE mouse embryo. **a**, Brightfield images of an E11 FFPE mouse embryo section before (top) and after (bottom) the DBiTplus workflow, showing that tissue integrity and morphology are preserved following cDNA retrieval (*n* = 2). **b**, Correlation analysis between two FFPE E11 mouse embryo section samples comparing the standard DBiT-seq and the DBiTplus workflows from a two-sided Pearson correlation test (Pearson correlation = 0.99 and *P* value < 2.2 × 10^−16^). **c**, Venn diagram showing overlap of genes sequenced between the standard DBiT workflow and the DBiTplus workflow. **d**, Distribution of detected genes and UMIs per spatial spot. Blue and red dashed lines represent the average gene and UMI counts, respectively. **e**, Top, UMAP clustering of spatial transcriptomic data identified ten distinct transcriptomic clusters from the E11 mouse embryo. Bottom, mxIF staining performed on the same tissue section. SMA, smooth muscle actin. **f**, Comparison of the spatial gene expression (DBiTplus) and protein expression (CODEX) for selected markers reveals concordant spatial localization across both modalities. **g**, CODEX-informed spot deconvolution of DBiTplus data. Cells were annotated by label transfer from the MOCA dataset using MaxFuse.

**Extended Data Fig. 3:**
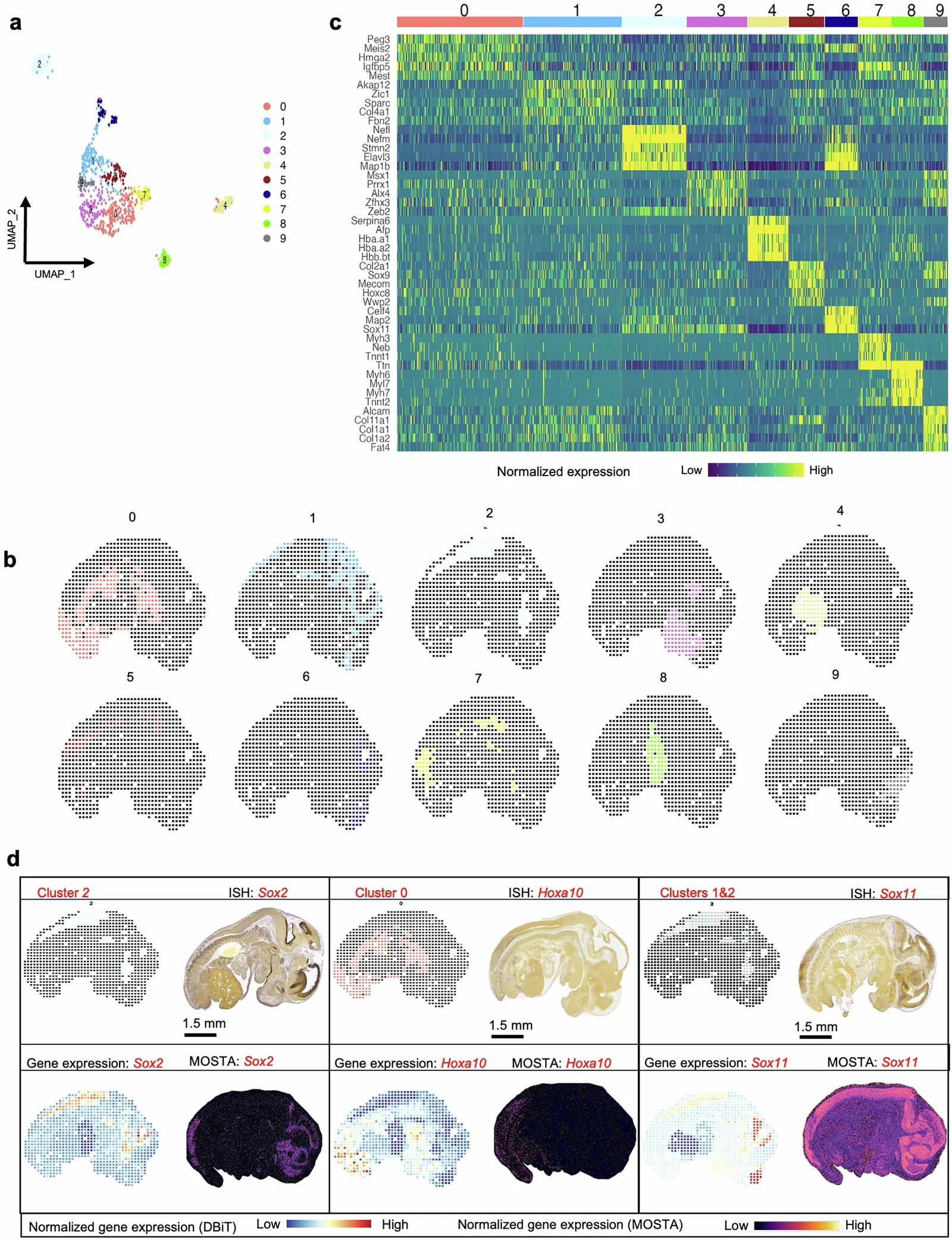
Spatial profiling of gene expression in FFPE mouse embryo using DBiTplus. **a** UMAP showing 10 distinct clusters of DBiTplus spots/spots. **b** Spatial distribution of the 10 clusters of DBiTplus spots. **c** Heat map showing the top 5 differentially expressed genes for each spatial cluster. **d** Spatial distribution of clusters 0, 1, and 2 alongside their principal defining genes. In-situ Hybridization (ISH) staining image from the Allen Mouse Brain Atlas (Developing Mouse Brain - Age: E13.5, Theiler Stage: TS21) and gene expression from STOmics Mouse Organogenesis Spatiotemporal Transcriptomic Atlas (MOSTA) dataset (E13.5_E1S1.MOSTA.h5ad). Scale bar 1.5 mm for Allen Mouse Brain Atlas images.

DBiTplus was applied to an E11 paraffin-embedded mouse embryo (Fig. 2a) and showed a strong correlation (*R* = 0.99) with the standard DBiT-seq workflow (on the adjacent section) with 27,884 overlapping genes. Each spot captured ~1,200 genes and 3,300 unique molecular identifiers (UMIs; Fig. 2b–d). The same section was stained with a 26-marker CODEX panel. Unsupervised clustering identified ten transcriptomic clusters, which closely aligned with anatomic structures and spatial protein patterns from the CODEX data (Fig. 2e and Extended Data Fig. 3a,b). Cluster 4 expressed liver markers (*Hba.a1*, *Hba.a2*, *Hbb.bt*, *Afp* and *Serpina6)*, while cluster 8 expressed embryonic heart markers (*Myh6*, *Myl7*, *Myh7* and *Tnnt2*; Extended Data Fig. 3c). mRNA and protein expression showed consistent spatial patterns for genes such as *Mki67*, *Nefl* and *Sox2* (Fig. 2f). Furthermore, spatial expression of *Sox2* (pluripotency, cluster 0) *Hoxa10* (limb muscle development, cluster 1) and *Sox11* (progenitor cell behavior regulation including neurogenesis, cluster 2) matched in situ hybridization data from the Allen Brain Atlas and the MOSTA dataset (Extended Data Fig. 3d). A trained support vector machine model enabled comprehensive cell-type annotation of the whole CODEX dataset (Supplementary Figs. 2 and 3). Integration of CODEX and DBiT-seq allowed deconvolution of DBiTplus spots into constituent cell types (Fig. 2g), outperforming TACCO (Supplementary Fig. 4). Using Seurat weighted-nearest-neighbor (WNN) methodology, further enhanced cell-type separation, clearly distinguishing epithelial cells from other cell types.

### Multimodal mapping of normal human lymph nodes

**Fig. 3:**
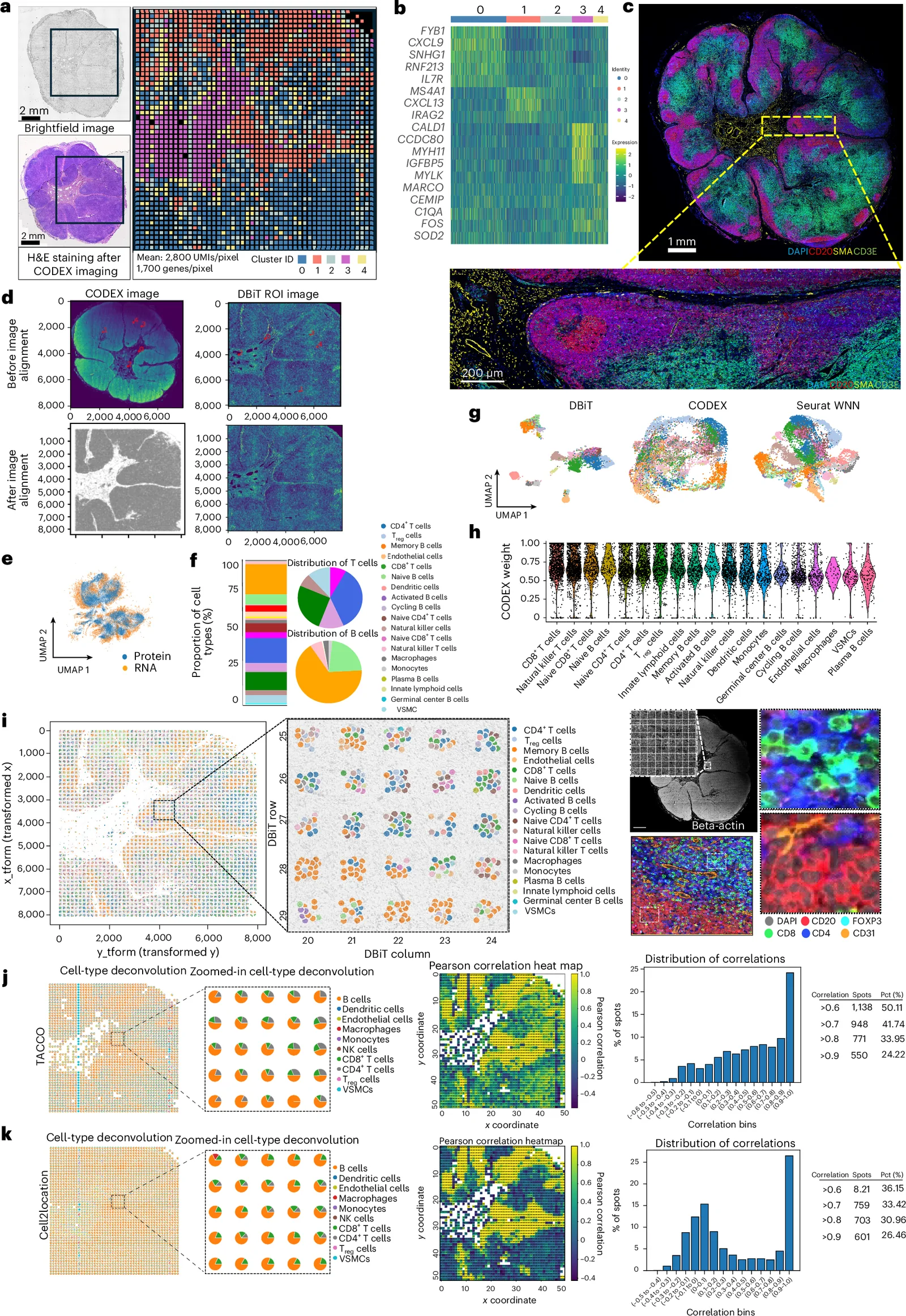
Spatial multi-omic profiling of FFPE human lymph node tissue section. **a**, Spatial transcriptomic profiling of an FFPE benign human lymph node section at 50-μm resolution using DBiTplus (*n* = 1). Left, brightfield and H&E images of the same tissue section after DBiTplus and CODEX. Right, spatial clustering of spots reveals five distinct transcriptomic regions that spatially correspond to distinct histological regions within the human lymph. **b**, Heat map showing the top five differentially expressed genes (DEGs) across spatial transcriptomic clusters. **c**, mxIF staining using CODEX on the same tissue section (*n* = 1). Cell-type markers CD20 (B cells), SMA (vasculature) and CD3E (T cells) are visualized. Bottom, high-resolution view of the boxed region. **d**, Image registration of CODEX and DBiTplus data using affine transformation and landmark alignment. **e**, UMAP projection of integrated protein (CODEX) and RNA (DBiTplus) modalities shows strong concordance following MaxFuse integration. **f**, Cell-type composition derived from CODEX-labeled cells within DBiTplus spots, visualized as bar plots (left) and pie charts (right) for T cells and B cells. **g**, UMAP plots of DBiTplus, CODEX and Seurat WNN-integrated datasets colored by cell-type annotation. **h**, Seurat WNN-derived modality weights for each cell type. The strongest weights were observed for T cell subtypes and the lowest were observed for B cell subtypes. These contributions are influenced by the composition of the CODEX marker panel. **i**, CODEX-informed spatial deconvolution of DBiTplus transcriptomic spots. Left, deconvolved spatial map, number of cells and cell types inferred from CODEX cell segmentation and cell-type annotation; middle: zoomed-in spot-level cell-type compositions; right, multiplexed image of the same region from CODEX (DAPI, CD20, CD8, CD4, FOXP3, CD31). Imprint of DBiTplus microfluidic channel on the tissue section observed in the beta-actin channel. **j**, Spatial deconvolution of DBiTplus data using TACCO. Left, deconvolved map; middle, spot-level pie chart visualization of cell types; right, correlation heat map and histogram of distribution of correlations. 50% of spots have Pearson correlation scores >0.6 when compared with ground truth (CODEX-informed spot-level cell-type deconvolution from **i**). **k**, Cell2location-based deconvolution and spatial mapping of cell types. Left to right, cell-type map, spot-level pie charts, correlation heat map and correlation score distribution. 36% of spots have Pearson correlation scores >0.6 when compared with ground truth (CODEX-informed spot-level cell-type deconvolution from **i**). VSMCs, vascular smooth muscle cells; T_reg_ cells, regulatory T cells; NK, natural killer cells; Pct, percentage.

**Extended Data Fig. 4:**
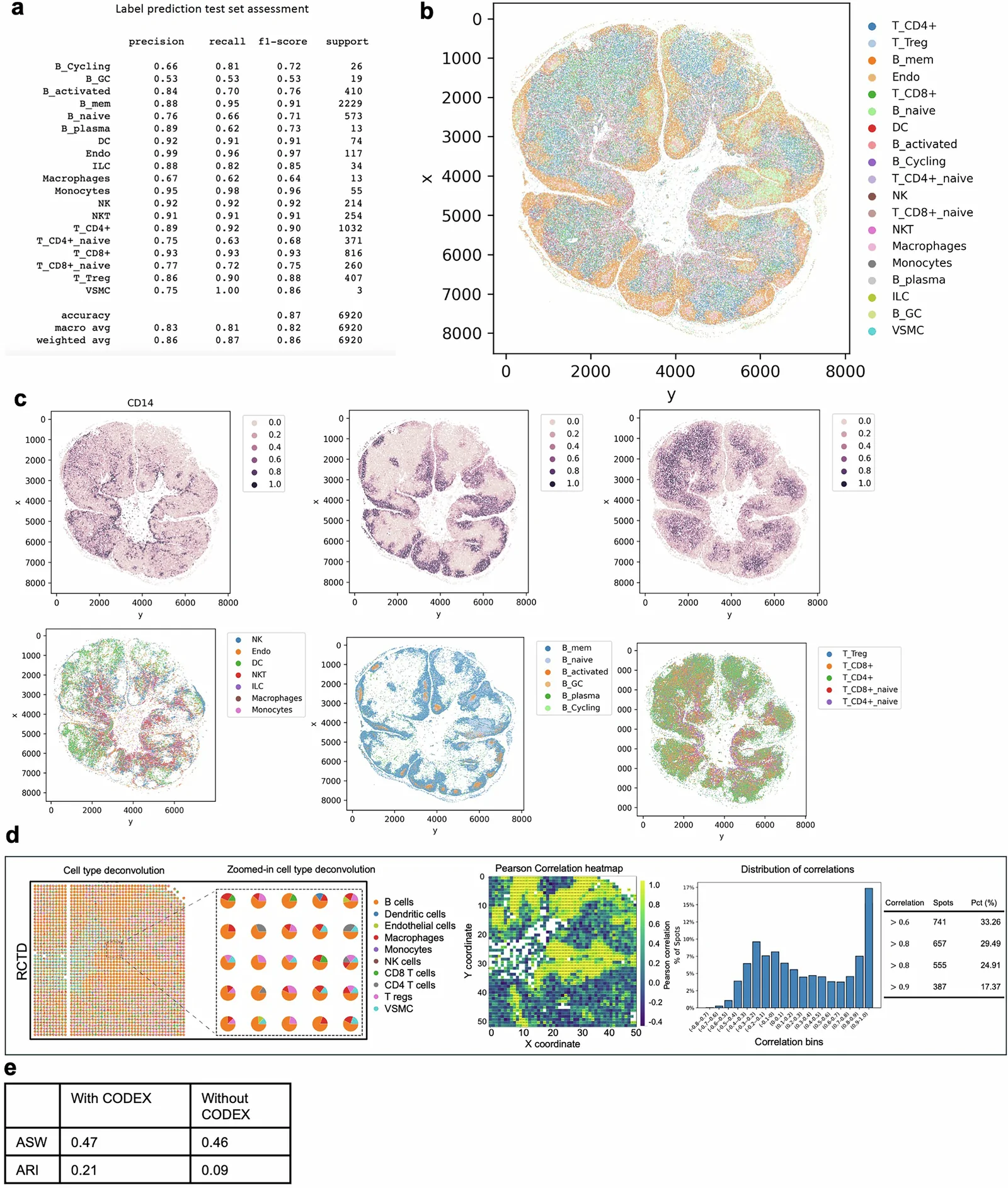
Evaluation of cell type classification and CODEX-informed deconvolution in FFPE human lymph node. **a** Label prediction metrics (precision, recall, F1-score, and support) for immune and stromal cell types from the integrated CODEX and single-cell RNA-seq dataset. Highest F1-scores were observed for endothelial cells, macrophages, dendritic cells, and multiple B cell subsets. **b** Spatial distribution of annotated cell types across the entire lymph node tissue. **c** Left: Spatial expression patterns of key lineage markers - CD14 (myeloid lineage), CD20 (B cells), and CD3ε (T cells) derived from the CODEX dataset. Right: spatial plots showing subtypes corresponding to each major lineage, including T cell subsets, B cell subsets and myeloid populations. **d** Spatial deconvolution results from RCTD. Left: whole-slide deconvolution maps; middle: spot-level pie chart visualization of cell types; right: correlation heat map and histogram of distribution of correlations. **e** Performance metrics for CODEX-informed versus uninformed deconvolution.

We applied DBiTplus to adjacent sections of a benign human lymph node, using 50-μm (Fig. 3) and 25-μm microfluidic devices, respectively (Supplementary Fig. 5). After cDNA retrieval, sections were stained with a 35-plex CODEX panel and H&E staining was done (Fig. 3a). Unsupervised clustering revealed five transcriptomic clusters, including B cells (*MS4A1*, cluster 1), smooth muscle cells in the medulla (*MYH11* and *CALD1*, cluster 3) and macrophages lining the medullary cords (*MARCO*, cluster 4; Fig. 3b,c). DBiTplus and CODEX images were co-registered, aligning the cellular-level data from the CODEX images with the spatial transcriptomic data from the DBiTplus spots (Fig. 3d). Uniform manifold approximation and projection (UMAP) embedding of the reference scRNA-seq and CODEX datasets using MaxFuse is shown in Fig. 3e. Endothelial cells had the highest f1-scores (for label-transfer accuracy), while germinal center B cells (GCB cells) had lowest scores (Extended Data Fig. 4a). Cell-type distributions matched known lymph node biology, with T cells and B cells predominating (Fig. 3f and Extended Data Fig. 4b,c). Joint embedding of DBiTplus and CODEX (Fig. 3g) and violin plots of the cell-type-specific modality weights from WNN analysis, showed that the CODEX (protein) contributed more for T cell-subtype identification, relative to DBiTplus (transcriptome), and the converse was observed for B cell subtypes such as GCB and plasma B cells (Fig. 3h). This can be explained by the marker composition of the CODEX panel, which included more T cell than B cell markers.

Each DBiTplus spot was subdivided into pure cell-type sub-spots, with cell-type identities assigned through MaxFuse label transfer and validated against CODEX markers (4′,6-diamidino-2-phenylindole (DAPI), CD20, CD8, CD4, FOXP3, CD31) from the same region of the tissue. The imprint of the microfluidic channels, visible in the β-actin channel, provided additional confirmation of spot locations. Three methods—TACCO (Optimal transport-based)^17^, Cell2location (Bayesian probabilistic model)^18^ and RCTD (Poisson regression-based deconvolution)^12^—were tested to benchmark spatial cell-type deconvolution, relative to the ground-truth CODEX-informed spot-level cell-type dataset (Fig. 3i). TACCO performed best, with 50% of spots showing Pearson scores > 0.6, versus 36% for Cell2location (Fig. 3j,k). Metrics for RCTD are shown in Extended Data Fig. 4d. Average silhouette width (ASW) scores, with or without CODEX (0.47 versus 0.46), were similar for the DBiTplus transcriptome dataset, indicating comparable clustering structure in the DBiTplus dataset alone. However, adjusted Rand index (ARI) scores improved from 0.09 to 0.21 with CODEX guidance, reflecting more accurate cell-type assignment (Extended Data Fig. 4e).

**Extended Data Fig. 5:**
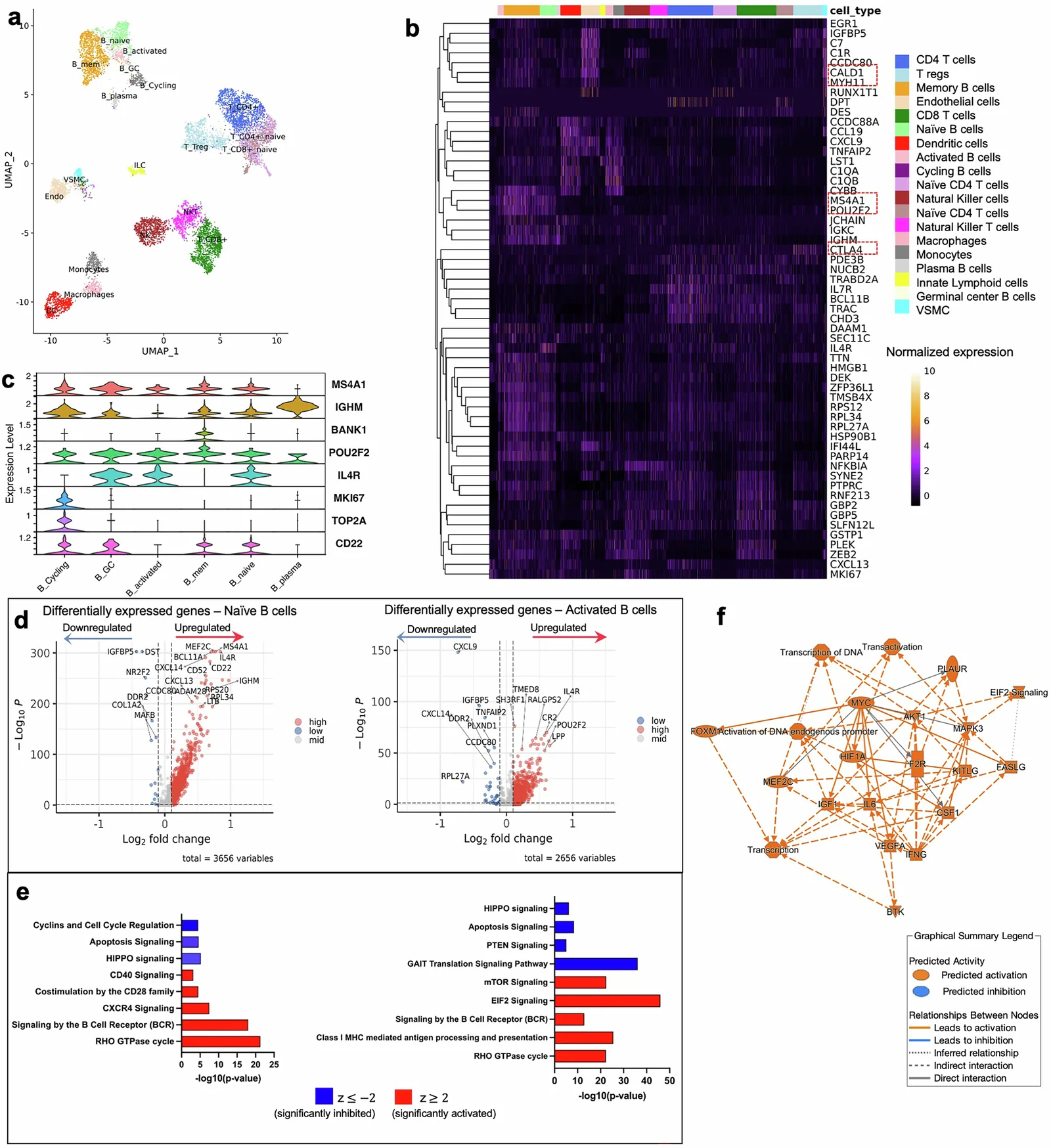
Transcriptomic profiling and pathway analysis of B cells populations from FFPE human lymph node. **a** UMAP embedding from DBiTplus following CODEX-guided deconvolution reveals distinct immune and stromal cell populations, including naive B cells, activated B cells, cycling B cells, memory B cells, plasma B cells, and T cell subsets. **b** Heat map of differentially expressed genes across annotated cell types. **c** Violin plots of representative (canonical) marker genes for various B cell subtypes. **d** Volcano plots showing differentially expressed genes in naive (left) and activated (right) B cells compared to all other cells. Differential gene expression computed from two-sided Wilcoxon rank-sum test, adjusted P value on the basis of Bonferroni correction) **e** Pathway analysis comparing enriched pathways between naïve B cells and activated B cells. **f** Ingenuity Pathway Analysis (IPA) graphical summary showing predicated canonical pathways, upstream regulators and transcriptional and translational programs in activated B cells.

UMAP of the DBiTplus (transcriptome) revealed diverse immune (B cell and T cell subsets) and stromal populations (Extended Data Fig. 5a). Cell-type-specific markers *MS4A1* (canonical B cell marker), *IL7R* (naive CD4^+^ T cells), *CTLA4* (regulatory T cells) and *CALD1* and *MYH11* (endothelial and vascular smooth muscle cells) distinguished immune subsets (Extended Data Fig. 5b). Exploring B cell activation and maturation, violin plots revealed distinct transcriptional profiles—*MS4A1*, *POU2F2* and *CD22*—broadly expressed across naive B cells, memory B cells and activated B cell populations, but downregulated in plasma B cells with concurrent upregulation of *IGHM*, consistent with plasma B cell maturation. *IL4R* was enriched in GCB/naive/activated B cell subsets, indicating activation or a poised state for activation, while *MKI67* and *TOP2A* were upregulated in cycling B cells, indicating active proliferation. These patterns validate B cell-subtype annotations and capture functional transitions from naive to proliferative and terminally differentiated states (Extended Data Fig. 5c).

To delineate transcriptional differences between antigen-inexperienced and antigen-experienced B cells, we compared naive and activated B cells using differential gene expression and pathway analysis. Naive B cells upregulated *MS4A1*, *IGHM*, *IL4R* and *POU2F2*, consistent with a resting phenotype capable of antigen sensing and homing. Correspondingly, B cell antigen receptor (BCR) signaling, CXCR4 signaling and RHO GTPase cycle pathways were upregulated, whereas apoptosis signaling was downregulated, underscoring reflecting migratory readiness and pro-survival status. In contrast, activated B cells upregulated *IL4R*, *CD2*, *POU2F2* and *LPP* genes and EIF2 signaling, BCR signaling and antigen presentation pathways, consistent with immune activation and survival (Extended Data Fig. 5d–f). These findings underscore functional divergence between naive and activated B cells and validate the resolution of our spatial transcriptomic profiling.

### Evaluating RNA–protein concordance in DBiTplus

**Extended Data Fig. 6:**
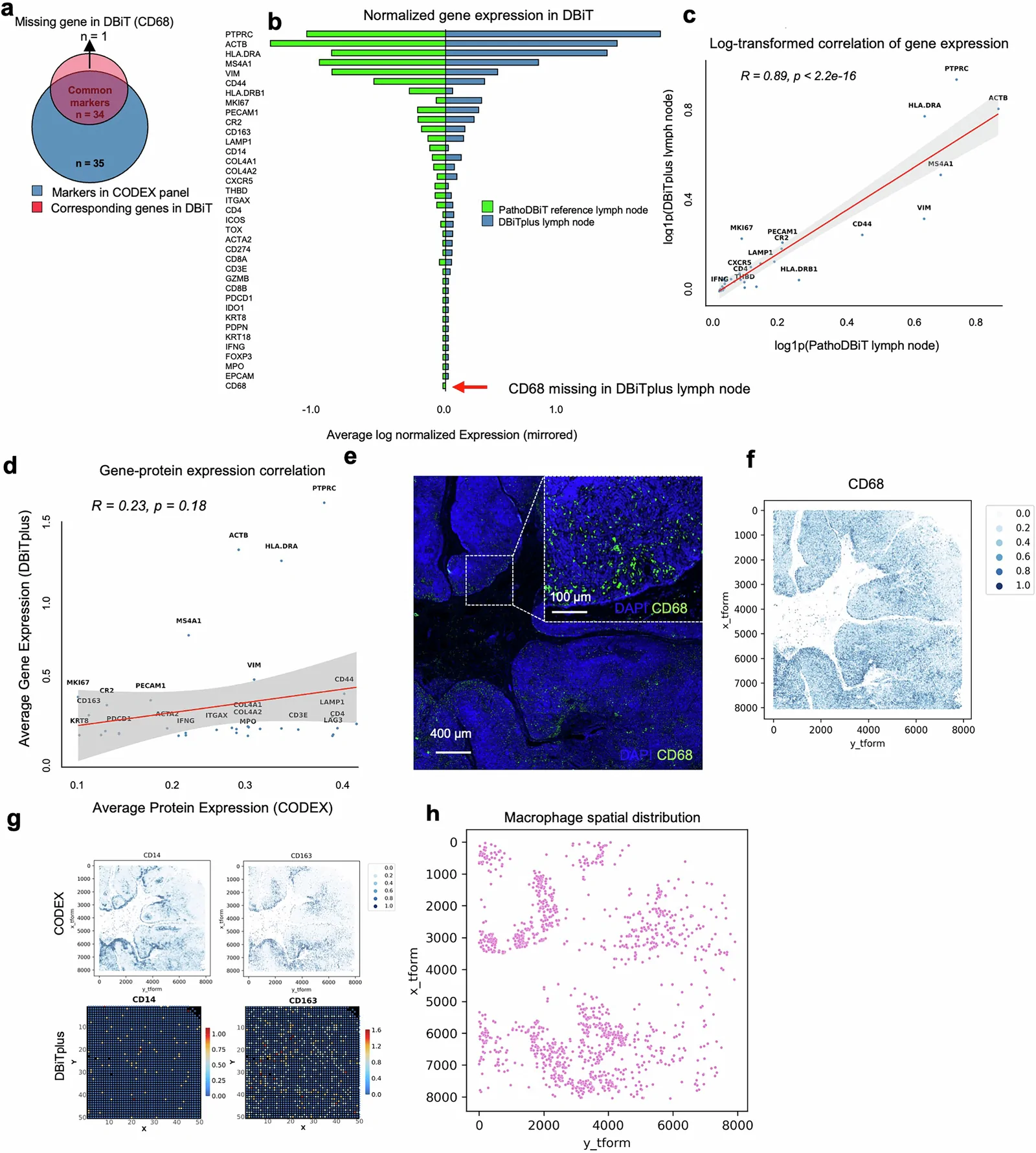
Concordance of transcriptomic and protein-level data across DBiTplus and CODEX platforms, with spatial analysis of macrophages. **a** Venn diagram comparing 35 markers in the CODEX panel with corresponding gene expression data from DBiTplus. CD68 is the only protein marker without a corresponding gene transcript detected in DBiTplus. **b** Bar plot showing normalized expression of matched genes between DBiTplus lymph node (blue) and a Patho-DBiT reference lymph node (green). **c** Scatter plot showing correlation of log-transformed gene expression between DBiTplus (n = 1) and Patho-DBiT reference lymph node sample (n = 1). Each dot represents a gene, the red line indicates linear regression fits and the shaded gray area indicates the 95% confidence interval for the regression line. The correlation analysis is from a two-sided Pearson correlation test. Strong concordance observed (Pearson R = 0.89, *p* < 2.2 × 10^−16^). **d** Correlation between protein expression (CODEX) and gene expression (DBiTplus) across matched markers. Each dot represents a gene, the red line indicates linear regression fits and the shaded gray area indicates the 95% confidence interval for the regression line. Correlation analysis from a two-sided Pearson correlation test. Weak but positive association (R = 0.23, *p* = 0.18). **e** Multiplexed CODEX image showing CD68^+^ macrophages within the lymph node architecture (blue: DAPI, green: CD68). Zoomed-in region highlights macrophage-rich zones. **f** Spatial feature plot showing the expression pattern of CD68 in the CODEX dataset. **g** Comparative visualization of CD14 and CD163 expression in both CODEX (top row, protein) and DBiTplus (bottom row, RNA), showing similar spatial enrichment patterns. **h** Spatial distribution of macrophages as identified from CODEX-guided DBiTplus cell type deconvolution.

We evaluated RNA–protein concordance in DBiTplus, comparing spatial transcriptomics with CODEX imaging. This is important because the relationship between protein levels and their coding transcripts can be discordant, influenced by spatial and temporal mRNA variations and the protein biosynthesis machinery^19^. Of the 35 markers in the human lymph node CODEX panel, *CD68* was the only gene transcript missing (Extended Data Fig. 6a). As validation, we compared our normalized gene expression to a reference lymph node dataset from Bai et al.^13^ (also using Patho-DBiT), noting generally consistent gene expression levels (*R* = 0.89, *P* < 2.2 × 10^−16^), particularly for highly abundant transcripts like *PTPRC, ACTB and HLA.DRA*. Low-expressing genes, such as *GZMB* and *IFNG*, were present at low levels in both datasets. Interestingly, *CD68* transcripts were detectable in the reference dataset (Extended Data Fig. 6b,c). Given the documented discrepancies between transcript and protein expression in immune cells, we evaluated the correlation between average normalized mRNA and protein levels in our dataset^20^. As expected, we observed minimal correlation (*R* = 0.23, *P* = 0.18), consistent with prior findings in peripheral blood mononuclear cells, particularly for T cell markers such as *CD4* (Extended Data Fig. 6d). CD68 protein expression was clearly visible in the CODEX images (Extended Data Fig. 6e,f), and other macrophage-associated markers such as CD14 and CD163 were detected in both modalities (Extended Data Fig. 6g,h). These findings underscore the strength of DBiTplus in co-mapping RNAs and proteins on the same tissue section, allowing for cross-validation and enhancing confidence in cell-type annotation and spatial localization.

### Assessment of mxIF imaging and H&E staining

Assessment of CODEX data from the DBiTplus workflow revealed staining quality comparable to control lymph node sections (two sections away from the Fig. 3 sample), with strong correlations (Pearson > 0.85) for key cell-type markers including CD3ε, CD20 and podoplanin (Supplementary Figs. 6a,b and 7). H&E staining after DBiTplus, evaluated against standard H&E and post-CODEX H&E (no prior DBiT), showed partially diminished nuclear detail, but still allowed trained pathologists to identify major cell types based on cellular morphology and spatial context (Supplementary Fig. 6c). This suggests the utility of these H&E images for inferring single-cell spatial gene expression in the dead space regions of the microfluidic channels using tools like iStar^21^. It is worth noting that post-CODEX H&E imaging can be challenging due to potential tissue damage during the flow cell removal, which may compromise reliable histological analysis.

### Multimodal mapping of the progression of MZL using DBiTplus

**Fig. 4:**
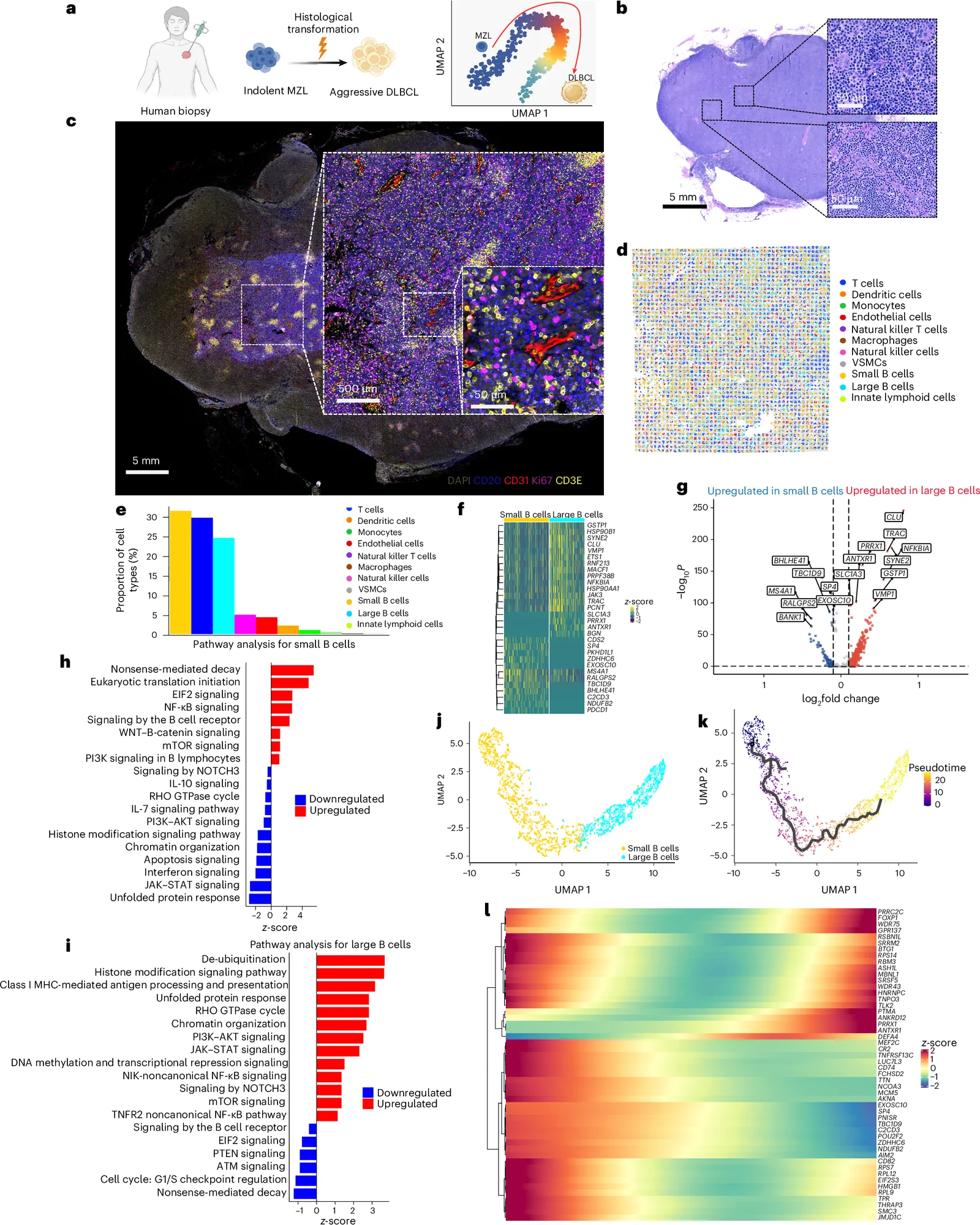
Spatial multi-omic characterization of MZL. **a**, Schematic overview of the study design. Created with BioRender.com. **b**, H&E staining of the FFPE lymph node section showing both small and large malignant lymphocytes (*n* = 1). **c**, Multiplexed CODEX imaging of the same tissue section (*n* = 1), showing CD20, CD3E, CD31 and Ki67. Zoomed-in image showing detailed views of proliferative tumor zones and vasculature. **d**, Spatial cell-type mapping using CODEX-guided DBiTplus transcriptomics. **e**, Bar plot of cell-type proportions across the lymphoma section dominated by B cells (small and large) and T cells. **f**, Heat map of DEGs between small B cells (MZL-like) and large B cells. **g**, Volcano plot highlighting the DEGs in small B cells and large B cells, with key transformation-associated genes labeled. Each dot represents a specific gene, with upregulation in small and large B cells colored in blue and red, respectively. (Differential gene expression computed from two-sided Wilcoxon rank-sum test, adjusted *P* value on the basis of Bonferroni correction.) **h**,**i**, Pathway enrichment analysis of small B cells (**h**) and large B cells (**i**) showing distinct pathway activation profiles during transformation. Small B cells are enriched for signaling pathways such as BCR and NF-kB signaling, while large B cells show enrichment for PI3K–AKT and NOTCH signaling pathways. MHC, major histocompatibility complex. **j**, Monocle 3 UMAP embedding colored by B cell subtype shows a continuum from small to large B cells. **k**, Pseudotime trajectory analysis reveals a dynamic transcriptional progression along the transformation axis. **l**, Heat map of gene expression dynamics along pseudotime highlights upregulation of transformation-related genes during the evolution of low-grade to high-grade lymphoma.

To investigate lymphoma progression, we profiled a marginal zone lymphoma (MZL) sample that showed increased large cell populations, high proliferation index and clinical progression without overt histological transformation (Fig. 4a). Unlike healthy lymph nodes with well-defined follicular structure, MZL is characterized by effacement of normal tissue architecture through the infiltration of large, atypical B cells and proliferation of small to medium-sized lymphocytes with irregular nuclei, condensed chromatin and scant cytoplasm (Supplementary Fig. 8a and Fig. 4b). Clinical immunohistochemical staining was performed for relevant clinical markers (Supplementary Fig. 8b). Interestingly, we observed robust follicular dendritic cell networks marked by CD21, with clusters of CD3^+^ and CD5^+^ T cells.

**Extended Data Fig. 7:**
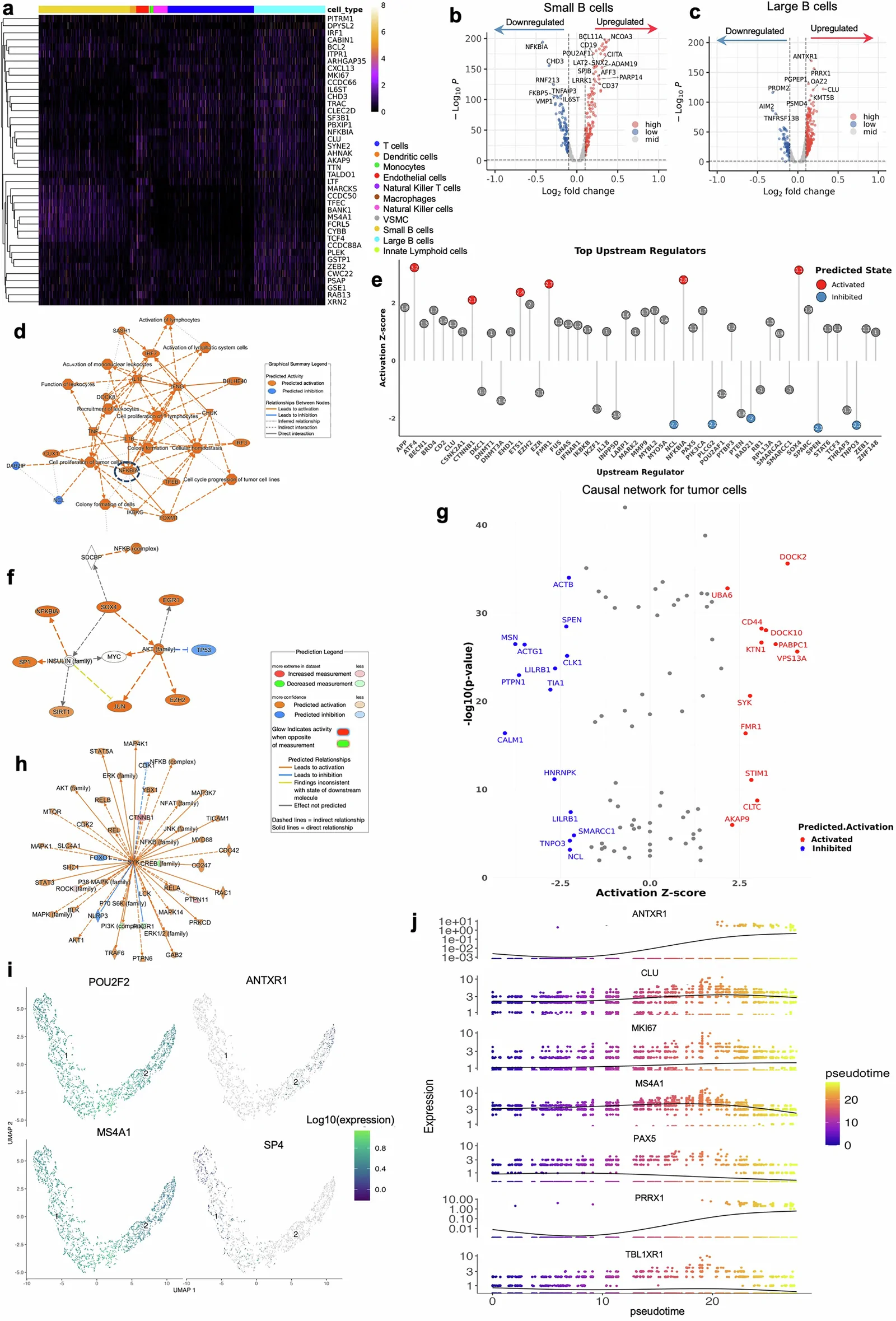
Transcriptional and regulatory landscape of B cell transformation. a Heat map of top differentially expressed genes across annotated cell types from the spatial transcriptomics dataset. **b**–**c**, Volcano plots highlighting differentially expressed genes in small B cells (**b**) and large B cells (**c**) compared to all other cell types. Differential gene expression computed from two-sided Wilcoxon rank-sum test, adjusted P value on the basis of Bonferroni correction) **d**, IPA-inferred upstream regulator network predicting TF-driven control over gene expression changes in tumor B cells. **e** Activation z-scores of top predicted upstream regulators of genes from pathway analysis. **f** Causal network diagram centered on MYC, highlighting upstream regulation from *SOX4* through the AKT family. **g** Activation z-scores vs. –log_10_(p-values) from causal network analysis showing predicted activators (red) and inhibitors (blue). z-score ≤ -2 (significantly inhibited) and z-score ≥ 2 (significantly activated). P-values calculated using the right-tailed FET (Fisher’s Exact Test). **h** Expanded regulatory network involving of SYK, a key upstream regulator of multiple genes in lymphoma transformation. **i** Monocle3 UMAP embedding showing four key genes. **j** Pseudotime heat map trajectories of selected genes reveal temporal dynamics of MZL.

A 2.5-mm × 2.5-mm region was profiled with DBiTplus (25-μm microfluidic device) and CODEX (44-marker panel) on the same tissue section. CODEX revealed extensive CD20^+^ Ki67^+^ proliferating B cells (Fig. 4c). B cells were classified as large or small B cells based on size and CD20 intensity. Integration of protein and RNA data revealed concordant expression of CD31, CD4 and TIGIT in the joint UMAP embedding (Supplementary Fig. 8c). The tumor microenvironment (TME) is composed primarily of malignant B cells (both large and small, ~55%) and T cells (~30%), consistent with mxIF and clinical immunohistochemical images for CD20 and CD3 (Fig. 4d,e). The transcriptional profile of large B cells was consistent with biological evolution with upregulated genes like *IL6ST* (interleukin-6 signaling), *CLU* (apoptosis inhibition) and *SYNE2, AHNAK* and *MARCKSL1* (protein synthesis and cell growth)^22,23^ indicating increased survival, and dysregulated inflammatory signaling (Extended Data Fig. 7a). DEG analysis between small and large B cells showed upregulation of *NFKBIA*, suggesting potential dysregulation of the frequently overactive nuclear factor (NF)-κB pathway, as well as upregulation of *PPRX1, ANTRX1* and *VMP1*, associated with migration/metastasis, PI3K–AKT–mTOR signaling and autophagy, respectively, in large B cells^24–26^. Small B cells showed upregulation of *PKHD1L1* and *EXOSC10*, associated with B cell lymphoma growth, activation and DNA repair^27,28^ (Fig. 4f,g and Extended Data Fig. 7b,c). Pathway analysis highlighted the upregulation of the NF-κB signaling pathways and WNT–β-catenin signaling, and a downregulation of epigenetic and chromatin organization, PI3K/AKT and BCR signaling in small B cells, while large B cells revealed upregulation in noncanonical NF-κB signaling, chromatin and histone modification and PI3K–AKT signaling pathways (Fig. 4h,i and Extended Data Fig. 7d).

Upstream regulator analysis of DEGs in large B cells identified *SOX4* (a MYC upstream regulator through ATK family and TP53 inhibitor) and downregulation of *RAD21* (cohesion complex gene), whose reduced expression in diffuse large B cell lymphoma (DLBCL) correlates with decreased survival, suggesting underlying pro-tumor survival mechanisms^29^ (Extended Data Fig. 7e,f). Causal network analysis (which identifies upstream regulators of gene expression in large B cells) highlighted *SYK*, an SRC family kinase involved in BCR and PI3K–AKT signaling, as a key master regulator. Additional regulators included upregulated *DOCK2* (proliferation regulator through *RAC* and *ERK* activation in B cell lymphomas) and downregulated *SPEN* (Notch signaling pathway regulator; Extended Data Fig. 7g,h), These transcriptional shifts offer insight into the biological programs underlying biological progression of MZL.

### Loss of B cell identity and activation of oncogenic programs characterize the progression of MZL along the pseudotime axis

We performed pseudotime analysis on the deconvoluted data of small and large B cell transcriptomes using Monocle 3 (ref. ^15^) and found small and large B cells on a differentiation continuum, revealing dynamic transcriptional changes across MZL progression (Fig. 4j,k). UMAP of B cell markers showed that both small and large B cells expressed *POU2F2* and *MS4A1*, whereas *ANTXR1* and *SP4* were restricted to large and small B cells, respectively (Extended Data Fig. 7i). *ANTXR1* is known to be elevated in the progression of several cancers and has recently been identified as a therapeutic target in DLBCL^30^. *PTMA*, *PRRX1*, *FOXP1* and *ASH1L* increased, reflecting enhanced proliferation and transcriptional and epigenetic programs, while B cell-specific genes *MS4A1* and *PAX5* reduced in pseudotime, consistent with the molecular evolution of indolent MZL (Extended Data Fig. 7j and Fig. 4l). These findings suggest that MZL progression (with the increased presence of large B cells as well as aggressive clinical features) is driven by a coordinated loss of B cell identity and immune function, coupled with the upregulation of proliferation, transcriptional reprogramming and epigenetic remodeling pathways, characteristic of aggressive lymphoma transformation.

### Multimodal mapping of the transformation of CLL to DLBCL using DBiTplus

**Fig. 5:**
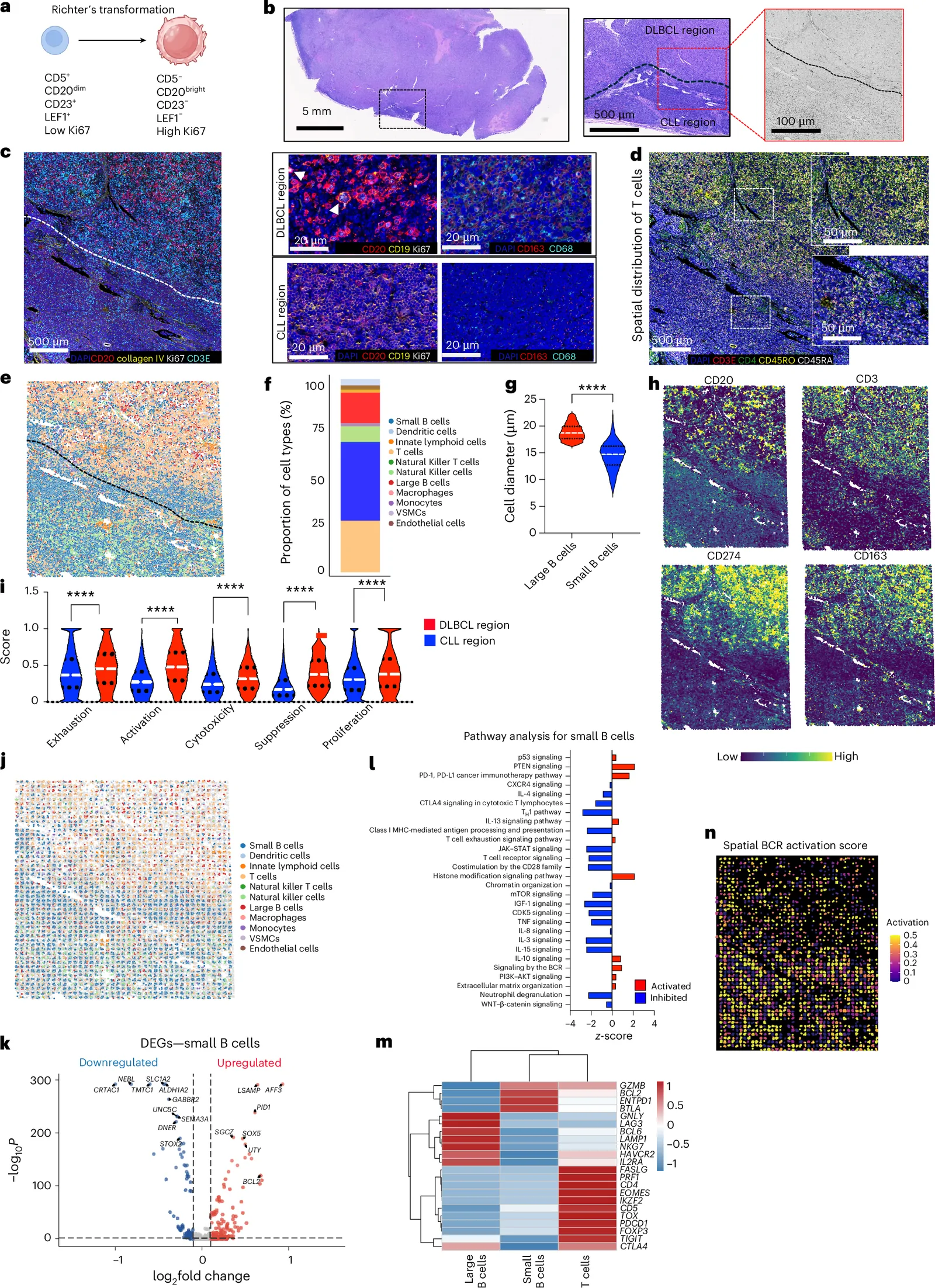
Spatial multi-omics characterization of Richter’s transformation from CLL to DLBCL. **a**, Schematic overview of transformation from CLL (CD5^+^CD20^dim^CD23^+^LEF1^+^Ki67^low^) to DLBCL (CD5^+^CD20^bright^CD23^+^LEF1^−^Ki67^high^). Created with BioRender.com. **b**, H&E staining of FFPE lymph nodes showing histologically distinct regions of CLL and DLBCL (*n* = 1). Zoomed-in region highlighting the transition between the two regions. Right, brightfield image of the DBiTplus-barcoded region. **c**, mxIF imaging (using CellScape) of the same tissue section showing CD20, CD3E, Ki67, COL4A1 and DAPI staining (*n* = 1). The regions below and above the dashed line represent the CLL and DLBCL regions, respectively. Zoomed-in region demonstrating differences in proliferation and immune infiltration between DLBCL and CLL regions. Arrowheads point to highly proliferative large B cells. **d**, Spatial distribution of T cell markers (CD4, CD45RO, CD3E and CD45RA) in DLBCL versus CLL regions, highlighting increased T cell infiltration in transformed zones. **e**, Spatial cell-type deconvolution using CellScape-guided DBiTplus transcriptomics reveals regional differences in immune cell composition across the tissue section. Cell-type annotation legend shown in **f**. **f**, Bar plot showing cell-type proportions; large B cells dominate DLBCL regions, while small B cells are enriched in CLL regions. **g**, Violin plot comparing large B cell and small B cell sizes. Significance level was calculated with unpaired two-tailed Welch *t*-test, ****P < 0.0001. (*n*_large B cells_ = 6,140 and *n*_small B cells_ = 15,707). The shape of the violin represents the kernel density estimate of the data distribution. The solid white dashed line indicates the median, and the upper and lower black dotted lines indicate the 25th and 75th percentiles. The full vertical extent of the violin represents the range of the data. **h**, Spatial gradient expression of selected protein markers (CD20, CD3, CD274/PD-L1, CD163) highlights differences in tumor and immune cell distribution across the tissue. **i**, Violin plot of the quantification of functional immune signatures (exhaustion, activation, cytotoxicity, suppression and proliferation) comparing DLBCL and CLL regions. Significance level was calculated with a two-tailed Mann–Whitney test, and ****P < 0.0001, (*n*_CLL_ = 28,331 and *n*_DLBCL_ = 10,336). The shape of the violin represents the kernel density estimate of the data distribution. The solid white dashed line indicates the median, and the upper and lower black dotted lines indicate the 25th (first quartile, Q_1_) and 75th (3rd quartile, Q3) percentiles. The full vertical extent of the violin represents the range of the data, **j**, Spatial spot-level cell-type deconvolution mapping of lymphoma tissue. **k**, Volcano plot showing DEGs in the small cells. Each dot represents a specific gene, colored in blue (downregulated) and red (upregulated), respectively. (Differential gene expression computed from two-sided Wilcoxon rank-sum test, adjusted *P* value on the basis of Bonferroni correction.) **l**, Pathway analysis of small B cells. *z*-score is computed and used to reflect the predicted activation level. **m**, Heat map showing differential expression of immune checkpoint and cytotoxicity genes across large B cells, small B cells and T cells. **n**, Spatial BCR activation score map derived from DBiTplus transcriptomics, revealing localized activation patterns across the tissue section.

**Extended Data Fig. 8:**
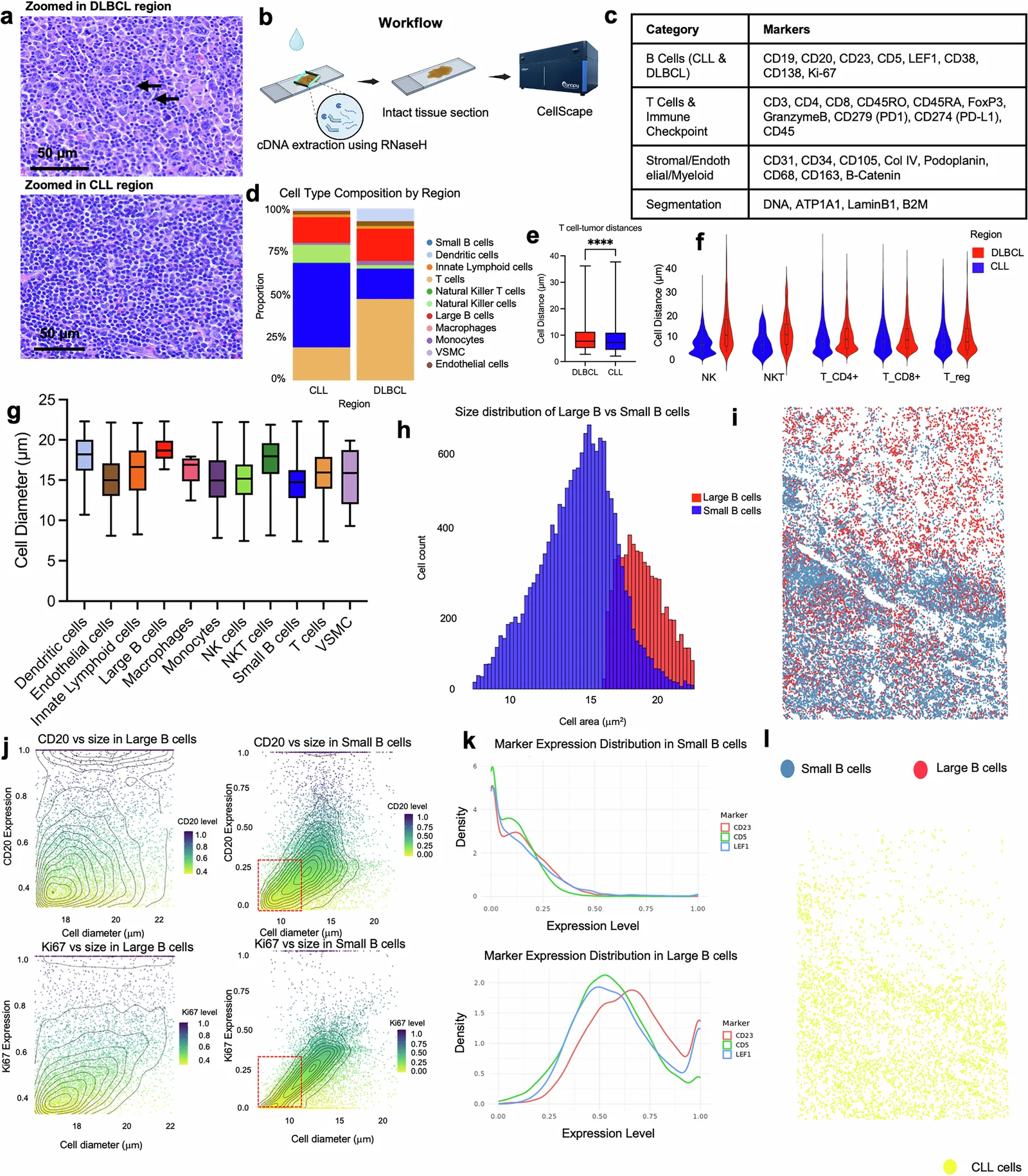
Spatial proteomic profiling of Richter’s Transformation. **a** Representative high-resolution images of DLBCL (top) and CLL (bottom) regions showing differences in cell size (n = 1). **b** Schematic of the DBiTplus workflow using CellScape on an FFPE tissue section. Created with BioRender.com. **c** Protein marker panel used in CellScape, including immune, stromal, and segmentation markers. **d** Stacked barplot showing cell type composition in CLL and DLBCL regions. **e** Boxplot where the central line represents the median, the bottom and upper edges of the box indicate the first and third quartiles, and the whiskers extend to the most extreme data points within 1.5 times the interquartile range (IQR) from the box edge for the quantification of average T cell-tumor cell distances in DLBCL compared to CLL. Computed using a two-tailed Mann-Whitney test (**** represents p < 0.0001, n_CLL_ = 5,525 and n_DLBCL_ = 4,924). Median distance 7.715 µm and 7.292 µm, respectively for DLBCL and CLL. **f** Violin plots of the distance of T cell subtypes from tumor cells in DLBCL versus CLL regions. Violin plots show the median (center line), the first and third quartiles (box limits) and 1.5x interquartile range (whiskers). **g** Boxplot, where the central line represents the median, the bottom and upper edges of the box indicate the first and third quartiles, and the whiskers extend to the most extreme data points within 1.5 times the interquartile range (IQR) from the box edge showing size distribution across all major annotated cell types. **h** Histogram of cell size distributions comparing large B cells (DLBCL) and small B cells (CLL). Histogram bin width of 0.25 **i** Spatial maps showing localization of large B cells (red) and small B cells (blue) within the tissue section (left), and CLL cells only (right). **j**, 2D density scatter plot showing CD20 and Ki67 expression as a function of cell size in large B cells (left) versus other B cells (right). Small B cells < 300 in size and < 0.30 in normalized Ki67 expression were annotated as CLL-like cells. **k**, Density distribution plots of marker expression (CD23, CD5, LEF1) in small (top) and large B cells (bottom). **l** Spatial maps showing localization of CLL-like cells.

Richter’s transformation is the evolution of chronic lymphocytic leukemia (CLL) into aggressive lymphomas, often DLBCL, although rare cases involve Hodgkin or T cell lymphomas^31^. Recent multi-omics studies have identified distinct Richter’s transformation subtypes, clonal origins and high-risk genomic features^32^. Most DLBCL-Richter’s transformation cases (~80%) are clonally related to the original CLL, representing true transformations, with poor prognosis (median overall survival <1 year), although de novo DLBCL can occur in individuals with concurrent CLL^33^. We profiled a rare case of concurrent CLL and Richter’s transformation-DLBCL within the same lymph node, providing a unique spatially contiguous context to study Richter’s transformation (Fig. 5a). H&E staining revealed a region of densely packed small monomorphic cells transitioning into a sheet of large pleomorphic cells with open chromatin corresponding to the CLL and DLBCL regions, respectively (Fig. 5b and Extended Data Fig. 8a). We applied DBiTplus to 5-µm-thick sections followed by imaging with a 30-marker panel (using CellScape, with relevant CLL/DLBCL markers such as CD5, LEF1 and CD20). An adjacent section was also imaged as a quality-control measure (Extended Data Fig. 8b,c). mxIF imaging revealed proliferating CD20^bright^Ki67^high^ large B cells (DLBCL region), contrasting with the CD20^dim^Ki67^low^ small B cells (CLL region), as well as a higher density of T cells within the DLBCL region (Fig. 5c,d). Leveraging the distinct features of CLL and DLBCL (CD20^dim^Ki67^low^ versus CD20^bright^Ki67^high^), B cells were initially classified as large or small based on size, and the remaining cells in the TME, annotated using MaxFuse (Fig. 5e), with B cells making up ~60% of all cells (Fig. 5f). T cell infiltration was higher in DLBCL than CLL, with small B cells, on average, located closer to the nearest T cell compared to large B cells (Extended Data Fig. 8d–f). Large B cells had the largest diameters of ~20 μm (Fig. 5g and Extended Data Fig. 8g,h). Some overlap of large B cells in CLL and small B cells in DLBCL suggested a gradual transition between the two regions (Extended Data Fig. 8i,l). To refine CLL cell classification, we applied a gating strategy based on cell with size and normalized CD20 and Ki67 expression (Extended Data Fig. 8j,k).

**Extended Data Fig. 9:**
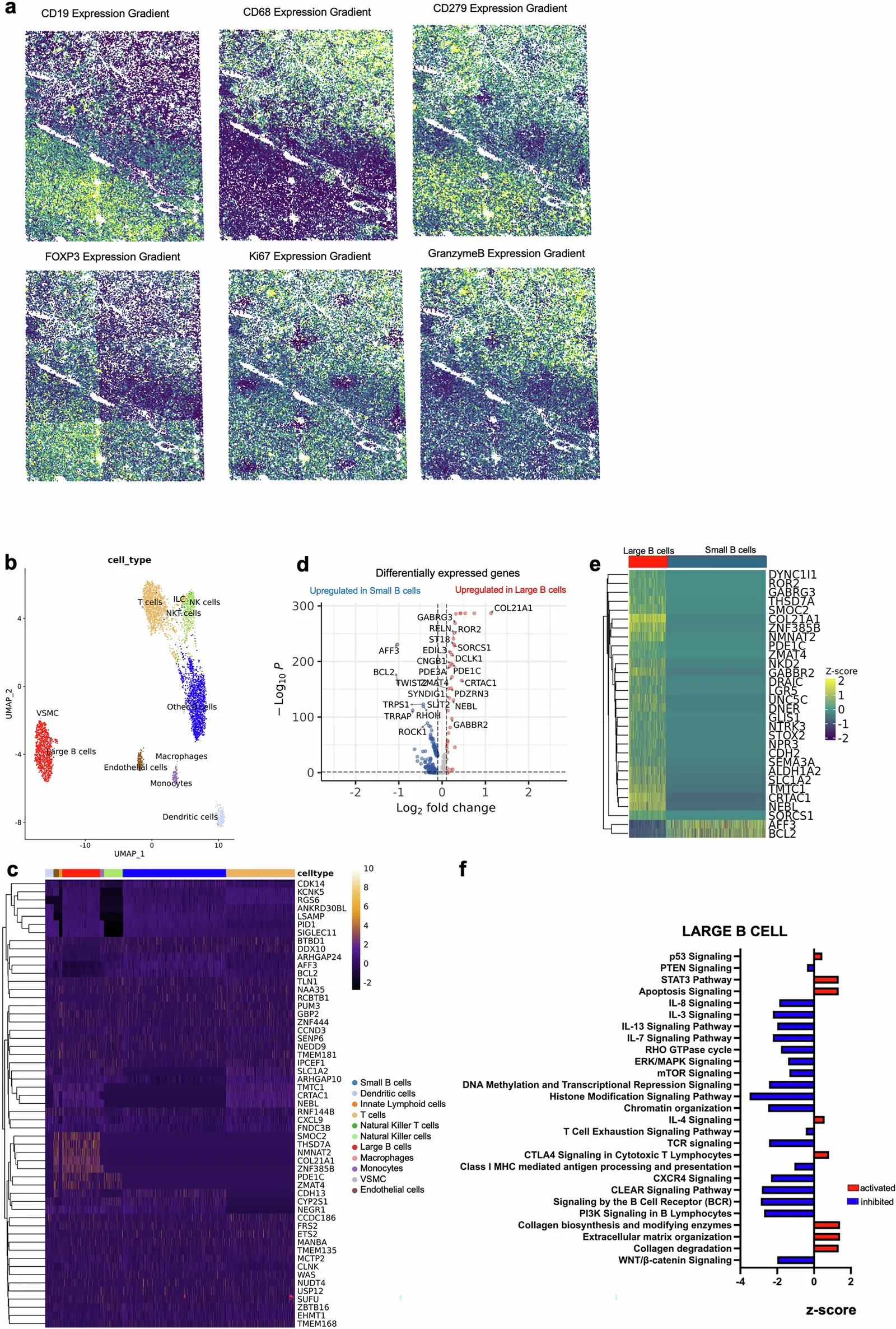
Spatial gradient expression and molecular features of Richter’s Transformation. **a** Spatial expression gradients for representative protein markers across the tissue section reveal distinct spatial localization (within CLL or DLBCL regions) patterns of B cells (CD19), macrophages (CD68), immune checkpoints (CD279/PD-1, FOXP3), proliferating cells (Ki67), and cytotoxic activity (GranzymeB). **b** UMAP plot showing unsupervised clustering of annotated cell type across the lymphoma tissue. **c** Heat map of top differentially expressed genes across all cell types. **d** Volcano plot showing DEGs between large B cells (DLBCL) and small B cells (CLL), with key upregulated genes in each population highlighted. Differential gene expression computed from two-sided Wilcoxon rank-sum test, adjusted P value on the basis of Bonferroni correction **e** Heat map of top 30 DEGs distinguishing large B cells from small B cells. **f** Ingenuity Pathway enrichment analysis of large B cells.

Aberrant immune regulation contributes to CLL initiation and progression, and Richter’s transformation is associated with increased FOXP3^+^ regulatory T cells and CD163^+^ macrophages, and elevated LAG-3 compared to de novo DLBCL^34,35^. Spatial gradient plots revealed enrichment of CD163^+^ macrophages, CD274 (PD-L1) and CD20^+^ cells in DLBCL regions (Fig. 5h), indicating an immunologically active yet suppressed microenvironment. Regional scores for activation (CD38, CD45RO), cytotoxicity (granzyme B, CD56, CD8), proliferation (Ki67), immunosuppression (CD274, FOXP3, CD163) and exhaustion (CD279) using multiplexed imaging data (Fig. 5i and Extended Data Fig. 9) were all higher in DLBCL than CLL regions (two-tailed Mann–Whitney test, ****P < 0.0001), mirroring the distribution of CD8^+^ T cells with highest exhaustion and large B cells, macrophages and regulatory T cells showing highest immunosuppression scores within the TME. This aligns with reports of aberrant PD-1^+^ neoplastic B cells and increased infiltration of FOXP3^+^ T cells and CD163^+^ macrophages in Richter’s transformation versus CLL, potentially influencing responses to immune checkpoint blockade. Additionally, CLL cells have been shown to overexpress PD-L1, which engages PD-1 on T cells, promoting immune tolerance through downstream inhibitory signaling^36^.

### Spatial transcriptomic landscape of Richter’s transformation

Distinct genetic and transcriptional alterations characterize Richter’s transformation, including *TP53* and *CDKN2A* deletions, *NOTCH1* gain-of-function mutations and *c-MYC* hyperactivation^37^. UMAP and spatial clustering revealed clear separation of cell types (Extended Data Fig. 9b and Fig. 5j). Large B cells uniquely expressed *ROR2*, *SMOC2* and *PDE1C*, implicated in proliferation, migration and regulation of the PI3K–Akt pathway, while small B cells overexpressed anti-apoptotic *BCL2*, consistent with their more indolent and chronic nature, and *AFF3*, suggesting IGHV-mutated CLL (Extended Data Fig. 9c–e and Fig. 5k). Pathways upregulated in small B cells included BCR signaling, histone modification and T cell exhaustion, whereas *CTLA4* signaling in cytotoxic T lymphocytes was downregulated (Fig. 5l). In large B cells, p53 and apoptosis signaling were upregulated, although *PTEN* was downregulated, suggesting activation of intrinsic stress responses, whereas canonical proliferative and epigenetic programs such as mTOR, ERK/MAPK, PI3K signaling, and DNA methylation and transcriptional repression were downregulated. This reflects the hypomethylation typically observed in Richter’s transformation versus CLL and de novo DLBCL and indicates a shift from canonical proliferative circuits. Additionally, suppression of T cell antigen receptor signaling may reflect impaired immune engagement, and upregulation of extracellular matrix remodeling suggests altered tumor–stroma interactions (Extended Data Fig. 9f). Aberrant cell-surface expression of co-inhibitory receptors CTLA4 and LAG-3 on large B cells may play a role in immune escape (Fig. 5m). Spatial scoring of T cell activation and exhaustion (based on previously published gene lists^38–40^) highlighted DLBCL hotspots, while higher scores of BCR activation were observed in the CLL region, suggesting reduced BCR survival signaling dependency in the transformed lymphoma (Supplementary Fig. 9a,b and Fig. 5n),

### Investigating the role of miRNAs in Richter’s transformation

**Extended Data Fig. 10:**
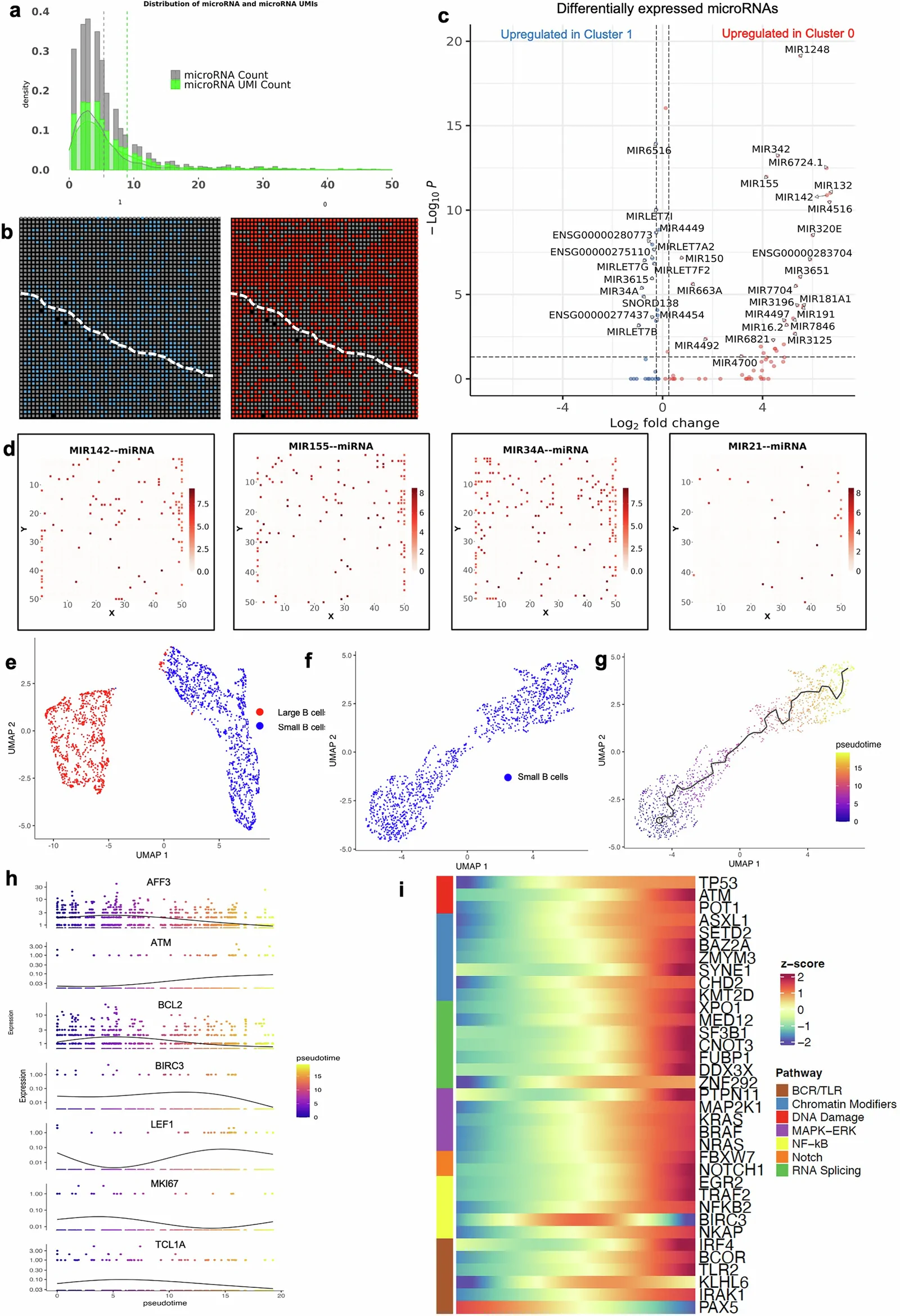
Analysis of microRNAs, transcriptional pathways and pathway dynamics in Richter’s Transformation. **a** Distribution of microRNAs and corresponding UMI counts per spatial pixel. Dashed lines indicate the average number of microRNAs or UMI counts. **b** Spatial clustering of clusters 0 and 1 whose spatial distributions mirrored CLL and DLBCL regions from histological staining of Richter’s transformation sample. **c** Differentially expressed microRNAs between clusters 0 and 1. Differential gene expression computed from two-sided Wilcoxon rank-sum test, adjusted P value on the basis of Bonferroni correction) **d** Spatial mapping of select microRNAs implicated in lymphoid malignancies. **e**–**g**, Monocle3 UMAP visualization of large B cells and small B cells e showing distinct partitions in transcriptomic space f Monocle3 UMAP visualization of small B cells g Monocle3 pseudotime UMAP highlighting a dynamic trajectory. **h** Pseudotime heat map expression dynamics of genes associated with transformation, including anti-apoptotic regulators (*BCL2, BIRC3*), proliferation (*MKI67*), and key signaling nodes (*ATM*, *LEF1, TCL1A*). **i** Heat map of pathway-associated gene expression (z-score) across pseudotime reveals coordinated activation of DNA damage response, chromatin modifiers, and NF-κB and MAPK-ERK signaling, consistent with progressive transformation.

DBiTplus further enables spatial profiling of small noncoding RNAs such as microRNAs (miRNAs), which regulate mRNA synthesis and gene expression^41^. The miR-17-92 cluster, miR-150 and miR-15b are critical for B cell differentiation and germinal center selection, whereas miR-21, miR-155 and miR-222 are known to be upregulated in lymphoid malignancies, with high miR-21 expression linked to the activated B cell subtype of DLBCL^42^. Unsupervised clustering at the DBiTplus spot level revealed seven distinct miRNA clusters, with clusters 0 and 1 corresponding to the histological transition from CLL to DLBCL (Extended Data Fig. 10a,b). Differential analysis identified several miRNAs relevant to disease progression: miR-34a, miR-21 and miR-155 (Extended Data Fig. 10c). Interestingly, miR-21 is known to inhibit expression of *PTEN* and activate the PI3K–AKT pathway, increasing chemotherapy resistance^43^, confirming the small and large B cell pathway analysis (Extended Data Fig. 10d). Other miRNAs such as miR-342 and miR-132, implicated in other lymphomas, warrant further investigation in the context of Richter’s transformation. These findings demonstrate the unique capability of DBiTplus to spatially map miRNAs and interrogate their roles in hematological malignancies.

### Single-cell pseudotime dissects the molecular evolution of CLL-transformed B cells

We applied Monocle 3 to the DBiTplus-deconvoluted single-cell transcriptomes of this CLL-DLBCL transformation. UMAP visualization revealed distinct clusters for large B cells and small B cells, indicating substantial transformation-associated transcriptional shifts (Extended Data Fig. 10e). Pseudotime analysis of small B cells revealed dynamic gene expression changes: *ATM* (linked to NF-κB activation), *MKI67* (proliferation) and *LEF1* (diagnostic CLL marker) increased over pseudotime, whereas *AFF3, BCL2* and *TCL1A* decreased (Extended Data Fig. 10f–h). Pseudotime heat map highlighted progressive upregulation of several CLL-associated genes across multiple pathways such as DNA damage response (*TP53*, *ATM*), chromatin modification (*ASXL1*, *SETD2*) and RNA splicing (*SF3B1*; Extended Data Fig. 10i). *BIRC3*, a tumor suppressor and negative regulator of noncanonical NF-κB signaling in CLL, exhibited transient upregulation before declining, consistent with 11q deletion-associated poor prognosis. These transcriptional dynamics reveal gradual remodeling of the CLL transcriptome toward a more aggressive state, underscoring the molecular changes that may underlie Richter’s transformation.

## Discussion

Spatial multi-omics integrates genomics, transcriptomics, proteomics and metabolomics while preserving spatial context, providing a comprehensive view of molecular processes in tissues. Traditional approaches utilize separate assays on adjacent tissue sections limiting alignment and multimodal integration accuracy due to tissue heterogeneity. To overcome this, we developed DBiTplus, which combines unbiased transcriptome-wide spatial sequencing with mxIF (CODEX or CellScape) on the same tissue section, compatible with both OCT-frozen and FFPE samples. We optimized enzymatic cDNA retrieval using RNase H, while preserving tissue architecture for multiplexed imaging.

By registering DBiTplus data with high-resolution imaging, we achieve precise colocalization of transcriptomic and proteomic data. This enabled mxIF-informed DBiTplus spot deconvolution, allowing for accurate identification of cell types. Our approach involved splitting spots into pure cell-type sub-spots and utilizing the Seurat WNN methodology for reliable cell-type deconvolution and splitting the transcriptomes of individual sub-spots into single-cell transcriptional profiles. Thus, leveraging mxIF data to guide the splitting of DBiT spatial transcriptomes can enable the creation of truly single-cell-level spatially resolved transcriptome atlases.

Application to human lymph node and lymphoma samples revealed distinct spatial organization and enabled high-resolution mapping of lymphoma progression and transformation. DBiTplus also captures the whole milieu of small RNAs, particularly miRNAs, providing insights into the molecular biological mechanism of disease evolution.

DBiTplus shares common limitations with sequencing-based spatial transcriptomics approaches; low capture depth can lead to dropout of low-abundance transcripts, which may be exacerbated by the lower transcript recovery rate of DBiTplus, compared to the standard DBiT-seq workflow, an important consideration when profiling rare cell populations. Further, the tight clamping of the tissue during the DBiTplus workflow may disrupt tissue architecture outside the clamped region and may lead to reduced imaging signal. Nonetheless, these limitations are counterbalanced by several strengths: DBiTplus enables whole-transcriptome spatial profiling without being constrained by predesigned panels, can be integrated with high-plex protein imaging on the same tissue section, and is flexible and cost-effective for broad adoption. While Xenium and CosMx achieve single-cell spatial resolution through direct imaging of transcripts, DBiTplus even allows for unbiased profiling of total RNAs including small noncoding RNAs (that is, miRNAs), which is unique for discovery of new RNA biological mechanisms.

In summary, DBiTplus represents a spatial multi-omics approach that integrates sequencing-based and imaging-based spatial assays on the same tissue section, enabling image-guided deconvolution into single-cell-resolved spatial transcriptomes. By combining multiple molecular layers at single-cell resolution, DBiTplus provides unprecedented insights into tissue architecture and cellular interactions, opening new avenues for spatial multi-omics.

## Methods

### Human specimens

De-identified archived benign FFPE human lymph node and lymphoma tissue blocks were obtained from Yale Pathology Tissue Services (YPTS). The tissue retrieval and distribution for research was conducted with the approval of the Yale University Institutional Review Board (approved IRB no. 1401013259) and oversight by the Tissue Resource Oversight Committee. Written informed consent for participation in any cases where identification was collected alongside the specimen, was obtained from individuals or their guardians, in accordance with the principles of the Declaration of Helsinki. Each sample was handled in strict compliance with HIPAA regulations, University Research Policies, Pathology Department diagnostic requirements and Hospital bylaws.

### Mouse tissue slides

The mouse tissue used in this study was obtained from a commercial vendor, Zyagen (San Diego, CA), which procured and handled the animals under their in-house Institutional Animal Care and Use Committee (IACUC)-approved protocols. Because no live animal procedures were conducted at our institution, separate IACUC approval was not required. C57 embryo sagittal frozen sections, E13 (MF-104-13-C57), C57 spleen frozen sections (MF-701-C57) and C57 embryo sagittal paraffin sections, E11 (MP-104-11-C57), were purchased from Zyagen. Mouse embryo frozen sagittal sections and mouse C57 spleen frozen sections were made of freshly collected tissues, snap frozen in OCT blocks. E11 mouse whole-embryo paraffin sagittal sections were made of freshly collected tissues, fixed in 10% neutral buffered formalin, and processed for paraffin embedding. Both OCT and paraffin blocks were also sectioned at a thickness of 7–10 μm and mounted on the center of poly-L-lysine-covered glass slides (63478-AS, Electron Microscopy Sciences).

### Human tissue section preparation

After review and selection by a board-certified pathologist, optimal paraffin blocks were sectioned by YPTS at a thickness of 7–10 μm and mounted on the center of poly-L-lysine-coated 1 × 3-inch glass slides. Serial tissue sections were collected simultaneously for DBiT-seq and H&E staining. Human brain cerebellum paraffin sections (HP-202) were purchased from Zyagen and made of freshly collected tissues, fixed in 10% neutral buffered formalin and processed for paraffin embedding. Paraffin sections were stored at −80 °C until use.

### RNA quality assessment

To perform RNA integrity number tests, 15–20-μm-thick curls were obtained from YPTS. The RNeasy FFPE Kit for RNA Extraction from Qiagen was used. Following RNA extraction, the High Sensitivity RNA ScreenTape assay was used with the Agilent TapeStation system to assess RNA quality. The RNA integrity number equivalent, which automatically assesses RNA degradation, and the DV_200_, the percentage of RNA fragments > 200 nucleotides, were used as metrics for determining RNA quality.

### Fabrication of microfluidic device

Details of the fabrication process for the PDMS wafers and microfluidic chips can be found in a prior publication^3^.

### DNA barcode annealing

The DNA oligonucleotides were obtained from Integrated DNA Technologies, with the sequences provided in Supplementary Tables 6 and 7. The barcodes (100 μM) and ligation linker (100 μM) were annealed at a 1:1 ratio in 2× annealing buffer (20 mM Tris-HCl pH 8.0, 100 mM NaCl, 2 mM EDTA). The mixes were placed in a thermal cycle and heated to 97 °C to anneal and slowly cooled to room temperature at a rate of −0.1 °C s^−1^. The barcodes can be stored at −20 °C for up to 6 months.

### DBiTplus profiling of fresh frozen tissues

OCT-embedded tissue sections stored in a −80 °C freezer were allowed to equilibrate to room temperature. The section was then fixed with 4% formaldehyde for 20 min and washed three times with 0.5× DPBS-RI (1× DPBS diluted with nuclease-free water and 0.05 U μl^−1^ RNase Inhibitor). The tissue was permeabilized for 20 min at room temperature using 0.5% Triton X-100 in DPBS, followed by a wash with 0.5× DPBS-RI (1× DPBS diluted with nuclease-free water and 0.05 U μl^−1^ RNase Inhibitor) to stop the permeabilization. After air-drying, a PDMS reservoir was placed over the region of interest (ROI) on the tissue slide. In situ polyadenylation was performed with *Escherichia coli* poly(A) polymerase. The samples were first equilibrated by adding 100 μl wash buffer (88 μl nuclease-free water, 10 μl 10× Poly(A) Reaction Buffer, 2 μl 40 U μl^−1^ RNase Inhibitor) and incubating at room temperature for 5 min. After removing the wash buffer, 60 μl of the Poly(A) enzymatic mix (38.4 μl nuclease-free water, 6 μl 10× Poly(A) Reaction Buffer, 6 μl 5 U μl^−1^ Poly(A) Polymerase, 6 μl 10 mM ATP, 2.4 μl 20 U μl^−1^ SUPERase•In RNase Inhibitor, 1.2 μl 40 U μl^−1^ RNase Inhibitor) was added to the reaction chamber and incubated in a humidified box at 37 °C for 30 min. To remove excess reagents, the slide was dipped in 50 ml DPBS and shake-washed for 5 min. Next, 60 μl of the reverse transcription mix (20 μl 25 μM RT Primer, 16.3 μl 0.5× DPBS-RI, 12 μl 5× RT Buffer, 6 μl 200 U μl^−1^ Maxima H Minus Reverse Transcriptase, 4.5 μl 10 mM dNTPs, 0.8 μl 20 U μl^−1^ SUPERase•In RNase Inhibitor, 0.4 μl 40 U μl^−1^ RNase Inhibitor) was loaded into the PDMS reservoir and sealed with parafilm. The sample was incubated at room temperature for 30 min and then at 42 °C for 90 min, followed by a wash with 50 ml DPBS. For the in situ ligation of barcode A, the first PDMS device was carefully aligned over the tissue slide, positioning the 50 center channels over the ROI. The chip was imaged to record positions for downstream alignment and analysis. An acrylic clamp was used to secure the PDMS to the slide, preventing interchannel leakage. The ligation mix (100 μl 1× NEBuffer 3.1, 61.3 μl nuclease-free water, 26 μl 10× T4 ligase buffer, 15 μl T4 DNA ligase, 5 μl 5% Triton X-100, 2 μl 40 U μl^−1^ RNase Inhibitor and 0.7 μl 20 U μl^−1^ SUPERase•In RNase Inhibitor) was prepared. For the barcoding reaction, 5 μl of a ligation solution (comprising 4 μl of ligation mix and 1 μl of 25 μM DNA barcode A (A1-A50)) was added to each of the 50 inlets. The solution was drawn through the channels using a gently controlled vacuum. After incubating at 37 °C for 30 min, the PDMS chip was removed, and the slide was washed with 50 ml of DPBS. Next, a second PDMS device with 50 perpendicular channels was attached to the air-dried slide over the ROI. A brightfield image was taken, and barcode B was ligated similarly. The tissue section was then washed with nuclease-free water to remove any residual salts, and a final brightfield image was taken to mark the boundaries of the microfluidic channels on the tissue ROI.

### Tissue deparaffinization and decrosslinking

FFPE tissue sections were allowed to equilibrate to room temperature from the −80 °C freezer. Tissue sections were baked for 1 h at 60 °C to facilitate the removal of paraffin and increase adhesion of the tissue section to the slide. The tissue slide was then immersed into xylene twice for 5 min for deparaffinization. This was followed by rehydration steps in a series of ethanol dilutions: two rounds of 100% ethanol, and one each of 90%, 70%, 50% and 30% ethanol. Finally, the tissue sections were immersed in distilled water for 5 min. Next, the tissue slide was immersed into preheated antigen retrieval buffer (Discovery CC1 buffer (Roche, Basel) or Tris-EDTA Buffer, pH 9.0 (Abcam)) and allowed to boil at 95–100 °C for 10 min and then allowed to cool to room temperature. The intact tissue slide was then imaged using the ×10 objective on the M7000 Imaging EVOS System and the same profiling approach described above applied.

### cDNA retrieval post-spatial barcoding

For cDNA retrieval, the barcoded region of the tissue was covered with a clean PDMS well gasket, and 100 μl cDNA extraction solution (10 μl 5% Triton X-100, 74 μl nuclease-free water, 10 μl 1× RNase H Reaction Buffer and 6 μl Thermostable RNase H (M0523S, New England Biolabs)) was loaded into it. The reservoir was then clamped tightly with the slide to avoid any leakage and sealed with parafilm. The clamped tissue slide was incubated in a humidified box at 55 °C for 3 h. Following this, the cDNA extraction solution was collected and 1 μl of 0.5 M EDTA was added to inactivate the RNase H enzyme. The intact tissue slide was washed with 100 μl of nuclease-free water, which was then collected. Following this, 100 μl of cDNA extraction solution was added to the tissue slide as described previously, and the clamped slide was incubated in a humidified box at 37 °C overnight. The cDNA extraction solution was collected and 1 μl of 0.5 M EDTA was added to inactivate the RNase H enzyme. The intact tissue slide was washed with 100 μl of nuclease-free water, which was also collected and an additional wash step with 0.1× SSC buffer done. The collected cDNA extraction solution was then pooled and stored at −80 °C until use. For control slides, the standard lysing process described in previous publications from the lab was followed^3^.

### DAPI staining

The tissue clamps were removed, and the intact tissue was washed in nuclease-free water and then with 1× PBS. Following this, the tissue slide was incubated with 500 μl of DAPI solution (two drops of NucBlue Fixed Cell ReadyProbes Reagent in 500 μl of 1× PBS) and incubated at room temperature for 5 min. The tissue slide was then imaged in the DAPI channel using the ×20 objective on the EVOS M7000 Imaging System. This image is used to co-register the DBiTplus and mxIF images. The tissue slide was then washed with 1× PBS three times and stored at −80 °C until the multiplexed fluorescence imaging step.

### cDNA purification, template switch and PCR amplification

To construct the sequencing library, the pooled cDNA extraction solution was first purified using the Zymo DNA Clean & Concentrator-5 kit, following the recommended 5:1 ratio and eluted into 100 μl of nuclease-free water. Biotinylated cDNAs were captured using streptavidin beads (Dynabeads MyOne Streptavidin C1, Invitrogen). Before use, the beads were washed three times with 1× B&W buffer containing 0.05% Tween 20 and resuspended in 100 µl of 2× B&W buffer (10 mM Tris-HCl pH 7.5, 1 mM EDTA, 2 M NaCl). The beads were then mixed with the purified cDNA in a 1:1 volume ratio and incubated with gentle rotation at room temperature for 60 min. The beads were subsequently washed twice with 1× B&W buffer and once with 1× Tris buffer containing 0.1% Tween 20. Streptavidin beads bound with cDNA molecules were resuspended in 200 μl of TSO Mix (75 μl nuclease-free water, 40 μl 5× RT buffer, 40 μl 20% Ficoll PM-400, 20 μl 10 mM dNTPs, 10 μl 200 U μl^−1^ Maxima H Minus Reverse Transcriptase, 5 μl 40 U μl^−1^ RNase Inhibitor, 10 μl 100 μM TSO Primer). The template switch reaction was performed at room temperature for 30 min and then at 42 °C for 90 min with gentle rotation. Afterward, the beads underwent a single wash with 10 mM Tris-HCl pH 7.5 containing 0.1% Tween 20 and another wash with nuclease-free water. Second-strand synthesis was then performed as follows: the beads were washed twice with TE-TW buffer (10 mM Tris pH 8, 1 mM EDTA, 0.01% Tween 20) and resuspended in freshly prepared 200 μl 0.1 M NaOH for 5 min with gentle rotation. The beads were washed once with 500 μl of TE-TW, and once with 500 μl 1× TE buffer (10 mM Tris pH 8, 1 mM EDTA). The beads were then resuspended in 200 μl second-strand synthesis reaction solution (40 μl Maxima 5× RT Buffer, 20 μl 10 mM dNTPs, 2 μl 1 mM dN-SMRT oligo, 5 μl Klenow Enzyme 133 μl H_2_O) and rotated end-over-end at 37 °C for 1 h. The beads were washed with nuclease-free water and were resuspended in 200 μl of PCR Mix (100 μl 2× KAPA HiFi HotStart ReadyMix, 84 μl nuclease-free water, 8 μl 10 μM PCR Primer 1, 8 μl 10 μM PCR Primer 2). This suspension was then distributed into PCR strip tubes. An initial amplification was performed with the following PCR program: 95 °C for 3 min, five cycles at 98 °C for 20 s, 63 °C for 45 seconds, 72 °C for 3 min, followed by an extension at 72 °C for 3 min and a hold at 4 °C. The PCR product was purified using SPRIselect beads at a 0.8× ratio, according to the manufacturer’s standard protocol. The resulting cDNA amplicon was then analyzed using the TapeStation system with D5000 DNA ScreenTape and reagents. The purified PCR product can be stored at −20 °C until the next steps if necessary.

### rRNA removal, library preparation and sequencing

The SEQuoia RiboDepletion Kit was used to remove rRNA and mitochondrial rRNA from the amplified cDNA, following the manufacturer’s instructions. 20 ng of cDNA was used as the input amount, and three rounds of depletion were performed. We observed that two rounds of depletion could suffice. Next, following the PCR steps described in the previous step, ten PCR cycles were sufficient to directly ligate sequencing primers, using a 100 μl system consisting of 50 μl 2× KAPA HiFi HotStart ReadyMix, ~27 μl solution from the rRNA removal step, 4 μl 10 μM P5 primer, and 4 μl 10 μM P7 primer and ~15 μl of water. The library product was purified using SPRIselect beads at a 0.7× ratio, according to the manufacturer’s standard protocol, and sent out to Novogene Corporation to be sequenced on an Illumina NovaSeq 6000 or NovaSeq X Plus Sequencing System with paired-end reads of 150 base pairs in length.

### CODEX spatial multiplexing using PhenoCycler-Fusion

A modified version of the CODEX PhenoCycler-Fusion protocol (https://www.akoyabio.com/wp-content/uploads/2021/01/CODEX-User-Manual.pdf) was adopted for tissue sections used in the DBiTplus workflow. Since the tissue had already been deparaffinized and rehydrated during the DBiTplus workflow, the CODEX process began with a gentle antigen retrieval step using 1× AR9 buffer for 5–10 min. The tissue was then allowed to cool to room temperature and was rinsed twice with nuclease-free water and hydration buffer, followed by staining buffer as the antibody cocktail was prepared. The tissue slide was incubated with the antibody cocktail at room temperature for 3 h in a humidified chamber. After incubation, the tissue underwent a series of steps including post-fixation, ice-cold methanol incubation and a final fixation step. Attached to the flow cell, the tissue section was incubated in 1× PhenoCycler buffer with additive for at least 10 min to improve adhesion. The CODEX cycles were then set up, the reporter plate was prepared and loaded, and the imaging process began. A final .qptiff file was generated, at the end that could be viewed using QuPath (V0.5.1)^44^. For further details on the PhenoCycler antibody panels, experimental cycle design and reporter plate volumes, see Supplementary Data 1.

### CellScape mxIF staining and imaging

MxIF staining and imaging was performed using the CellScape platform (Bruker Spatial Biology). Human FFPE CLL samples were prepared as previously described and shipped to Bruker Spatial Biology in Saint Louis at 4 °C. Pre-DBIT samples were shipped in standard slide mailers. Post-DBIT samples were shipped in 50 ml conical tubes with PBS to prevent sample dehydration in transit. In preparation for staining, pre-DBIT samples were deparaffinized and rehydrated according to the CellScape user manual. Briefly, pre-DBIT samples were washed for 5 min three times in HistoClear II (Electron Microscopy Sciences, Hatfield), two times in 100% ethanol (EtOH), one time each in 90% EtOH, 70% EtOH, 50% EtOH, 30% EtOH and, finally, CellScape Wash Buffer. Immediately following deparaffinization (pre-DBIT samples), or 24 h before running the CellScape experiment (post-DBIT), heat-induced epitope retrieval was performed at 110–120 °C under low pressure in a pressure cooker with samples placed in plastic Coplin jars (Fisher, Waltham) filled with Discovery CC1 buffer (Roche, Basel) for 15 min. Thereafter, samples were allowed to cool for 25 min on the benchtop. Slides were then washed with CellScape Wash Buffer, mounted in the CellScape Whole-Slide Imaging Chamber, which was immediately filled with CellScape Storage Buffer and stored at 4 °C until use. The multiplex proteomic assay was performed on the CellScape platform using automated iterative cycles of fluorophore-conjugated primary antibody staining, imaging and photobleaching. The assay consisted of 12 cycles, with each cycle beginning with a 10-s photobleach and subsequent background measurement for each channel to be stained. This was followed by automated staining of up to three antibodies incubated for 15 min per cycle. Antibodies were subsequently washed off and fluorescence images were acquired. The 31-plex assay included Sytox Green (Thermo, S7020), the VistaPlex Cell Boundaries (Bruker Spatial Biology, VISTAPLEX3101), Immune Profiling (Bruker Spatial Biology, VISTAPLEX3102) and Architecture (Bruker Spatial Biology, VISTAPLEX 3103) kits, as well as the following custom-conjugated primary antibodies: LEF1 (Abcam, ab137872), CD5 (Leica, CD5-4C7-L-U) and CD23 (Leica, CD23-1B12-L-U).

### Flow cell removal and H&E staining

Following the mxIF imaging, the flow cell can be removed and histological H&E staining performed on the same tissue section. To remove the flow cell, the tissue slide with the flow cell is immersed in xylene or HistoClear for a minimum of 20 min to weaken the adhesive. A razor was then used to carefully detach the flow cell from the tissue slide. The tissue slide was then rinsed thoroughly with deionized water and then with 1× PhenoCycler Buffer without additive three times for 10 min each. Histological H&E staining on the FFPE sections was conducted by YPTS. The H&E images were taken using the Motic EasyScan digital slide scanner at a magnification of ×40.

### Assessing quality of mxIF imaging data after DBiTplus

The standard CODEX data preprocessing and filtering pipeline was first applied to extract cell positions and cell-level features from two adjacent CODEX slices separately. Six marker points were then manually identified from the two stacked CODEX images. The optimal affine transformation, which minimized the distance between paired marker points, was estimated using the ‘transform’ module from the Python package ‘skimage’. This transformation was subsequently applied to align cell positions from the two slices into a common coordinate system. To ensure statistical robustness, the spatial dimensions were discretized into bins with a pixel size of 100 (~4,000 common bins), and the mean values of selected features were computed by aggregating the cells within each bin. Common bins across both datasets were identified, and Pearson correlation coefficients were calculated to quantify the strength of linear relationships between corresponding features, and scatterplots were generated to visually represent the correlations.

### Sequence alignment and generation of gene expression matrix

Read 2 from the FASTQ file was processed to extract UMIs and spatial barcodes A and B. Read 1, containing cDNA sequences, was aligned to the mouse GRCm39 or human GRCh38 reference genome using STAR (V2.7.8a)^45^. Spatial barcode sequences were demultiplexed with ST_Pipeline (V1.8.1)^46^, using the predefined coordinates of the microfluidic channels, and ENSEMBL IDs were converted to gene names. This generated a gene-by-spot expression matrix for downstream analysis. Entries in the matrix that corresponded to spot positions without tissue were excluded.

### Read mapping of noncoding RNA species

The spatial transcriptomic data from the Richter’s transformation (Fig. 5) was preprocessed following the ASTRO Pipeline^47^, also utilized by Bai et al.^13^.

### Downsampling for gene and UMI comparison across samples

For comparative analyses, and to account for varying sequencing depths, the raw sequencing reads were uniformly downsampled to the read count of the sample with the minimum number of reads using Seqkit (V2.3.1)^48^. The downsampled reads were processed as described in the sequence alignment and generation of gene expression matrix section above. The average number of UMIs and genes per pixel were calculated during the spatial gene expression analysis using the Seurat (V4.3.0)^49^ pipeline, taking into account only useful pixels (pixels actually covering tissue) and were visualized as violin plots.

### Spot-level gene data normalization and unsupervised clustering analysis

Spatial gene expression analysis at the spot level was conducted using the Seurat V4.3.0 pipeline. Initially, gene expression within each spot was normalized and variance-stabilized using the SCTransform method, specifically designed for scRNA-seq datasets. Linear dimensional reduction was then performed with the ‘RunPCA’ function, and the optimal number of principal components for further analysis was determined using a heuristic approach, visualized by an ‘Elbow plot’ that ranks principal component analysis (PCA) components by their variance percentages. Subsequently, the ‘FindNeighbors’ function embedded spots into a *k*-nearest-neighbor graph structure based on Euclidean distance in PCA space, and the ‘FindClusters’ function applied a modularity optimization technique to cluster the spots. The ‘RunUMAP’ function was used to visually explore spatial heterogeneities through the UMAP algorithm. Finally, DEGs defining each cluster were identified using the ‘FindMarkers’ function for pairwise comparisons between spot groups or ‘FindAllMarkers’ for DEGs for each identity (cluster or cell type) versus all other cells combined.

### scRNA-seq reference data

All scRNA-seq data utilized in this study are publicly available. The scRNA-seq dataset for the mouse embryo was obtained from the Mouse Organogenesis Cell Atlas Project, which can be accessed at https://oncoscape.v3.sttrcancer.org/atlas.gs.washington.edu.mouse.rna/landing/. For this study, cells with a ‘development_stage’ of 10.5 to 11.5 days post-coitum were selected to correspond with CODEX measurements, using the MaxFuse method as described below. Doublet removal and cell annotations were conducted by the original authors of the dataset. The scRNA-seq dataset for human lymph nodes was retrieved from the Cell2location repository, and is available for download at https://urldefense.com/v3/__https:/cell2location.cog.sanger.ac.uk/paper/integrated_lymphoid_organ_scrna/RegressionNBV4Torch_57covariates_73260cells_10237genes/sc.h5ad__;!!IBzWLUs!XDud8EZcXLnWNMGYjYHAFdgJyM_lWL-_j9l6RweZDqQXLP1uY3a78B7_-BgAEzSHFVLk6TfuCwekjLtKGhyb4z8FJVvCtJhUcRVGqg$/. Cell-type annotations were provided by the original study.

### Preprocessing of CODEX/CellScape (mxIF) imaging data

Whole-cell segmentation was performed using the Mesmer method^14^ using pretrained weights. For cell mask prediction, the default training resolution of 0.5 μm per spot was adopted. Cells within the [0.05, 0.95] cell-size quantile range and possessing DAPI signal intensities exceeding the 0.1 quantile threshold were retained for analysis. mxIF features were extracted by summing the signal for each feature per cell. For each mxIF feature, the 0.05 and 0.95 quantiles were calculated, and each single-cell-level mxIF feature was subsequently scaled to a [0, 1] range, with the 0.05 quantile mapped to 0 and the 0.95 quantile mapped to 1. Values exceeding this range were clipped to 0 or 1, as appropriate.

### Integration of mxIF imaging data with scRNA-seq

The integration of scRNA-seq data and mxIF data was accomplished utilizing the MaxFuse algorithm^11^. Before integration, standard preprocessing protocols were applied to all scRNA-seq data using Scanpy. This preprocessing included count normalization, log1p transformation and the identification of highly variable genes, resulting in the selection of 5,000 genes exhibiting the highest variability. Linked features between the scRNA-seq and mxIF datasets were identified based on corresponding protein and gene names. From these linked features, those with a standard deviation greater than 0.01 were selected to enhance integration performance. During the pivot matching process, the number of principal components used to construct the nearest-neighbor graph was determined by examining the elbow of the singular value decomposition plot. A smoothing weight of 0.3 was applied, as recommended by the MaxFuse method, to account for the weak linkage between the two modalities. Following integration, low-quality pivots were removed to ensure the reliability of the cross-modal pivot pairs. Approximately 10% of cells from the mxIF datasets, representing high-confidence matches, were selected to construct these pivot pairs. For each pivot pair, cell-type labels from the scRNA-seq data were transferred to the matched mxIF cells. To extend cell-type annotation to the entire mxIF dataset, a support vector machine model was trained on the pivot mxIF cells to predict cell-type labels based on protein expression measurements. Once trained, the model was applied to the remaining non-pivot cells within the mxIF dataset, thereby achieving comprehensive cell-type annotation across the dataset.

### Large B cell annotation

To isolate the highly proliferative, enlarged B cell population in the MZL and CLL to DLBCL samples, we applied a three-way thresholding strategy based on protein abundance and morphometric size. For each of the markers CD20 and Ki67, as well as for the per-cell area measurement, we calculated the 60th-percentile value across all cells in the tissue section. Any cell whose CD20 intensity exceeded the CD20 0.6 quantile and whose Ki67 intensity exceeded the Ki67 0.6 quantile and whose physical cell size exceeded the area 0.6 quantile simultaneously was labeled as a ‘large B cell’. Cells failing to meet this simultaneous triple threshold were not assigned to this category. All cells that did not satisfy the ‘large B’ criteria were subsequently merged with the scRNA-seq reference using the MaxFuse pipeline, followed by the label-transfer procedure detailed in the ‘Integration of mxIF imaging data with scRNA-seq’ section. Conversion of cell size from pixel to area was based on the resolution of 0.5 μm per pixel. Diameter was inferred by modeling the cell as a circle and computing the diameter based on number of pixels in the segmentation mask.

### mxIF-DBIT alignment

To align the high-resolution mxIF image with the low-resolution DBiTplus spot image of the FFPE mouse embryo dataset, tissue boundaries were initially identified in both images. The input image was first smoothed using a Gaussian filter (implemented via the ‘filters.gaussian’ function from the scikit-image package) to reduce noise and enhance relevant structures. Subsequently, Otsu’s thresholding method (used through the ‘filters.threshold_otsu’ function from the scikit-image package) was applied to the smoothed image to compute an optimal threshold value. This threshold was then used to generate a binary image, effectively separating the foreground (potential tissue) from the background. To ensure the detected regions were solid, any holes within the binary regions were filled using a binary hole-filling algorithm (utilizing the ‘binary_fill_holes’ function from the SciPy package). The final output was a binary image with no holes enabling identification of the tissue’s outer boundary. Following the identification of tissue boundaries, an optimal similarity transformation was determined and applied to the mxIF image. This transformation was computed using the ‘transform’ module from the scikit-image package, aiming to minimize the squared error loss between the transformed mxIF image and the DBiTplus image. Given the cell positions in the mxIF image and spot positions in the DBiTplus image, the learned image transformation can be utilized to register the cells. This registration ensures that cellular-level data from the mxIF images are correctly aligned with the spatial transcriptomics data from the DBiTplus spots.

### DBiTplus spot cell-type deconvolution and splitting

The reference scRNA-seq dataset was normalized to a common total count per cell corresponding to the dataset’s median sequencing depth. Specifically, total counts were calculated for each cell in the scRNA-seq dataset, the median value was determined and this value was used as the ‘target_sum‘ in the function ‘scanpy.pp.normalize_total’. Following normalization, the average expression profile for each cell type was computed based on the normalized counts. *μ*_*k*,*j*_ denotes the average raw expression of gene *j* of cell type *k* in the scRNA-seq data. The deconvolution of DBiTplus spots is achieved by computing the cell-type proportions of mxIF cells aligned to each spot. Let *β*_*k*,*i*_ denote the proportion of the contribution of cell type *k* to spot *i*, and *x*_*i*,*j*_ denote the total raw expression of gene *j* in DBiTplus spot *i*. To attribute gene expression to specific cell types within each DBiTplus spot, we split each spot into pure cell-type sub-spots. This is done by computing the expected cell-type-specific gene expression at each DBiTplus spot. The calculation uses the formula shown in equation (1):

1 $${\hat{x}}_{i,\,j,k}=\frac{{x}_{i,\,j}\times {{\beta }_{k,i}\times \mu }_{k,\,j}}{{\sum }_{{k}^{{{{\prime} }}}=1}^{K}{\beta }_{{k}^{{{{\prime} }}}\!,i}\times {\mu }_{{k}^\prime\!,\,j}}$$

### Single-cell-level gene data normalization and unsupervised clustering analysis

For single-cell-level gene data analysis, to normalize gene expression values, we first calculated the median UMI counts per pure cell-type sub-spot. This median value was then used as the scale.factor in the NormalizeData function so that all sub-spots are normalized to a common library size representative of the dataset’s central tendency. Linear dimension reduction was then performed with the ‘RunPCA’ function, and the optimal number of principal components for further analysis was determined using a heuristic approach, visualized by an ‘elbow plot’ that ranks PCA components by their variance percentages. Subsequently, the ‘FindNeighbors’ function embedded spots into a *k*-nearest-neighbor graph structure based on Euclidean distance in PCA space, and the ‘FindClusters’ function (resolution = 0.7, all other parameters at default values) applied a modularity optimization technique to cluster the sub-spots. The ‘RunUMAP’ function was used to visually explore spatial heterogeneities through the UMAP algorithm. Finally, DEGs defining each cluster were identified using the ‘FindMarkers’ function for pairwise comparisons between spot groups or ‘FindAllMarkers’ for differential expression for each identity (cluster or cell type) versus all other cells combined.

### Seurat WNN analysis

mxIF cells were aggregated into pure cell-type sub-spots, and DBiTplus spots were similarly divided into pure cell-type sub-spots. Subsequent analyses were performed using the Seurat WNN methodology, with measurements from the pure cell-type sub-spots serving as input data. The RNA data underwent normalization using a scale factor derived from the median UMI count. Following normalization, variable features were identified, and PCA was conducted on the RNA assay. Subsequently, the default assay was switched to the protein modality, variable features were identified, and PCA was performed on the protein data under the reduction name ‘apca’. For each cell, the nearest neighbors within the dataset were determined based on a weighted combination of RNA and protein similarities. The cell-specific modality weights and multimodal neighbors were calculated using the ‘FindMultiModalNeighbors’ function. The integrated results were then utilized for visualization and clustering.

### Deconvolution comparisons with other methods

We used Cell2location for the deconvolution analysis. First, a negative binomial regression model was trained to estimate reference transcriptomic profiles for all cell types profiled with scRNA-seq data (sc_subsampled_park2020.h5ad). The ‘major celltype’ annotation was used to ensure consistency. Lowly expressed genes were excluded using the filtering strategy recommended by Cell2location (cell_count_cutoff = 5, cell_percentage_cutoff2 = 0.03, nonz_mean_cutoff = 1.12). The model was trained for 600 epochs and reached convergence. Next, we estimated cell-type abundances in the spatial transcriptomics dataset (LN50UM) using the reference profiles. The following Cell2location hyperparameters were applied: N_cells_per_location = 33 (estimated from nuclei counts in H&E images using QuPath, version 0.50). Training was stopped after 50,000 iterations, with all other parameters set to default values. To assess concordance between our mxIF-guided deconvolution and the Cell2location results, we calculated, for each spot, the Pearson correlation between the vectors of cell-type proportions produced by the two methods. Correlations were summarized by: (i) the proportion of spots above thresholds (>0.9, >0.8, >0.7, >0.6), and (ii) spatial heat maps of correlation values derived from spot coordinates. We also plotted the overall correlation distribution using 0.1-wide bins. RCTD provides a principled, likelihood-based framework for inferring which single-cell transcriptional programs underlie the mRNA captured in each spatial spot. Conceptually, it treats the observed counts as a mixture of reference cell-type profiles and estimates the mixture fractions that best explain the data, while explicitly accounting for technical variability such as total UMI depth. In our workflow, the analysis was implemented in R (spacexr v2.2.1) with Seurat, SeuratDisk and zellkonverter facilitating seamless interchange between Seurat and AnnData formats. The DBiTplus dataset supplied the query layer: raw spot-level counts were extracted from the Spatial assay, two-dimensional spot coordinates were obtained through GetTissueCoordinates() and spot-specific library sizes were computed with colSums(counts). These three components were wrapped in a SpatialRNA() object, which together with a preprocessed single-cell reference served as input to create.RCTD(). Model fitting proceeded via run.RCTD() with doublet_mode = ‘full’. The resulting cell-type weight matrix was normalized so that proportions across all reference types sum to one in every spot (normalize_weights()), appended to the DBiT Seurat object with AddMetaData(), and finally exported for downstream comparison with other deconvolution methods and for integration into our spatial analyses. Cell-type deconvolution with TACCO (v0.2.2) was performed in Python. Deconvolution was carried out with tc.tl.annotate, which treats every spatial spot as a compositional mixture of the single-cell expression profiles. Specifically, we passed the DBiT AnnData object (adata) and the gene-matched reference (ref) to tc.tl.annotate, using ‘celltype’ as the label to be transferred, result_key = ‘TACCO’ to store the output, and reconstruction_key = ‘rec’ to save reconstructed expression values. Setting multi_center = 1 let the algorithm place a single, data-driven centroid in expression space for each cell type, striking a balance between flexibility and over-fitting. After inference, ‘tc.pp.filter’ was applied to mirror any gene or cell filtering decisions across reference and query, ensuring that subsequent comparisons rested on an identical feature space.

### Pseudotime analysis

Pseudotemporal analysis was performed with Monocle 3 (ref. ^15^) on average gene expressions of pure cell-type sub-spots obtained from proteome-informed cell-type deconvolution of the DBiTplus spots. The Seurat object was subset to include only cell types of interest. The raw count matrix was converted converted to a monocle cell dataset using the ‘new_cell_data_set’ function. The raw count matrix was normalized with the ‘preprocess_cds’ function. Dimensionality reduction was performed with the ‘reduce_dimension’ function with default parameters (reduction_method = ‘UMAP’, umap.n_neighbors = 15, umap.min_dist = 0.1, preprocess_method = ‘PCA’). Next, the cells were clustered using the ‘cluster_cell’ function and the ‘learn_graph’ function to generate a principal graph from the reduced dimension space. The ‘order_cells’ function was used to order the observations in a pseudotemporal manner and the trajectory visualized with the ‘plot_cells’ function. Pseudotime plots of specific genes were plotted using the ‘plot_genes_in_pseudotime’ function. The ‘graph_test’ function was used to identify DEGs and the heat map of the DEGs plotted (both *k*-means and hierarchical clustering) with a modified version of code from ref. ^50^.

### Ingenuity pathway analysis

Ingenuity pathway analysis (Qiagen)^51^ was used to explore the biological pathways implicated in the DEGs from our Seurat clusters or cell types. The gene expression profile of each pure cell-type sub-spot was replicated *m* times where *m* is the number of cells of the corresponding cell type in the corresponding spot. This replication step ensured proper sample sizes for *P*-value calculations. The DEGs per cluster or cell type are generated using the FindAllMarkers function or FindMarkers (for pairwise comparisons) in Seurat. The list of genes with corresponding log_2_fold change values, *P* value and adjusted *P* value of each gene, was exported as a CSV file and used as input for the Qiagen ingenuity pathway analysis software. The Ingenuity Knowledge Base (genes only) served as the reference set for performing core expression analysis. The *z*-score assesses the activation or inhibition level of specific pathways by measuring the congruence between the observed gene expression profile and the pathway’s known regulatory pattern from the literature. Positive *z*-scores denote predicted activation or upregulation, whereas negative *z*-scores indicate inhibition or downregulation. A *z*-score ≥ 2 signifies a statistically significant upregulation in pathway activity whereas a *z*-score ≤ −2 denotes significant downregulation of a specific pathway. The significance (−log_10_*P* value) of each pathway enrichment is further evaluated using a right-tailed Fisher’s exact test. Pathways were plotted as bar charts using Prism V10 or R (4.2.0-foss-2020b). The graphical summary provides a quick overview of the major biological themes in the ingenuity pathway analysis core analysis. The upstream regulator analysis identifies a list of upstream regulators that may be responsible for the observed gene expression changes in the list of DEGs in our datasets. The enhanced causal network analysis provides a comprehensive approach to identifying upstream molecules that control the expression of the DEGs in our datasets.

### Statistical analysis

Prism V10 (GraphPad) was used for statistical analyses and the specific tests used are indicated in the main text and figure captions.

### Reporting summary

Further information on research design is available in the Nature Portfolio Reporting Summary linked to this article.

## Online content

Any methods, additional references, Nature Portfolio reporting summaries, source data, extended data, supplementary information, acknowledgements, peer review information; details of author contributions and competing interests; and statements of data and code availability are available at https://doi.org/10.1038/s41592-025-02948-0.

## Supplementary information

Supplementary Information Supplementary Figs. 1–9 and Tables 1–8.

Reporting Summary

Supplementary Data 1 Details of antibody source, amount/dilution, supplier name, catalog number, clone name and imaging cycle details.

## Data Availability

Raw and processed data reported are deposited in the Gene Expression Omnibus under accession GSE308167. The resulting fastq files were aligned to the mouse reference genome (GRCm39) or human reference genome (GRCh38). mxIF imaging datasets and histological images are available at https://doi.org/10.5281/zenodo.17153112 (ref. ^52^) and https://zenodo.org/records/17153187 (ref. ^53^). Published data for data comparison are available online at STOmicsDB MOSTA (E13.5_E1S2.MOSTA.h5ad; https://db.cngb.org/stomics/mosta/spatial/) and Allen Mouse Brain Atlas (Developing Mouse Brain—age, E13.5; Theiler stage, TS21; https://developingmouse.brain-map.org/). Patho-DBiT reference lymph node data are available on the Gene Expression Omnibus under accession number GSE274641 (sample GSM8454083).

## Code availability

The code for the analyzing the processed mxIF imaging datasets and the sequencing datasets are available on Zenodo (https://doi.org/10.5281/zenodo.16827046)^54^ and GitHub (https://github.com/Janezjz/DBiT-plus/).

## References

[1] Baysoy, A., Bai, Z., Satija, R. & Fan, R. The technological landscape and applications of single-cell multi-omics. *Nat. Rev. Mol. Cell Biol.* **24**, 695–713 (2023). 37280296 10.1038/s41580-023-00615-w PMC10242609

[2] Rodriques, S. G. et al. Slide-seq: a scalable technology for measuring genome-wide expression at high spatial resolution. *Science* **363**, 1463–1467 (2019). 30923225 10.1126/science.aaw1219 PMC6927209

[3] Liu, Y. et al. High-spatial-resolution multi-omics sequencing via deterministic barcoding in tissue. *Cell* **183**, 1665–1681.e18 (2020). 33188776 10.1016/j.cell.2020.10.026 PMC7736559

[4] Liu, Y. et al. High-plex protein and whole transcriptome co-mapping at cellular resolution with spatial CITE-seq. *Nat. Biotechnol.* **41**, 1405–1409 (2023). 36823353 10.1038/s41587-023-01676-0 PMC10567548

[5] Farzad, N. et al. Spatially resolved epigenome sequencing via Tn5 transposition and deterministic DNA barcoding in tissue. *Nat. Protoc.*10.1038/s41596-024-01013-y (2024).38943021

[6] Xia, C., Fan, J., Emanuel, G., Hao, J. & Zhuang, X. Spatial transcriptome profiling by MERFISH reveals subcellular RNA compartmentalization and cell cycle-dependent gene expression. *Proc. Natl Acad. Sci. USA* **116**, 19490–19499 (2019). 31501331 10.1073/pnas.1912459116 PMC6765259

[7] Wang, X. et al. Three-dimensional intact-tissue sequencing of single-cell transcriptional states. *Science*10.1126/science.aat5691 (2018). PMC6339868 29930089

[8] Black, S. et al. CODEX multiplexed tissue imaging with DNA-conjugated antibodies. *Nat. Protoc.* **16**, 3802–3835 (2021). 34215862 10.1038/s41596-021-00556-8 PMC8647621

[9] Li, Y. et al. All-optical multimodal mapping of single cell-type–specific metabolic activities via REDCAT. Preprint at *bioRxiv* 10.1101/2024.11.07.622511 (2024). PMC13541623 42660991

[10] Yeo, Y. Y. et al. Same-slide spatial multi-omics integration reveals tumor virus-linked spatial reorganization of the tumor microenvironment. Preprint at *bioRxiv* 10.1101/2024.12.20.629650 (2024).

[11] Chen, S. et al. Integration of spatial and single-cell data across modalities with weakly linked features. *Nat. Biotechnol.* **42**, 1096–1106 (2024). 37679544 10.1038/s41587-023-01935-0 PMC11638971

[12] Cable, D. M. et al. Robust decomposition of cell type mixtures in spatial transcriptomics. *Nat. Biotechnol.* **40**, 517–526 (2022). 33603203 10.1038/s41587-021-00830-w PMC8606190

[13] Bai, Z. et al. Spatially exploring RNA biology in archival formalin-fixed paraffin-embedded tissues. *Cell*10.1016/j.cell.2024.09.001 (2024). 39353436 PMC11568911

[14] Greenwald, N. F. et al. Whole-cell segmentation of tissue images with human-level performance using large-scale data annotation and deep learning. *Nat. Biotechnol.* **40**, 555–565 (2022). 34795433 10.1038/s41587-021-01094-0 PMC9010346

[15] Cao, J. et al. The single-cell transcriptional landscape of mammalian organogenesis. *Nature* **566**, 496–502 (2019). 30787437 10.1038/s41586-019-0969-x PMC6434952

[16] Chen, A. et al. Spatiotemporal transcriptomic atlas of mouse organogenesis using DNA nanoball-patterned arrays. *Cell* **185**, 1777–1792.e21 (2022). 35512705 10.1016/j.cell.2022.04.003

[17] Mages, S. et al. TACCO unifies annotation transfer and decomposition of cell identities for single-cell and spatial omics. *Nat. Biotechnol.* **41**, 1465–1473 (2023). 36797494 10.1038/s41587-023-01657-3 PMC10513360

[18] Kleshchevnikov, V. et al. Cell2location maps fine-grained cell types in spatial transcriptomics. *Nat. Biotechnol.* **40**, 661–671 (2022). 35027729 10.1038/s41587-021-01139-4

[19] Liu, Y., Beyer, A. & Aebersold, R. On the dependency of cellular protein levels on mRNA abundance. *Cell* **165**, 535–550 (2016). 27104977 10.1016/j.cell.2016.03.014

[20] Li, J., Zhang, Y., Yang, C. & Rong, R. Discrepant mRNA and protein expression in immune cells. *Curr. Genomics* **21**, 560–563 (2020). 33414677 10.2174/1389202921999200716103758 PMC7770634

[21] Zhang, D. et al. Inferring super-resolution tissue architecture by integrating spatial transcriptomics with histology. *Nat. Biotechnol.* 10.1038/s41587-023-02019-9 (2024). 38168986 PMC11260191

[22] Arribas, A. J. et al. Resistance to PI3Kδ inhibitors in marginal zone lymphoma can be reverted by targeting the IL-6/PDGFRA axis. *Haematologica* **107**, 2685–2697 (2022). 35484662 10.3324/haematol.2021.279957 PMC9614536

[23] Bret, C., Klein, B. & Moreaux, J. Gene expression-based risk score in diffuse large B-cell lymphoma. *Oncotarget* **3**, 1700–1710 (2012). 23482333 10.18632/oncotarget.807 PMC3681505

[24] Cai, C. et al. Anthrax toxin receptor 1/tumor endothelial marker 8 promotes gastric cancer progression through activation of the PI3K/AKT/mTOR signaling pathway. *Cancer Sci.* **111**, 1132–1145 (2020). 31977138 10.1111/cas.14326 PMC7156833

[25] Ferreres, J. R. et al. PRRX1 silencing is required for metastatic outgrowth in melanoma and is an independent prognostic of reduced survival in patients. *Mol. Oncol.* **18**, 2471–2494 (2024). 38978350 10.1002/1878-0261.13688 PMC11459042

[26] Flumann, R. et al. Distinct genetically determined origins of Myd88/BCL2-driven aggressive lymphoma rationalize targeted therapeutic intervention strategies. *Blood Cancer Discov.* **4**, 78–97 (2023). 36346827 10.1158/2643-3230.BCD-22-0007 PMC9816818

[27] Liu, Y. et al. Palmitoylation by ZDHHC family members regulate B-cell lymphoma growth. *Int. J. Biol. Macromol.* **320**, 145876 (2025). 40645243 10.1016/j.ijbiomac.2025.145876

[28] Zhang, W. et al. Exosome complex genes mediate RNA degradation and predict survival in mantle cell lymphoma. *Oncol. Lett.* **18**, 5119–5128 (2019). 31612023 10.3892/ol.2019.10850 PMC6781731

[29] Rivas, M. A. et al. Cohesin core complex gene dosage contributes to germinal center derived lymphoma phenotypes and outcomes. *Front. Immunol.* **12**, 688493 (2021). 34621263 10.3389/fimmu.2021.688493 PMC8490713

[30] Puvvada, S. D. et al. Pharmacogenetic approaches identify ANTXR1 as a potential mediator of response to therapeutic NF-κB inhibition in diffuse large B cell lymphoma. *Blood* **118**, 234 (2011).

[31] Broséus, J. et al. Molecular characterization of Richter syndrome identifies de novo diffuse large B-cell lymphomas with poor prognosis. *Nat. Commun.* 10.1038/s41467-022-34642-6 (2023) PMC9852595 36658118

[32] Parry, E. M. et al. Evolutionary history of transformation from chronic lymphocytic leukemia to Richter syndrome. *Nat. Med.* **29**, 158–169 (2023). 36624313 10.1038/s41591-022-02113-6 PMC10155825

[33] Parikh, S. A. et al. Diffuse large B-cell lymphoma (Richter syndrome) in patients with chronic lymphocytic leukaemia (CLL): a cohort study of newly diagnosed patients. *Br. J. Haematol.* **162**, 774–782 (2013). 23841899 10.1111/bjh.12458 PMC4098845

[34] Kipps, T. J. et al. Chronic lymphocytic leukaemia. *Nat. Rev. Dis. Primers* **3**, 16096 (2017). 28102226 10.1038/nrdp.2016.96 PMC5336551

[35] Sun, C. et al. The immune microenvironment shapes transcriptional and genetic heterogeneity in chronic lymphocytic leukemia. *Blood Adv.* **7**, 145–158 (2023). 35358998 10.1182/bloodadvances.2021006941 PMC9811214

[36] Mahmoud, A. M., Gaidano, G. & Mouhssine, S. Immunological aspects of Richter syndrome: from immune dysfunction to immunotherapy. *Cancers* **15**, 1015 (2023). 36831361 10.3390/cancers15041015 PMC9954516

[37] Mouhssine, S. & Gaidano, G. Richter syndrome: from molecular pathogenesis to druggable targets. *Cancers* **14**, 4644 (2022). 36230566 10.3390/cancers14194644 PMC9563287

[38] Zhang, C. et al. Prioritizing exhausted T cell marker genes highlights immune subtypes in pan-cancer. *iScience* **26**, 106484 (2023). 37091230 10.1016/j.isci.2023.106484 PMC10119613

[39] Rouillard, A. D. et al. The harmonizome: a collection of processed datasets gathered to serve and mine knowledge about genes and proteins. *Database* **2016**, baw100 (2016). 27374120 10.1093/database/baw100 PMC4930834

[40] Diamant et al. Harmonizome 3.0: integrated knowledge about genes and proteins from diverse multi-omics resources. *Nucleic Acids Res.* **53**, D1016–D1028 (2025). 39565209 10.1093/nar/gkae1080 PMC11701526

[41] Ademokun, A. & Turner, M. Regulation of B-cell differentiation by microRNAs and RNA-binding proteins. *Biochem. Soc. Trans.* **36**, 1191–1193 (2008). 19021522 10.1042/BST0361191

[42] Malpeli, G. et al. MYC-related microRNAs signatures in non-Hodgkin B-cell lymphomas and their relationships with core cellular pathways. *Oncotarget* **9**, 29753–29771 (2018). 30038718 10.18632/oncotarget.25707 PMC6049865

[43] Lawrie, C. H. et al. Microrna expression distinguishes between germinal center B cell-like and activated B cell-like subtypes of diffuse large B cell lymphoma. *Int. J. Cancer* **121**, 1156–1161 (2007). 17487835 10.1002/ijc.22800

[44] Bankhead, P. et al. QuPath: open source software for digital pathology image analysis. *Sci. Rep.* **7**, 16878 (2017). 29203879 10.1038/s41598-017-17204-5 PMC5715110

[45] Dobin, A. et al. STAR: ultrafast universal RNA-seq aligner. *Bioinformatics* **29**, 15–21 (2013). 23104886 10.1093/bioinformatics/bts635 PMC3530905

[46] Navarro, J. F., Sjostrand, J., Salmen, F., Lundeberg, J. & Stahl, P. L. ST Pipeline: an automated pipeline for spatial mapping of unique transcripts. *Bioinformatics* **33**, 2591–2593 (2017). 28398467 10.1093/bioinformatics/btx211

[47] Zhang, D. et al. ASTRO: automated spatial whole-transcriptome RNA-expression workflow. Preprint at *bioRxiv* 10.1101/2025.01.24.634814 (2025).

[48] Shen, W., Le, S., Li, Y. & Hu, F. SeqKit: a cross-platform and ultrafast toolkit for FASTA/Q file manipulation. *PLoS ONE* **11**, e0163962 (2016). 27706213 10.1371/journal.pone.0163962 PMC5051824

[49] Hao, Y. et al. Integrated analysis of multimodal single-cell data. *Cell* **184**, 3573–3587.e29 (2021). 34062119 10.1016/j.cell.2021.04.048 PMC8238499

[50] Tambalo, M., Mitter, R. & Wilkinson, D. G. A single cell transcriptome atlas of the developing zebrafish hindbrain. *Development* **147**, dev184143 (2020). 32094115 10.1242/dev.184143 PMC7097387

[51] Kramer, A., Green, J., Pollard, J. Jr & Tugendreich, S. Causal analysis approaches in ingenuity pathway analysis. *Bioinformatics* **30**, 523–530 (2014). 24336805 10.1093/bioinformatics/btt703 PMC3928520

[52] Enninful, A. Integration of imaging-based and sequencing-based spatial omics mapping on the same tissue section via DBiTplus-multiplexed IF images. *Zenodo*10.5281/zenodo.17153112 (2025). PMC13541625 41540123

[53] Enninful, A. Integration of imaging-based and sequencing-based spatial omics mapping on the same tissue section via DBiTplus-multiplexed IF and H&E images. *Zenodo*10.5281/zenodo.17153187 (2025). PMC13541625 41540123

[54] Enninful, A. DBiTplus_updated. *Zenodo*10.5281/zenodo.16827046 (2025).

