# Supplementary material for: Integration of imaging-based and sequencing-based spatial omics mapping on the same tissue section via DBiTplus
Source: Nat Methods. 2026 Jan 15;23(9):1827–39. doi: 10.1038/s41592-025-02948-0 (PMC13541625; doi:10.1038/s41592-025-02948-0)
Supplement: Supplementary file 1 [file 41592_2025_2948_MOESM1_ESM.pdf]

nature methods

Supplementary information https://doi.org/10.1038/s41592-025-02948-0

Integration of imaging-based and sequencing-based spatial omics mapping on the same tissue section via DBiTplus

In the format provided by the authors and unedited

Supplementary Information

Integration of Imaging-based and Sequencing-based Spatial Omics Mapping on the Same Tissue Section via DBiTplus

Archibald Enninful[1#], Zhaojun Zhang[2#], Dmytro Klymyshyn[3], Matthew Ingalls[4], Mingyu Yang[1], Hailing Zong[3], Zhiliang Bai[1], Negin Farzad[1], Graham Su[1], Alev Baysoy[1], Jungmin Nam[1], Yao Lu[1], Shuozhen Bao[1], Siyan Deng[1], Nancy R. Zhang[2], Oliver Braubach[4], Mina L. Xu[5*], Zongming Ma[6*], Rong Fan[1,5, 7,8*]

[1]Department of Biomedical Engineering, Yale University, New Haven, CT, 06520, USA.
[2]Department of Statistics and Data Science, The Wharton School, University of Pennsylvania, Philadelphia, PA, USA.
[3]Akoya Biosciences, Inc. 1080 O'Brien Dr Suite A, Menlo Park, CA 94025 USA.
[4]Bruker Spatial Biology, 4340 Duncan Avenue, St. Louis, MO, 63110, USA.
[5]Department of Pathology, Yale School of Medicine, New Haven, CT, 06520, USA.
[6]Department of Statistics and Data Science, Yale University, New Haven, CT, 06520, USA.
[7]Yale Stem Cell Center and Yale Cancer Center, Yale School of Medicine, New Haven, CT, 06520, USA.
[8]Human and Translational Immunology Program, Yale School of Medicine, New Haven, CT, 06520, USA.

# These authors contributed equally to this work
* Corresponding authors: M.X. mina.xu@yale.edu; Z.M, zongming.ma@yale.edu; R.F. rong.fan@yale.edu

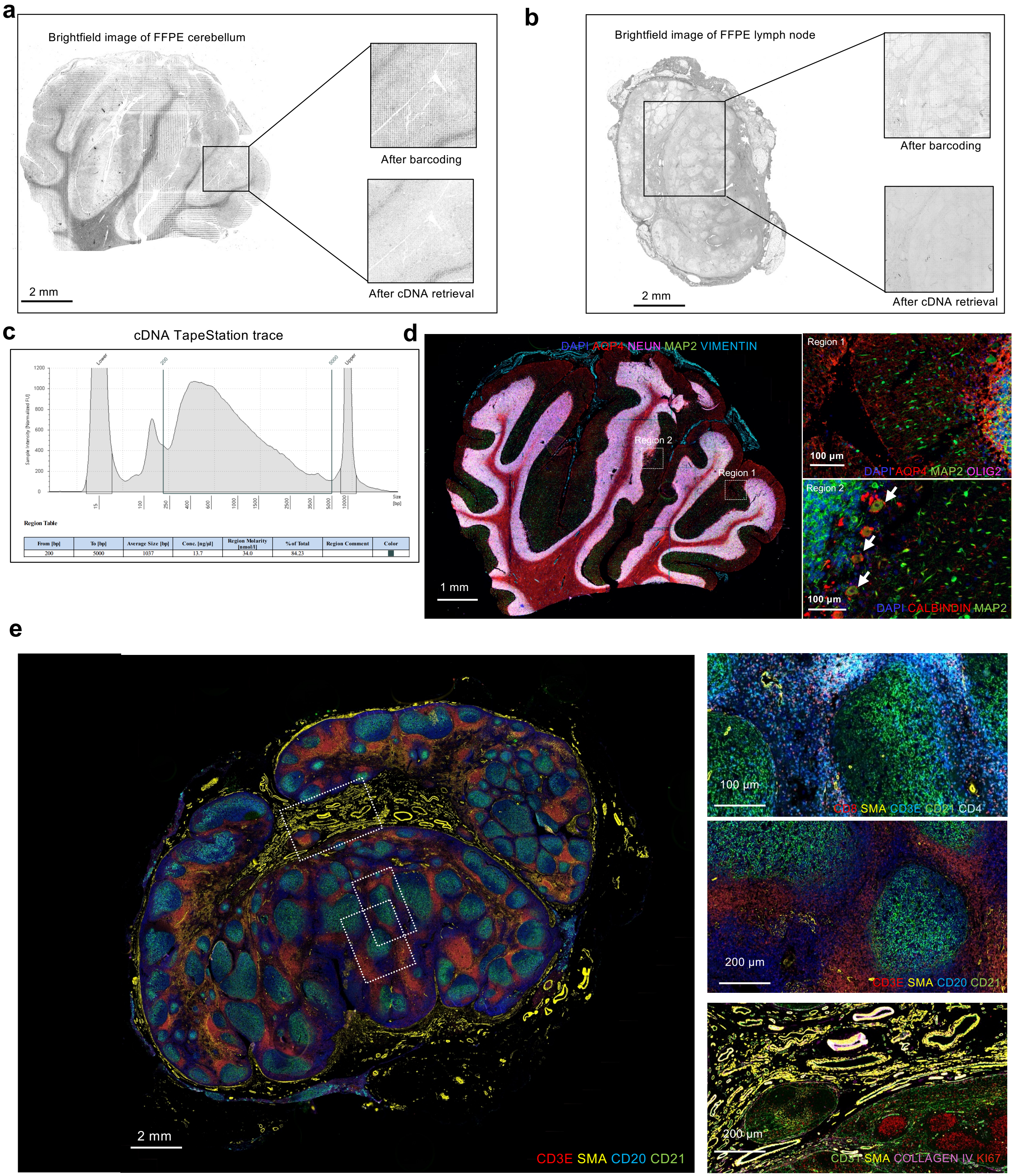
a
Brightfield image of FFPE cerebellum
After barcoding
After cDNA retrieval
2 mm
b
Brightfield image of FFPE lymph node
After barcoding
After cDNA retrieval
2 mm
c
cDNA TapeStation trace
Region Table
From [bp]
To [bp]
Average Size [bp]
Conc. [ng/µl]
Region Molarity [nmol/l]
% of Total
Region Comment
Color
200
5000
1037
13.7
34.0
84.23
d
DAPI AQP4 NEUN MAP2 VIMENTIN
Region 2
Region 1
1 mm
100 µm
DAPI AQP4 MAP2 OLIG2
DAPI CALBINDIN MAP2
e
2 mm
CD3E SMA CD20 CD21
100 µm
CD8 SMA CD3E CD21 CD4
200 µm
CD3E SMA CD20 CD21
200 µm
CD31 SMA COLLAGEN IV KI67

Supplementary Fig. 1. Optimization of CODEX imaging on FFPE human cerebellum and lymph node following RNaseH-based cDNA retrieval in the DBiTplus workflow. a Brightfield image of FFPE human cerebellum following barcoding in the DBiTplus workflow. Box represents the barcoded region. Brightfield image after cDNA retrieval shows the tissue morphology is preserved and is suitable for CODEX imaging. **b** Brightfield image of FFPE of human lymph node. Box represents the barcoded region. Brightfield image after cDNA retrieval shows the tissue morphology is preserved and is suitable for CODEX imaging. **c** Size distribution from TapeStation traces of cDNA amplicon. **d** CODEX imaging of FFPE human cerebellum following DBiTplus (from panel a) (n = 1) Left: CODEX image showing AQP4 (astrocytes), MAP2 (synaptic neurons), NeuN (neuronal nuclei and cell bodies) and Vimentin (vasculature). Right top: Synaptic neurons marked by MAP2 in the molecular layer of the cerebellum. Right bottom: Large pear-shaped Purkinje cells, with their large cell bodies visualized in the Purkinje cell layer. Three distinct regions are further analyzed for quality of CODEX staining. **e** CODEX imaging of FFPE human lymph node following DBiTplus (n = 1). Left: overview of the entire tissue section with markers CD3E, CD20, CD21, and SMA. Right top: Region showing T cell subtypes: CD3ε (pan T cells), CD8 (cytotoxic T cells), CD4 (helper T cells) and SMA (smooth muscles). Middle: CD3ε (pan T cells), SMA (smooth muscle), CD20 (B cells) and CD21 (follicular dendritic cells). Bottom: SMA (smooth muscle), Ki67 (proliferating germinal center B cells), Collagen IV (blood vessels), CD31 (vasculature).

a

FINE-TUNED CELL TYPES	COARSE CELL TYPES	FINE-TUNED CELL TYPES	COARSE CELL TYPES
Jaw and tooth progenitors	Mesodermal cells	Cardiac muscle lineages	Mesodermal cells
Lens	Schwann cell precursor	Cholinergic neurons	Neural cells
Limb mesenchyme	Mesodermal cells	Chondroctye progenitors	Mesodermal cells
Megakaryocytes	Blood cells	Chondrocytes & osteoblasts	Mesodermal cells
Melanocytes	Mesodermal cells	Connective tissue progenitors	Mesodermal cells
Myocytes	Mesodermal cells	Definitive erythroid lineage	Blood cells
Neural Tube	Neural cells	Early mesenchyme	Mesodermal cells
Neural progenitor cells	Neural cells	Endothelial cells	Endothelial cells
Notochord cells	Notochord cells	Ependymal cell	Neural cells
Oligodendrocyte Progenitors	Neural cells	Epithelial cells	Epithelial cells
Osteoblasts	Mesodermal cells	Excitatory neurons	Neural cells
Postmitotic premature neurons	Neural cells	Granule neurons	Neural cells
Premature oligodendrocyte	Neural cells	Hepatocytes	Endodermal cells
Primitive erythroid lineage	Blood cells	Inhibitory interneurons	Neural cells
Radial glia	Neural cells	Inhibitory neuron progenitors	Neural cells
Schwann cell precursor	Schwann cell precursor	Inhibitory neurons	Neural cells
Sensory neurons	Neural cells	Intermediate Mesoderm	Mesodermal cells
Stromal cells	Stromal cells	Isthmic organizer cells	Neural cells
White blood cells	Blood cells		

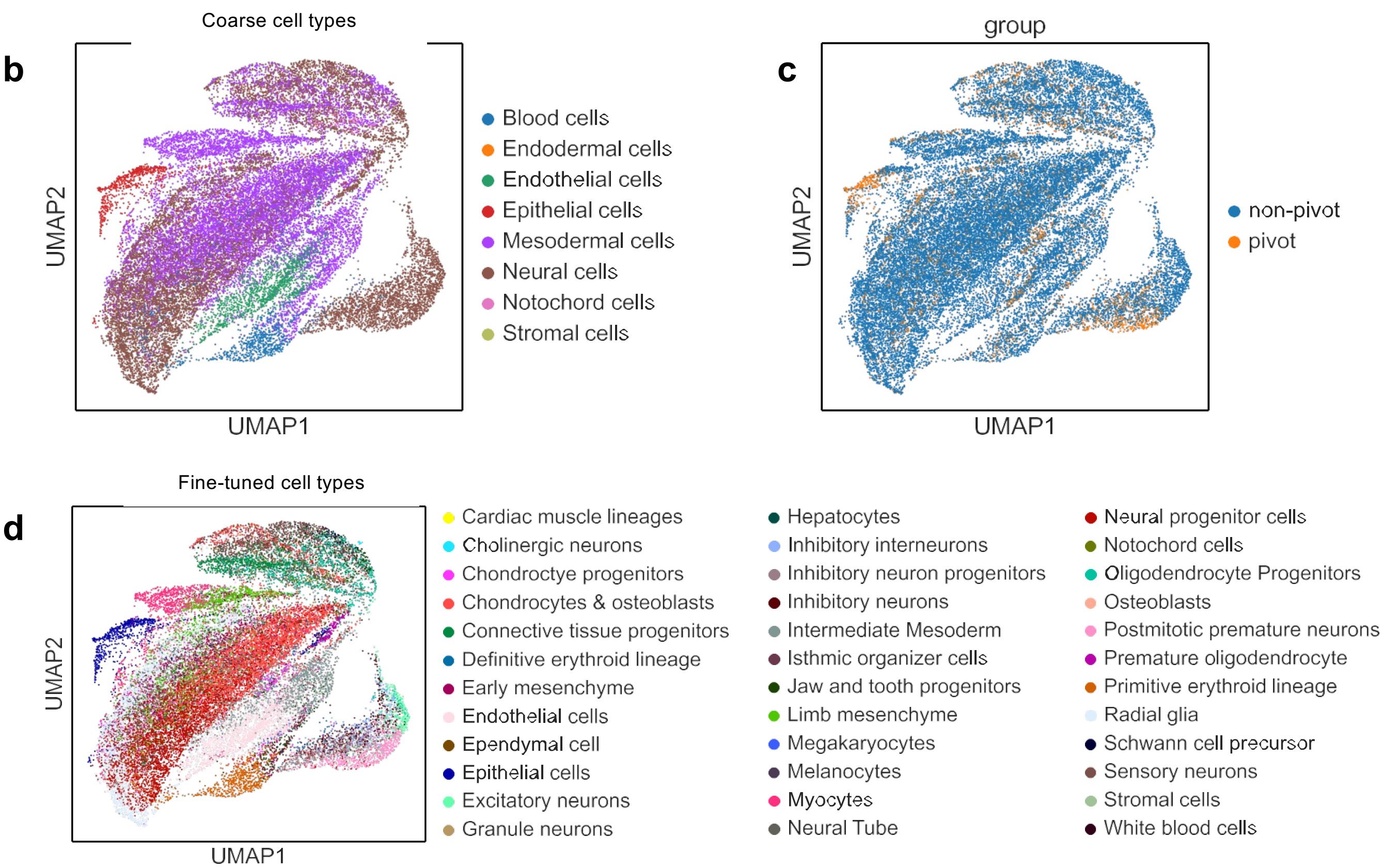

Supplementary Fig. 2. Integration of CODEX spatial proteomic data with single-cell RNA-seq reference using MaxFuse. **a** Table mapping fine-tuned and coarse cell type annotations from the single-cell RNA-seq reference dataset of E11 FFPE mouse embryo. **b** UMAP visualization of the integrated dataset showing coarse cell types. **c** UMAP plot showing the distribution of pivot and non-pivot cells identified during MaxFuse integration. Pivot cells are used as anchors for label transfer between the two modalities. **d** UMAP visualization of fine-tuned cell types within the integrated dataset.

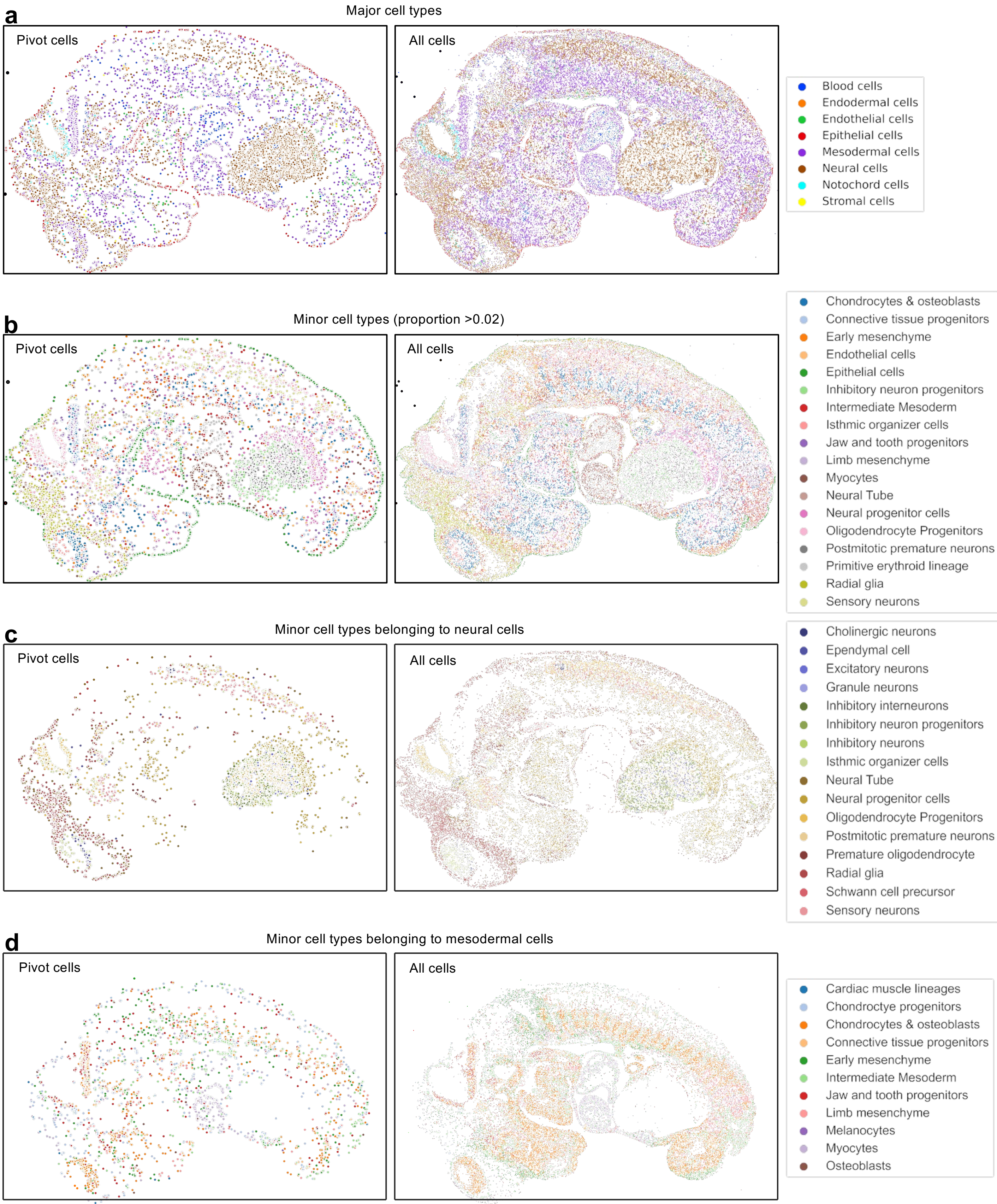

Supplementary Fig. 3. Spatial mapping of pivot and non-pivot cell types from integrated CODEX and MOCA RNA-seq reference dataset using MaxFuse. a Spatial plot of pivot cells and all cells from the matched single cell RNA-seq and CODEX datasets for major cell types. **b** Spatial plot of pivot cells and all cells from the matched single cell RNA-seq and CODEX datasets for minor cell types. **c** Spatial plot of pivot cells and all cells from the matched single cell RNA-seq and CODEX datasets for minor cell types belonging to neural cells. **d** Spatial plot of pivot cells and all cells from the matched single cell RNA-seq and CODEX datasets for minor cell types belonging to mesodermal cells.

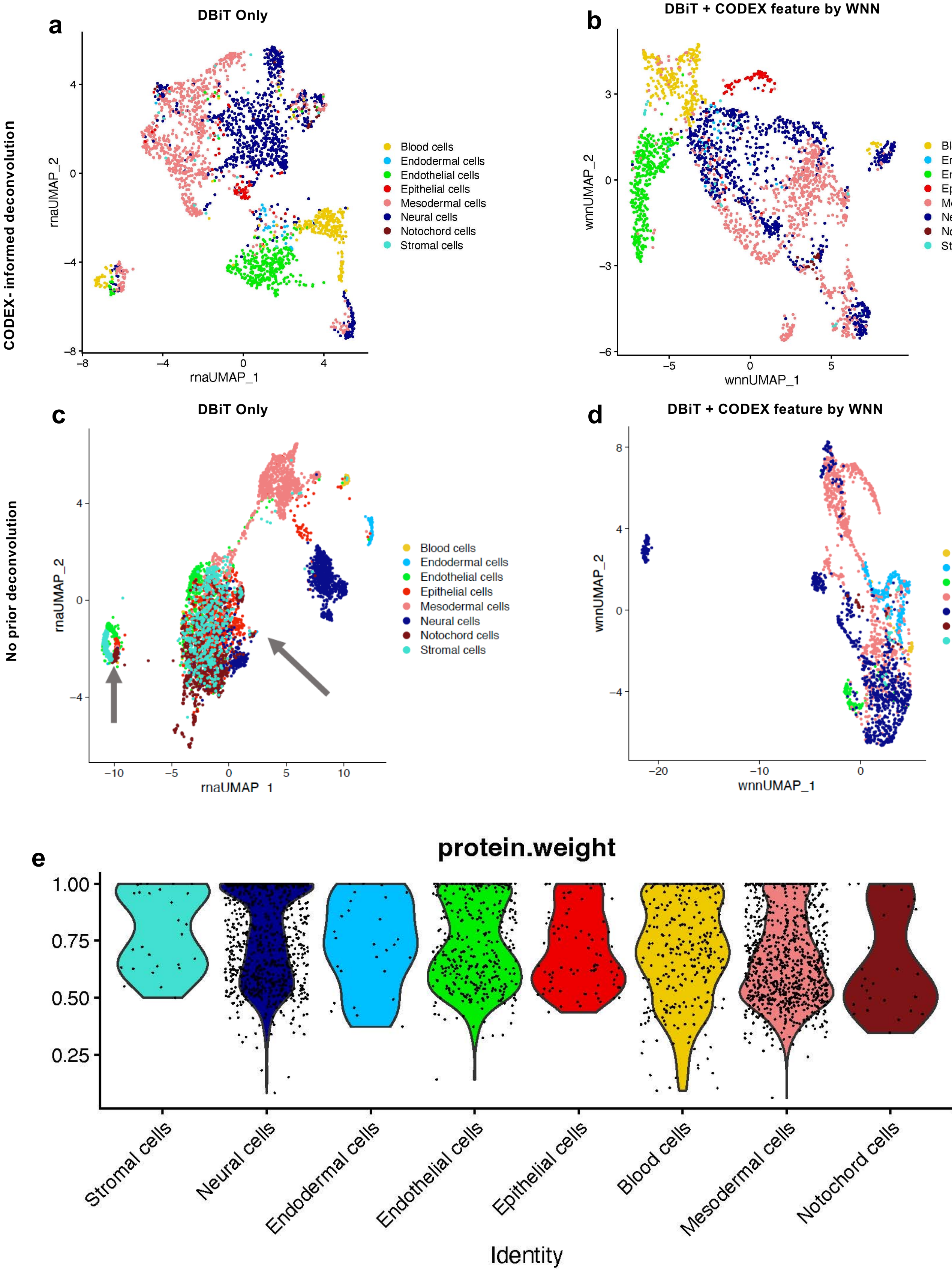

Supplementary Fig. 4. Deconvolution and WNN integration of DBiTplus datasets. a UMAP showing CODEX-informed deconvolution of DBiTplus feature only **b.** UMAP showing a combination of DBiTplus and CODEX features **c.** UMAP showing no prior deconvolution with DBiTplus feature only **d.** UMAP showing DBiTplus and CODEX feature. **e** Violin plot of Seurat WNN integration of DBiTplus and CODEX datasets showing the distribution of protein (CODEX) modality weights across major cell types in the WNN analysis. Strongest weights are observed for stromal cells.

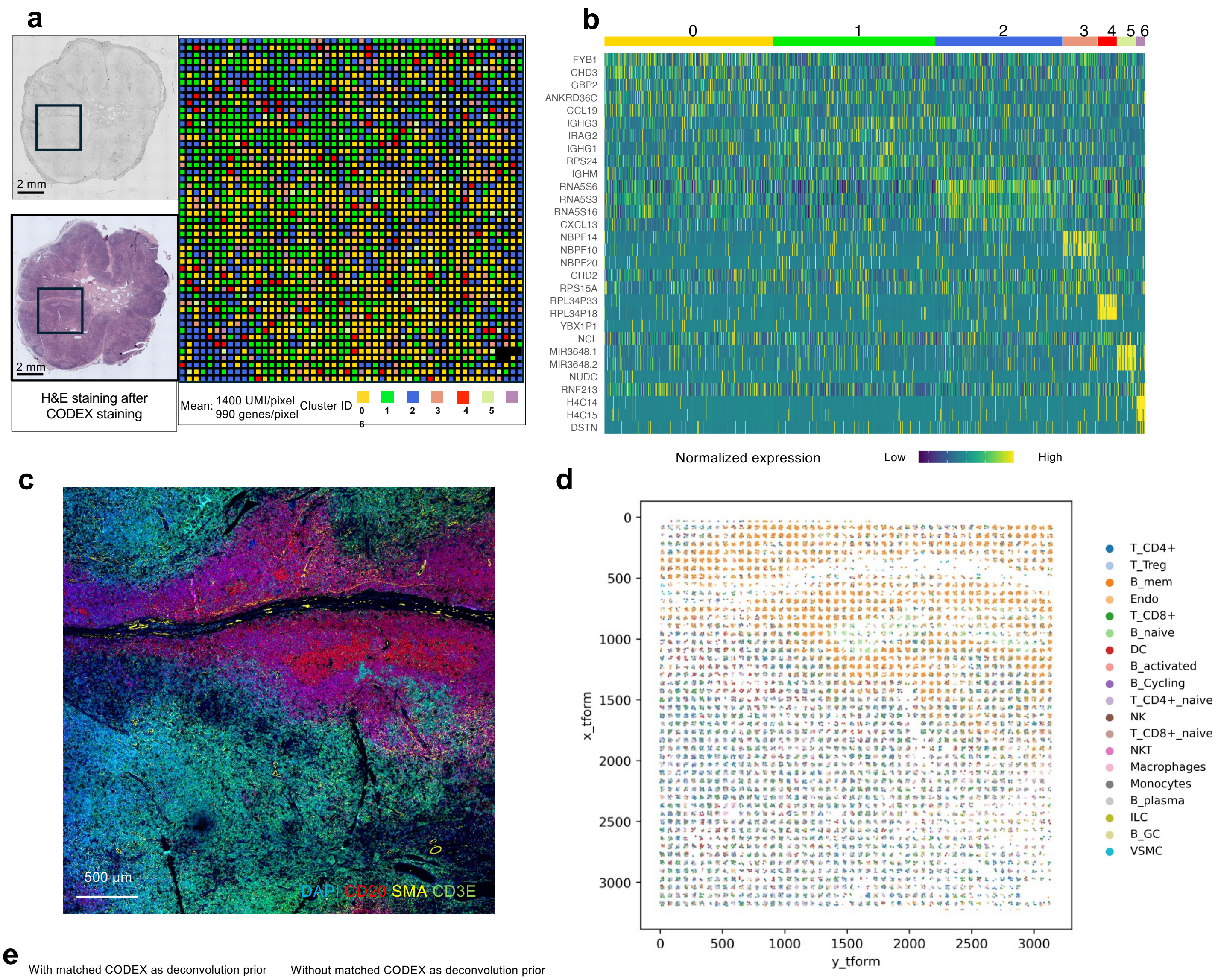

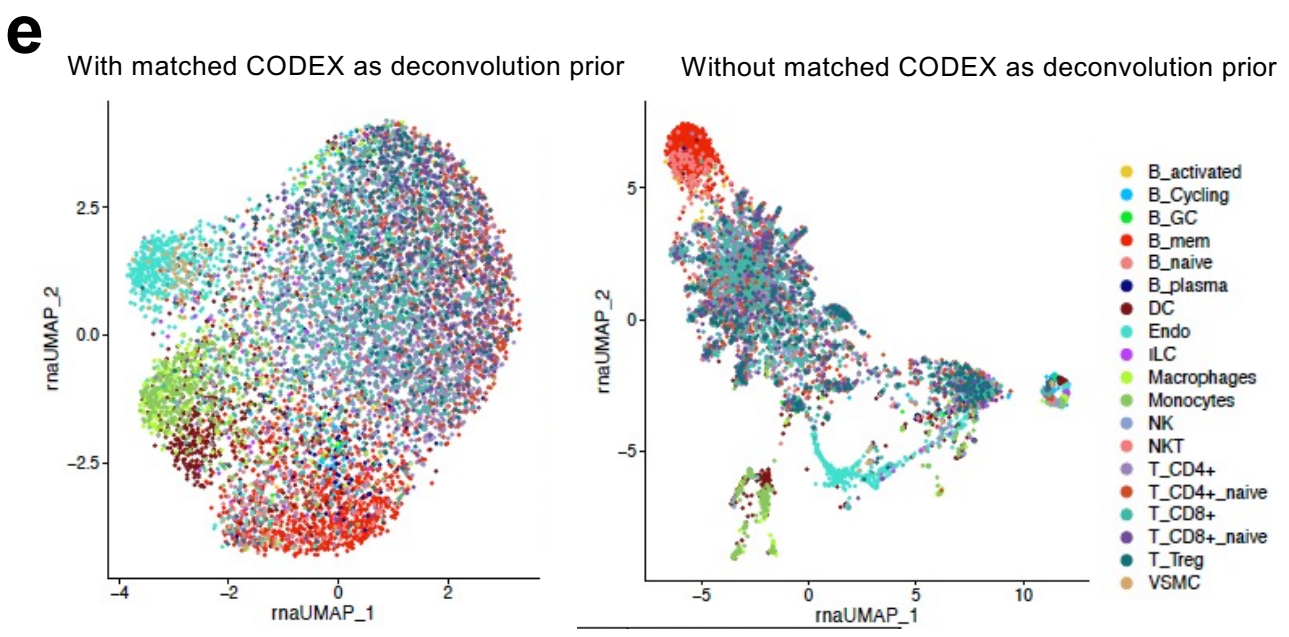

f

	With CODEX	Without CODEX
ASW	0.45	0.43
ARI	0.15	0.07

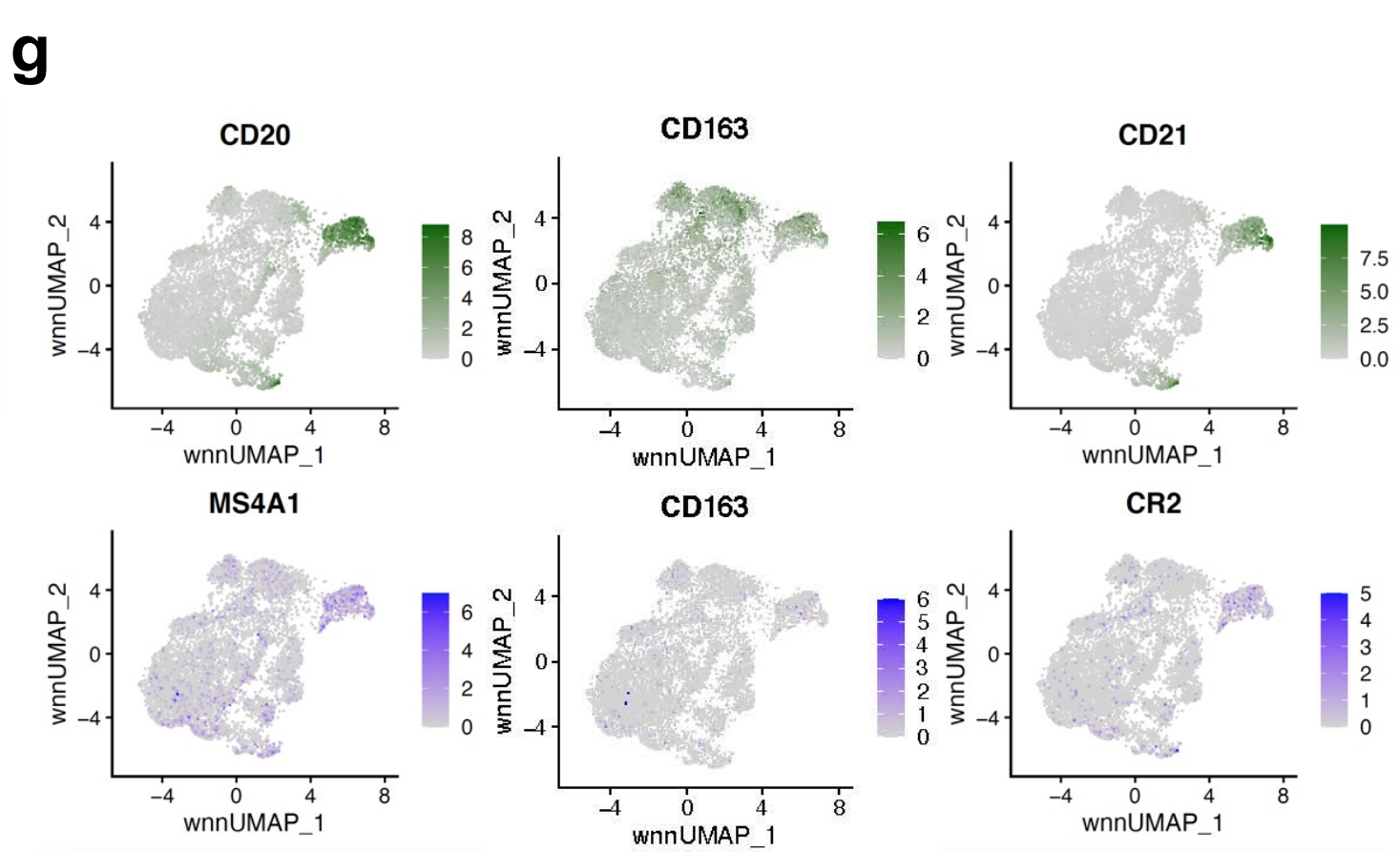

Supplementary Fig. 5. Spatial multi-omics profiling of FFPE human lymph node tissue section (25 μm resolution). a Spatial transcriptome distribution of FFPE human lymph node tissues barcoded using the microfluidic device with 25 μm resolution (n = 1). Black box represents the DBiTplus ROI. Unsupervised clustering identified 7 distinct clusters whose spatial distribution closely matches the histological organization of human lymph nodes. **b** Heatmap showing the top 7 differentially expressed genes for each spatial cluster. **c** CODEX staining (panel of 35 markers) is performed on the same tissue section following the DBiTplus workflow (n = 1). Scale bar 1mm. **d** Deconvolution of DBiTplus spots mapped by CODEX dataset. The identities of each cell within the DBiTplus spots can be directly inferred. **e** UMAPs of deconvoluted DBiTplus data: CODEX-informed deconvolution of DBiTplus feature (Left) and DBiTplus deconvolution without CODEX information (Right). **f** Higher ASW and ARI scores achieved by CODEX-informed deconvolution of DBiTplus spots suggesting better spot deconvolution quality. **g** Comparison of expression levels of protein features from CODEX (top) and corresponding RNA from DBiTplus after deconvolution (bottom).

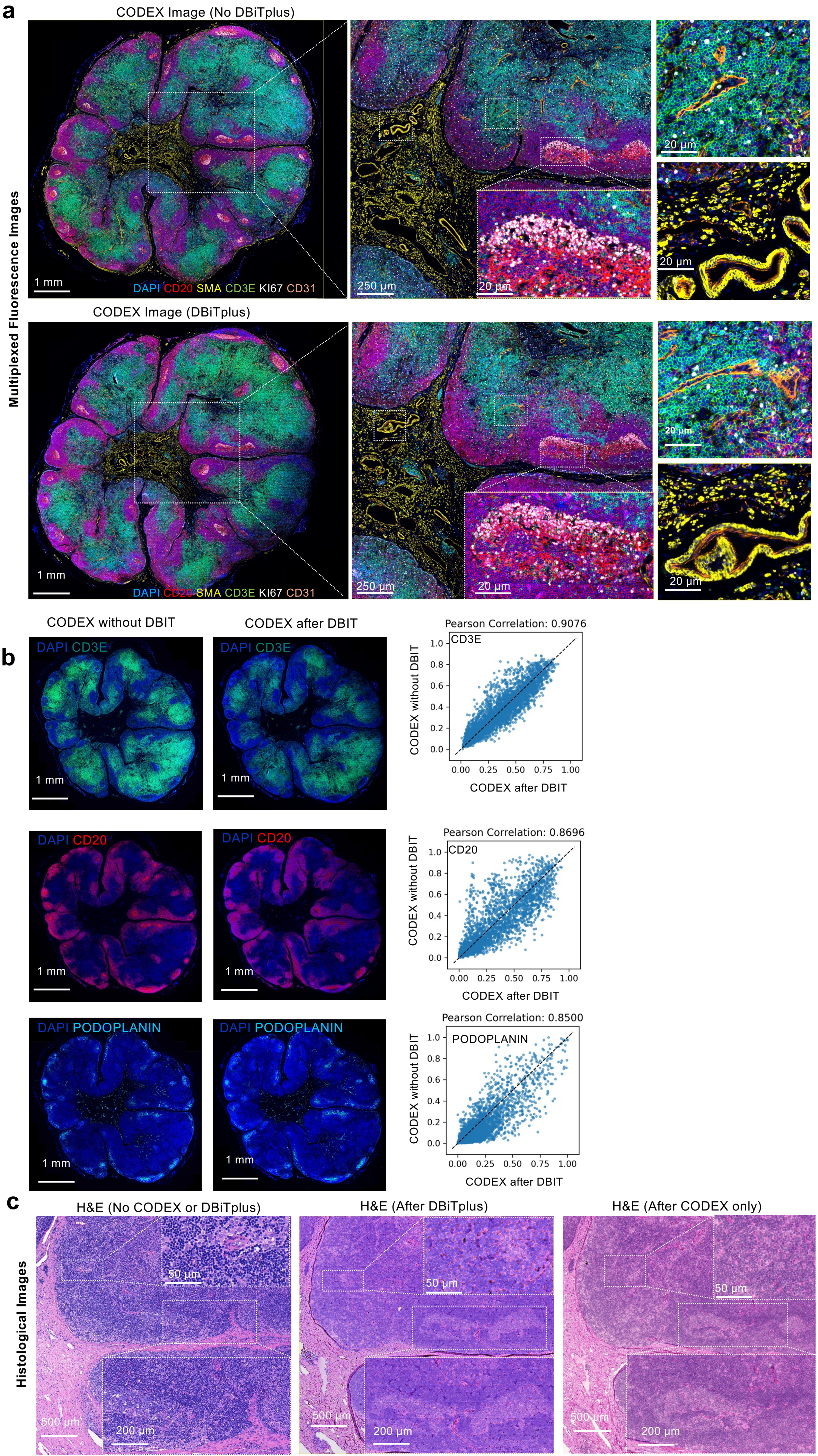
a
CODEX Image (No DBiTplus)
CODEX Image (DBiTplus)
Multiplexed Fluorescence Images
1 mm
250 µm
20 µm
DAPI CD20 SMA CD3E KI67 CD31
b
CODEX without DBIT
CODEX after DBIT
DAPI CD3E
DAPI CD20
DAPI PODOPLANIN
Pearson Correlation: 0.9076
Pearson Correlation: 0.8696
Pearson Correlation: 0.8500
CD3E
CD20
PODOPLANIN
CODEX without DBIT
CODEX after DBIT
c
H&E (No CODEX or DBiTplus)
H&E (After DBiTplus)
H&E (After CODEX only)
Histological Images
500 µm
200 µm
50 µm

Supplementary Fig. 6. Assessing multiplexed immunofluorescence imaging protein integrity after DBiTplus workflow. a Comparison of multiplexed fluorescence CODEX images from adjacent FFPE lymph node tissue without DBiTplus (top) (n = 1) and with DBiTplus (bottom) (n = 1). High-resolution zoom-in panels show comparable staining intensity and spatial patterns across cellular and vascular compartments. **b** Correlation analysis between two adjacent sections stained via CODEX. Left: Tissue section stained via CODEX (n = 1). Middle panel: Tissue section stained via CODEX after the DBiTplus workflow (n = 1). Right: Scatter plot showing strong correlation in expression levels between the two samples (Pearson R = 0.91 for CD3E, R = 0.87 for CD20, and R = 0.85 for PODOPLANIN), confirming that high-quality CODEX data can be obtained from a tissue section following the DBiTplus workflow. **c** Assessing Hematoxylin and Eosin (H&E) staining quality after DBiTplus. Regular H&E tissue section with no processing (n = 1), left), after DBiTplus (n = 1, middle), and H&E stain after CODEX alone (n = 1, right), showing minimal morphological distortion or loss of histological integrity and comparable quality between middle and right panels.

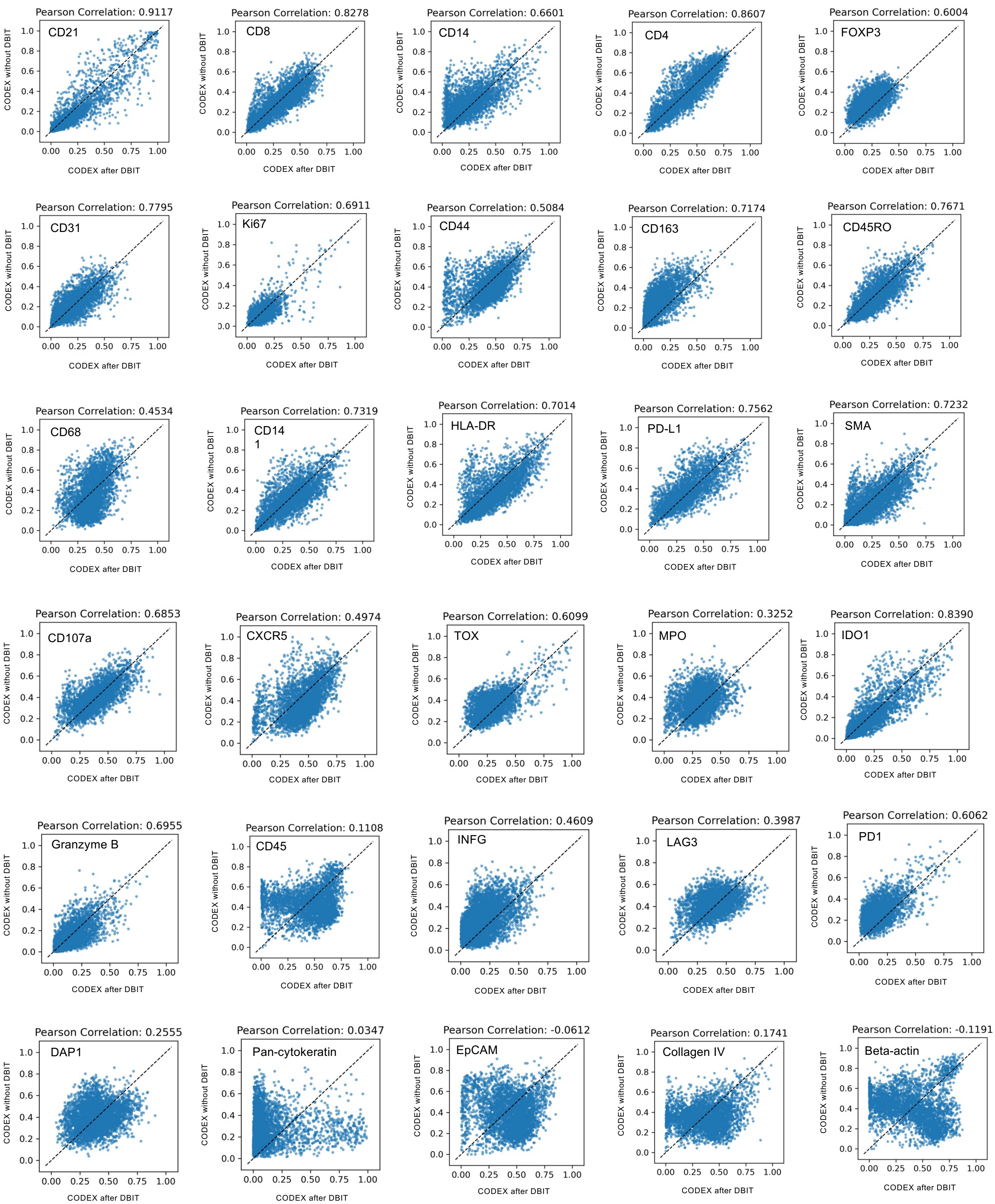

Supplementary Fig. 7. Marker-wise comparison of CODEX protein expression with and without DBiTplus. Scatter plots show pairwise comparison of mean-normalized CODEX expression for each marker with and without DBiTplus from two adjacent FFPE lymph node section. Pearson correlation coefficients are annotated above each panel. Each dot represents a protein, and black dotted line indicates linear regression fits. Strong correlation is observed for most markers. Lowest correlations observed for markers such as Beta-actin and EpCAM.

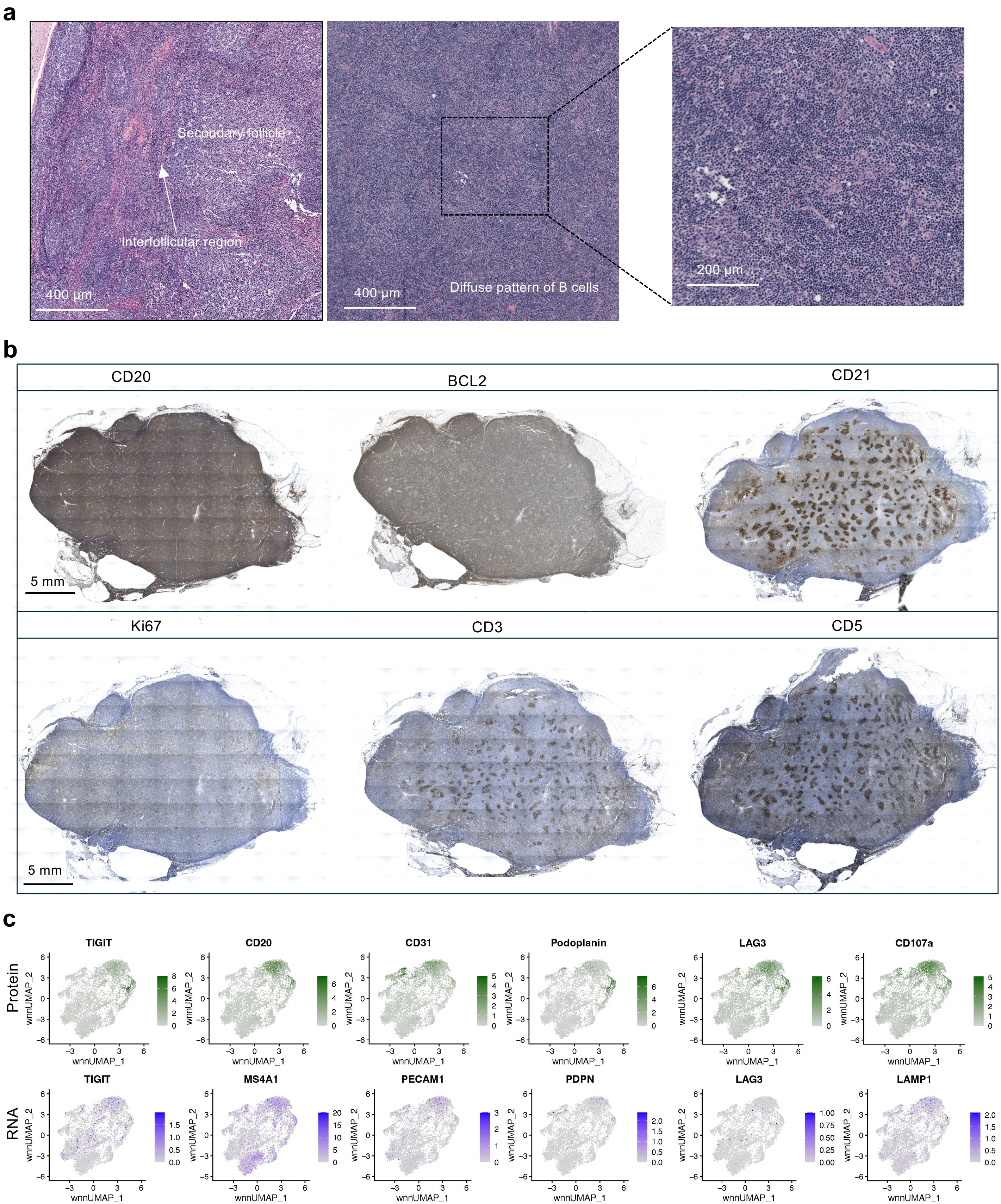

Supplementary Fig. 8. Histopathological and molecular profiling of MZL with increased large B cells transformation. a H&E-stained section showing normal lymph node architecture (n = 1) with secondary follicles and interfollicular zones (left), alongside a region with a diffuse sheet-like infiltration of B cells (n = 1) suggestive of high-grade transformation (middle and right). **b** Clinical Immunohistochemistry (IHC) staining of CD20 and BCL2 positivity, consistent with a B cell phenotype. CD21 highlights follicular dendritic cell meshwork, while Ki67 shows increased proliferation. CD3 and CD5 identify scattered T cells. These were for clinical diagnosis and were done once (n = 1) **c** Concordance of protein (top, CODEX) and mRNA (bottom, DBiTplus) expression for key lymphoma-related markers.

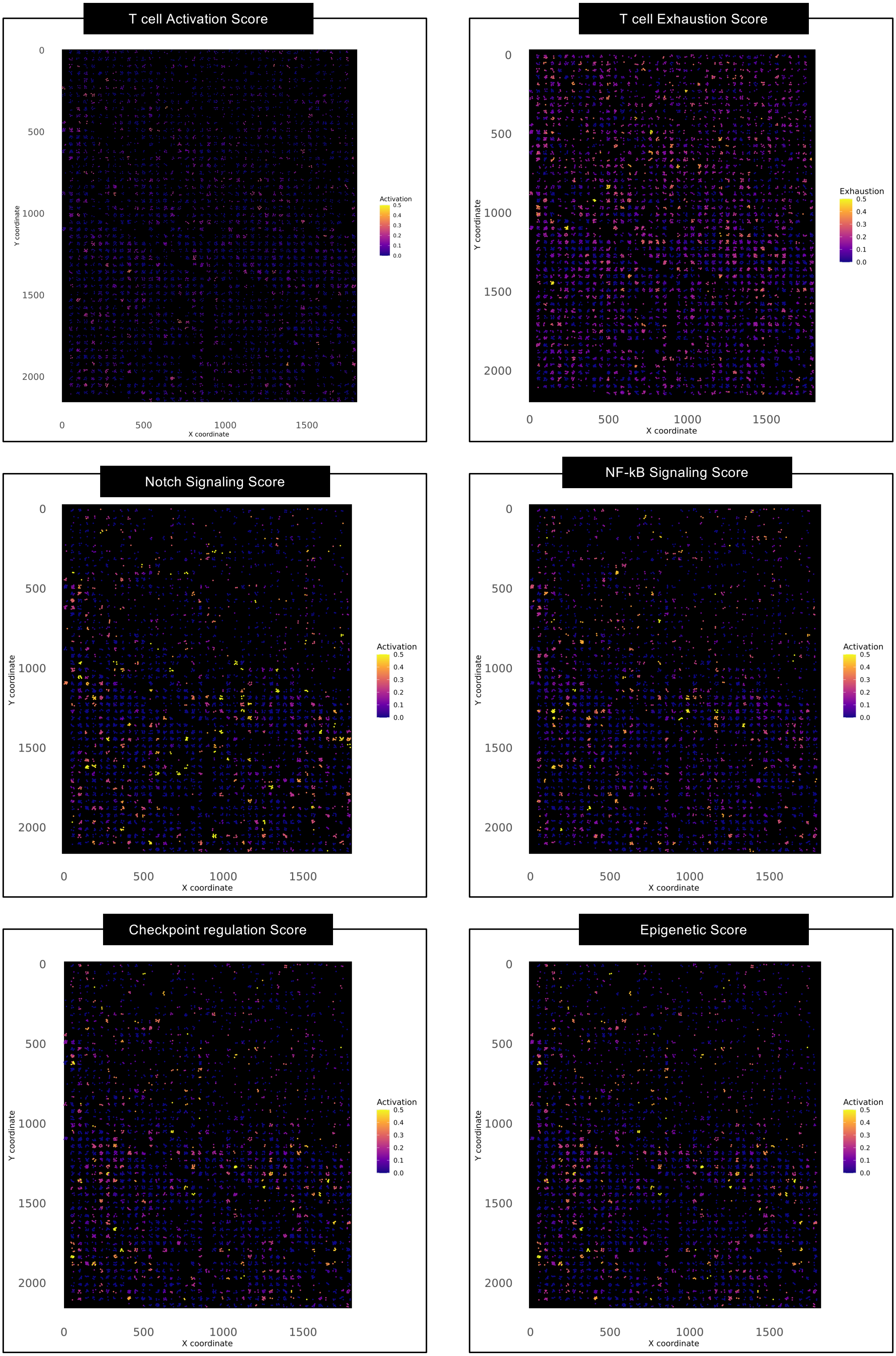

T cell Activation Score
Activation
0.5
0.4
0.3
0.2
0.1
0.0
Y coordinate
X coordinate
0
500
1000
1500
2000
T cell Exhaustion Score
Exhaustion
Notch Signaling Score
NF-kB Signaling Score
Checkpoint regulation Score
Epigenetic Score

Supplementary Fig. 9. Spatial pathway activity scores in Richter's Transformation. Spatial visualization of pathway module scores across the tissue section reveals microregional heterogeneity in immune and oncogenic signaling. **Top row:** T cell activation and exhaustion scores highlight spatially distinct zones of immune engagement and dysfunction. **Middle row:** Notch and NF-κB signaling scores, key regulators of lymphomagenesis, show punctate enrichment in distinct regions. **Bottom row:** Checkpoint regulation and epigenetic reprogramming scores reveal localized immunosuppressive and chromatin remodeling signatures.

Supplementary Table 1: Summary of gene expression metrics for all samples sequenced.

Sample	Channel size (μm)	Raw reads	Number of unique genes	Genes per pixel	UMIs per pixel
FF Mouse Embryo Control V1	25	204,654,134	23,295	1,245	1,918
FF Mouse Embryo RNaseH V1	25	230,056,530	20,089	518	678
FF Mouse Embryo Control V2	25	199,217,052	26,359	2,312	3,969
FF Mouse Embryo RNaseH V2	25	403,888,604	22,502	1,029	1,601
C57 Embryo Sagittal Paraffin Sections, E11	50	237,477,206	29,977	1,431	3,332
C57 Embryo Sagittal Paraffin Sections, E11 (Control)	50	175,095,812	36,739	1,004	2,398
Lymph node 2 Paraffin section (50 μm)	50	450,059,046	38,366	1,617	2,760
Lymph node 2 Paraffin section (25 μm)	25	597,416,230	31,027	801	1,434
MZL to DLBCL Lymphoma, Paraffin section	25	654,419,052	36,515	1,777	3,439
CLL to DLBCL Lymphoma, Paraffin section **	25	639,720,754	22,406	1,687	3,827

** This sample was preprocessed following the ASTRO Pipeline (Zhang, D. *et al. ASTRO: Automated Spatial Whole-Transcriptome RNA-Expression Workflow* (Cold Spring Harbor Laboratory, 2025), also utilized in Bai, Z. et al. Cell (2024).

Supplementary Table 2: Comparison of genes and UMI counts from the spatial profiling of FF mouse embryos

Sample	Number of Raw reads	Number of unique genes	Number of Genes per pixel	Number of UMIs per pixel
FF Mouse Embryo Control V1	99000000	24102	1374	2179
FF Mouse Embryo RNaseH V1	99000000	20973	591	782
FF Mouse Embryo Control V2	99000000	27370	2557	4575
FF Mouse Embryo RNaseH V2	99000000	22660	906	1364

Summary of raw reads, total number of unique genes, and genes and UMIs per 25 μm spot for FF mouse embryo experiments. The number of reads was downsampled to 99 million reads for all the samples to allow for this comparison.

Supplementary Table 3: Summary of marker staining quality from multiplexed immunofluorescence imaging experiments

Tissue Type	Species	Number of markers in panel	Number of successful markers	% of successful markers
Spleen, OCT	Mouse	25	14	56%
C57 Embryo Sagittal Paraffin Sections, E11	Mouse	21	18	86%
Cerebellum, Paraffin section	Human	28	24	86%
MZL to DLBCL Lymphoma, Paraffin section	Human	44	40	90%
Lymph node 1, Paraffin section	Human	46	44	92%
Lymph node 2 Paraffin section (50 μm)	Human	35	33	94%
Lymph node 2 Paraffin section (25 μm)	Human	35	33	94%
Lymph node 2 Paraffin section (Control)	Human	49	47	96%
CLL to DLBCL Lymphoma, Paraffin section	Human	30	22	73%

Supplementary Table 4: Summary of age and RNA QC for FFPE tissue blocks

FFPE block	Source	Species	Age (in years)	RIN	DV_{200}
Lymph node 1 Paraffin section	Clinical	Human	3	1.0	69.4%
Lymph node 2 Paraffin section	Clinical	Human	1	1.9	48.2%
MZL to DLBCL Lymphoma, Paraffin section	Clinical	Human	5	1.6	48.3%
CLL to DLBCL Lymphoma, Paraffin section	Clinical	Human	2	NA	29.3%
C57 Embryo Sagittal Paraffin Sections, E11	Commercial	Mouse	< 1	NA	NA
Cerebellum, Paraffin section	Commercial	Human	< 1	NA	NA

Supplementary Table 5: DNA oligos for PCR, ligation and library preparation

Oligo	Sequence
RT primer	/5Phos/CATCGGCGTACGACTNNNNNNNNNN/iBiodT/TTTTTTTTTTTTTTTVN
Ligation linker 1	AGTCGTACGCCGATGCGAAACATCGGCCAC
Ligation linker 2	CGAATGCTCTGGCCTCTCAAGCACGTGGAT
PCR Primer 1	CAAGCGTTGGCTTCTCGCATCT
PCR Primer 2	AAGCAGTGGTATCAACGCAGAGT
Template Switch Oligo	AAGCAGTGGTATCAACGCAGAGTGAATrGrG+G
dN-SMRT	AAGCAGTGGTATCAACGCAGAGTGANNNGGNNNB
P5 oligo for FFPE direct ligation	AATGATACGGCGACCACCGAGATCTACACTAGATCGCTCGTCGGCAGCGTCAG ATGTGTATAAGAGACAGAAGCAGTGGTATCAACGCAGAGT
N501	AATGATACGGCGACCACCGAGATCTACACTAGATCGCTCGTCGGCAGCGTCAGA TGTGTATAAGAGACAG
N701	CAAGCAGAAGACGGCATACGAGATTCGCCTTAGTCTCGTGGGCTCGGAGATGTG TATAAGAGACAGCAAGCGTTGGCTTCTCGCATCT
N702	CAAGCAGAAGACGGCATACGAGATCTAGTACGGTCTCGTGGGCTCGGAGATGTG TATAAGAGACAGCAAGCGTTGGCTTCTCGCATCT
N703	CAAGCAGAAGACGGCATACGAGATTTCTGCCTGTCTCGTGGGCTCGGAGATGTG TATAAGAGACAGCAAGCGTTGGCTTCTCGCATCT
N704	CAAGCAGAAGACGGCATACGAGATGCTCAGGAGTCTCGTGGGCTCGGAGATGT GTATAAGAGACAGCAAGCGTTGGCTTCTCGCATCT
N705	CAAGCAGAAGACGGCATACGAGATAGGAGTCCGTCTCGTGGGCTCGGAGATGT GTATAAGAGACAGCAAGCGTTGGCTTCTCGCATCT
N706	CAAGCAGAAGACGGCATACGAGATCATGCCTAGTCTCGTGGGCTCGGAGATGTG TATAAGAGACAGCAAGCGTTGGCTTCTCGCATCT
N707	CAAGCAGAAGACGGCATACGAGATGTAGAGAGGTCTCGTGGGCTCGGAGATGT GTATAAGAGACAGCAAGCGTTGGCTTCTCGCATCT

Supplementary Table 6: DNA barcode A sequences

Barcode A	Sequence
Barcode A-1	/5Phos/AGGCCAGAGCATTCGAACGTGATGTGGCCGATGTTTCG
Barcode A-2	/5Phos/AGGCCAGAGCATTCGAAACATCGGTGGCCGATGTTTCG
Barcode A-3	/5Phos/AGGCCAGAGCATTCGATGCCTAAGTGGCCGATGTTTCG
Barcode A-4	/5Phos/AGGCCAGAGCATTCGAGTGGTCAGTGGCCGATGTTTCG
Barcode A-5	/5Phos/AGGCCAGAGCATTCGACCACTGTGTGGCCGATGTTTCG
Barcode A-6	/5Phos/AGGCCAGAGCATTCGACATTGGCGTGGCCGATGTTTCG
Barcode A-7	/5Phos/AGGCCAGAGCATTCGCAGATCTGGTGGCCGATGTTTCG
Barcode A-8	/5Phos/AGGCCAGAGCATTCGCATCAAGTGTGGCCGATGTTTCG
Barcode A-9	/5Phos/AGGCCAGAGCATTCGCGCTGATCGTGGCCGATGTTTCG
Barcode A-10	/5Phos/AGGCCAGAGCATTCGACAAGCTAGTGGCCGATGTTTCG
Barcode A-11	/5Phos/AGGCCAGAGCATTCGCTGTAGCCGTGGCCGATGTTTCG
Barcode A-12	/5Phos/AGGCCAGAGCATTCGAGTACAAGGTGGCCGATGTTTCG
Barcode A-13	/5Phos/AGGCCAGAGCATTCGAACAACCAGTGGCCGATGTTTCG
Barcode A-14	/5Phos/AGGCCAGAGCATTCGAACCGAGAGTGGCCGATGTTTCG
Barcode A-15	/5Phos/AGGCCAGAGCATTCGAACGCTTAGTGGCCGATGTTTCG
Barcode A-16	/5Phos/AGGCCAGAGCATTCGAAGACGGAGTGGCCGATGTTTCG
Barcode A-17	/5Phos/AGGCCAGAGCATTCGAAGGTACAGTGGCCGATGTTTCG
Barcode A-18	/5Phos/AGGCCAGAGCATTCGACACAGAAGTGGCCGATGTTTCG
Barcode A-19	/5Phos/AGGCCAGAGCATTCGACAGCAGAGTGGCCGATGTTTCG
Barcode A-20	/5Phos/AGGCCAGAGCATTCGACCTCCAAGTGGCCGATGTTTCG
Barcode A-21	/5Phos/AGGCCAGAGCATTCGACGCTCGAGTGGCCGATGTTTCG
Barcode A-22	/5Phos/AGGCCAGAGCATTCGACGTATCAGTGGCCGATGTTTCG
Barcode A-23	/5Phos/AGGCCAGAGCATTCGACTATGCAGTGGCCGATGTTTCG
Barcode A-24	/5Phos/AGGCCAGAGCATTCGAGAGTCAAGTGGCCGATGTTTCG
Barcode A-25	/5Phos/AGGCCAGAGCATTCGAGATCGCAGTGGCCGATGTTTCG
Barcode A-26	/5Phos/AGGCCAGAGCATTCGAGCAGGAAGTGGCCGATGTTTCG
Barcode A-27	/5Phos/AGGCCAGAGCATTCGAGTCACTAGTGGCCGATGTTTCG
Barcode A-28	/5Phos/AGGCCAGAGCATTCGATCCTGTAGTGGCCGATGTTTCG
Barcode A-29	/5Phos/AGGCCAGAGCATTCGATTGAGGAGTGGCCGATGTTTCG
Barcode A-30	/5Phos/AGGCCAGAGCATTCGCAACCACAGTGGCCGATGTTTCG
Barcode A-31	/5Phos/AGGCCAGAGCATTCGGACTAGTAGTGGCCGATGTTTCG
Barcode A-32	/5Phos/AGGCCAGAGCATTCGCAATGGAAGTGGCCGATGTTTCG
Barcode A-33	/5Phos/AGGCCAGAGCATTCGCACTTCGAGTGGCCGATGTTTCG
Barcode A-34	/5Phos/AGGCCAGAGCATTCGCAGCGTTAGTGGCCGATGTTTCG
Barcode A-35	/5Phos/AGGCCAGAGCATTCGCATACCAAGTGGCCGATGTTTCG
Barcode A-36	/5Phos/AGGCCAGAGCATTCGCCAGTTCAGTGGCCGATGTTTCG
Barcode A-37	/5Phos/AGGCCAGAGCATTCGCCGAAGTAGTGGCCGATGTTTCG
Barcode A-38	/5Phos/AGGCCAGAGCATTCGCCGTGAGAGTGGCCGATGTTTCG
Barcode A-39	/5Phos/AGGCCAGAGCATTCGCCTCCTGAGTGGCCGATGTTTCG
Barcode A-40	/5Phos/AGGCCAGAGCATTCGCGAACTTAGTGGCCGATGTTTCG
Barcode A-41	/5Phos/AGGCCAGAGCATTCGCGACTGGAGTGGCCGATGTTTCG

Barcode A-42	/5Phos/AGGCCAGAGCATTCGCGCATACAGTGGCCGATGTTTCG
Barcode A-43	/5Phos/AGGCCAGAGCATTCGCTCAATGAGTGGCCGATGTTTCG
Barcode A-44	/5Phos/AGGCCAGAGCATTCGCTGAGCCAGTGGCCGATGTTTCG
Barcode A-45	/5Phos/AGGCCAGAGCATTCGCTGGCATAGTGGCCGATGTTTCG
Barcode A-46	/5Phos/AGGCCAGAGCATTCGGAATCTGAGTGGCCGATGTTTCG
Barcode A-47	/5Phos/AGGCCAGAGCATTCGCAAGACTAGTGGCCGATGTTTCG
Barcode A-48	/5Phos/AGGCCAGAGCATTCGGAGCTGAAGTGGCCGATGTTTCG
Barcode A-49	/5Phos/AGGCCAGAGCATTCGGATAGACAGTGGCCGATGTTTCG
Barcode A-50	/5Phos/AGGCCAGAGCATTCGGCCACATAGTGGCCGATGTTTCG

Supplementary Table 7: DNA barcode B sequences

Barcode B	Sequence
Barcode B-1	CAAGCGTTGGCTTCTCGCATCTAACGTGATATCCACGTGCTTGAG
Barcode B-2	CAAGCGTTGGCTTCTCGCATCTAAACATCGATCCACGTGCTTGAG
Barcode B-3	CAAGCGTTGGCTTCTCGCATCTATGCCTAAATCCACGTGCTTGAG
Barcode B-4	CAAGCGTTGGCTTCTCGCATCTAGTGGTCAATCCACGTGCTTGAG
Barcode B-5	CAAGCGTTGGCTTCTCGCATCTACCACTGTATCCACGTGCTTGAG
Barcode B-6	CAAGCGTTGGCTTCTCGCATCTACATTGGCATCCACGTGCTTGAG
Barcode B-7	CAAGCGTTGGCTTCTCGCATCTCAGATCTGATCCACGTGCTTGAG
Barcode B-8	CAAGCGTTGGCTTCTCGCATCTCATCAAGTATCCACGTGCTTGAG
Barcode B-9	CAAGCGTTGGCTTCTCGCATCTCGCTGATCATCCACGTGCTTGAG
Barcode B-10	CAAGCGTTGGCTTCTCGCATCTACAAGCTAATCCACGTGCTTGAG
Barcode B-11	CAAGCGTTGGCTTCTCGCATCTCTGTAGCCATCCACGTGCTTGAG
Barcode B-12	CAAGCGTTGGCTTCTCGCATCTAGTACAAGATCCACGTGCTTGAG
Barcode B-13	CAAGCGTTGGCTTCTCGCATCTAACAACCAATCCACGTGCTTGAG
Barcode B-14	CAAGCGTTGGCTTCTCGCATCTAACCGAGAATCCACGTGCTTGAG
Barcode B-15	CAAGCGTTGGCTTCTCGCATCTAACGCTTAATCCACGTGCTTGAG
Barcode B-16	CAAGCGTTGGCTTCTCGCATCTAAGACGGAATCCACGTGCTTGAG
Barcode B-17	CAAGCGTTGGCTTCTCGCATCTAAGGTACAATCCACGTGCTTGAG
Barcode B-18	CAAGCGTTGGCTTCTCGCATCTACACAGAAATCCACGTGCTTGAG
Barcode B-19	CAAGCGTTGGCTTCTCGCATCTACAGCAGAATCCACGTGCTTGAG
Barcode B-20	CAAGCGTTGGCTTCTCGCATCTACCTCCAAATCCACGTGCTTGAG
Barcode B-21	CAAGCGTTGGCTTCTCGCATCTACGCTCGAATCCACGTGCTTGAG
Barcode B-22	CAAGCGTTGGCTTCTCGCATCTACGTATCAATCCACGTGCTTGAG
Barcode B-23	CAAGCGTTGGCTTCTCGCATCTACTATGCAATCCACGTGCTTGAG
Barcode B-24	CAAGCGTTGGCTTCTCGCATCTAGAGTCAAATCCACGTGCTTGAG
Barcode B-25	CAAGCGTTGGCTTCTCGCATCTAGATCGCAATCCACGTGCTTGAG
Barcode B-26	CAAGCGTTGGCTTCTCGCATCTAGCAGGAAATCCACGTGCTTGAG
Barcode B-27	CAAGCGTTGGCTTCTCGCATCTAGTCACTAATCCACGTGCTTGAG
Barcode B-28	CAAGCGTTGGCTTCTCGCATCTATCCTGTAATCCACGTGCTTGAG
Barcode B-29	CAAGCGTTGGCTTCTCGCATCTATTGAGGAATCCACGTGCTTGAG
Barcode B-30	CAAGCGTTGGCTTCTCGCATCTCAACCACAATCCACGTGCTTGAG
Barcode B-31	CAAGCGTTGGCTTCTCGCATCTGACTAGTAATCCACGTGCTTGAG
Barcode B-32	CAAGCGTTGGCTTCTCGCATCTCAATGGAAATCCACGTGCTTGAG
Barcode B-33	CAAGCGTTGGCTTCTCGCATCTCACTTCGAATCCACGTGCTTGAG
Barcode B-34	CAAGCGTTGGCTTCTCGCATCTCAGCGTTAATCCACGTGCTTGAG
Barcode B-35	CAAGCGTTGGCTTCTCGCATCTCATACCAAATCCACGTGCTTGAG
Barcode B-36	CAAGCGTTGGCTTCTCGCATCTCCAGTTCAATCCACGTGCTTGAG
Barcode B-37	CAAGCGTTGGCTTCTCGCATCTCCGAAGTAATCCACGTGCTTGAG
Barcode B-38	CAAGCGTTGGCTTCTCGCATCTCCGTGAGAATCCACGTGCTTGAG
Barcode B-39	CAAGCGTTGGCTTCTCGCATCTCCTCCTGAATCCACGTGCTTGAG
Barcode B-40	CAAGCGTTGGCTTCTCGCATCTCGAACTTAATCCACGTGCTTGAG
Barcode B-41	CAAGCGTTGGCTTCTCGCATCTCGACTGGAATCCACGTGCTTGAG

Barcode B-42	CAAGCGTTGGCTTCTCGCATCTCGCATACAATCCACGTGCTTGAG
Barcode B-43	CAAGCGTTGGCTTCTCGCATCTCTCAATGAATCCACGTGCTTGAG
Barcode B-44	CAAGCGTTGGCTTCTCGCATCTCTGAGCCAATCCACGTGCTTGAG
Barcode B-45	CAAGCGTTGGCTTCTCGCATCTCTGGCATAATCCACGTGCTTGAG
Barcode B-46	CAAGCGTTGGCTTCTCGCATCTGAATCTGAATCCACGTGCTTGAG
Barcode B-47	CAAGCGTTGGCTTCTCGCATCTCAAGACTAATCCACGTGCTTGAG
Barcode B-48	CAAGCGTTGGCTTCTCGCATCTGAGCTGAAATCCACGTGCTTGAG
Barcode B-49	CAAGCGTTGGCTTCTCGCATCTGATAGACAATCCACGTGCTTGAG
Barcode B-50	CAAGCGTTGGCTTCTCGCATCTGCCACATAATCCACGTGCTTGAG

Supplementary Table 8: Chemicals and reagents

Name	Catalog number	Vendor
Formaldehyde solution	PI28906	Thermo Fisher Scientific
Triton X-100	T8787	Sigma-Aldrich
T4 DNA Ligase	M0202L	New England Biolabs
T4 DNA Ligase Reaction Buffer	B0202S	New England Biolabs
NEBuffer 3.1	B7203S	New England Biolabs
DPBS	14190144	Thermo Fisher Scientific
Proteinase K	EO0491	Thermo Fisher Scientific
SYBR Green I Nucleic Acid Gel Stain	S7563	Thermo Fisher Scientific
Maxima H Minus Reverse Transcriptase (200 U/L)	EP0751	Thermo Fisher Scientific
dNTP mix	R0192	Thermo Fisher Scientific
SUPERase In RNase Inhibitor	AM2694	Thermo Fisher Scientific
RNase Inhibitor	Y9240L	Enzymatics
Kapa Hotstart HiFi ReadyMix	KK2601	Kapa Biosystems
Nextera XT DNA Preparation Kit	FC-131-1024	Illumina
Tween-20	11332465001	Sigma-Aldrich
EDTA Solution pH 8	AB00502	AmericanBio
DNA Clean & Concentrator-5	D4014	Zymo Research
Dynabeads™ MyOne™ Streptavidin C1	65001	Thermo Fisher
RNase free water	10977015	Invitrogen
Ethanol	187380-4L	Sigma
Ficoll Type 400 20% solution	F54145	Sigma-Aldrich
Klenow Fragment (3'→5' exo-)	M0212S	New England BioLabs
Thermostable RNase H	M0523S	New England BioLabs
Xylene	247642-18L-CS	Sigma-Aldrich
HistoChoice Clearing Agent	C913R27	Thomas Scientific
Methanol	LC168002	Thomas Scientific
Antigen Retrieval Buffer (100X Tris-EDTA Buffer, pH 9.0)	ab93684	Abcam
Poly(A) Polymerase, Yeast	74225Z25KU	Thermo Fisher Scientific
Discovery CC1 buffer	NC1719907	Roche Diagnostics
Thermo Scientific™ ATP Solution (100 mM)	FERR0441	Fisher Scientific
SEQuoia RiboDepletion Kit	17006487	Bio-Rad
