# Supplementary material for: Integration of imaging-based and sequencing-based spatial omics mapping on the same tissue section via DBiTplus
Source: Nat Methods. 2026 Jan 15;23(9):1827–39. doi: 10.1038/s41592-025-02948-0 (PMC13541625; doi:10.1038/s41592-025-02948-0)
Supplement: Supplementary file 2 [file 41592_2025_2948_MOESM2_ESM.pdf]

nature portfolio

Corresponding author(s): Rong Fan, Mina Xu, Zongming Ma

Last updated by author(s): Oct 4, 2025

Reporting Summary

Nature Portfolio wishes to improve the reproducibility of the work that we publish. This form provides structure for consistency and transparency in reporting. For further information on Nature Portfolio policies, see our Editorial Policies and the Editorial Policy Checklist.

Statistics

For all statistical analyses, confirm that the following items are present in the figure legend, table legend, main text, or Methods section.

n/a	Confirmed	
☐	☒	The exact sample size (*n*) for each experimental group/condition, given as a discrete number and unit of measurement
☐	☒	A statement on whether measurements were taken from distinct samples or whether the same sample was measured repeatedly
☐	☒	The statistical test(s) used AND whether they are one- or two-sided<br>*Only common tests should be described solely by name; describe more complex techniques in the Methods section.*
☐	☒	A description of all covariates tested
☐	☒	A description of any assumptions or corrections, such as tests of normality and adjustment for multiple comparisons
☐	☒	A full description of the statistical parameters including central tendency (e.g. means) or other basic estimates (e.g. regression coefficient) AND variation (e.g. standard deviation) or associated estimates of uncertainty (e.g. confidence intervals)
☐	☒	For null hypothesis testing, the test statistic (e.g. *F*, *t*, *r*) with confidence intervals, effect sizes, degrees of freedom and *P* value noted<br>*Give P values as exact values whenever suitable.*
☒	☐	For Bayesian analysis, information on the choice of priors and Markov chain Monte Carlo settings
☒	☐	For hierarchical and complex designs, identification of the appropriate level for tests and full reporting of outcomes
☐	☒	Estimates of effect sizes (e.g. Cohen's *d*, Pearson's *r*), indicating how they were calculated

Our web collection on statistics for biologists contains articles on many of the points above.

Software and code

Policy information about availability of computer code

Data collection	Akoya Biosciences PhenoCycler Fusion System, Agilent TapeStation 4150, Illumina NovaSeq X Plus System, Illumina NovaSeq 6000 System, Bruker Spatial Biology CellScape System, M7000 Imaging EVOS System, Motic EasyScan digital slide scanner
Data analysis	STAR V2.7.8a, ST_Pipeline V1.8.1, Seurat V4.3.0, Seqkit version 2.3.1, Ingenuity Pathway Analysis (IPA, QIAGEN, Version 01-23-01), Prism V10, R version 4.2.0, RStudio Server version: v2024.12.1, ASTRO Pipeline(Zhang, D. et al. ASTRO: Automated Spatial Whole-Transcriptome RNA-Expression Workflow (Cold Spring Harbor Laboratory, 2025). The code for the analyzing the processed multiplexed immunofluorescence imaging datasets and the sequencing datasets are archived at Zenodo (https://zenodo.org/records/16814952) and also available at GitHub (https://github.com/Janezjz/DBiT-plus).

For manuscripts utilizing custom algorithms or software that are central to the research but not yet described in published literature, software must be made available to editors and reviewers. We strongly encourage code deposition in a community repository (e.g. GitHub). See the Nature Portfolio guidelines for submitting code & software for further information.

Data

Policy information about availability of data

All manuscripts must include a data availability statement. This statement should provide the following information, where applicable:

- Accession codes, unique identifiers, or web links for publicly available datasets
- A description of any restrictions on data availability
- For clinical datasets or third party data, please ensure that the statement adheres to our policy

Raw and processed data reported are deposited in the Gene Expression Omnibus (GEO) with accession GSE308167. The resulting fastq files were aligned to the mouse reference genome (GRCm39) or human reference genome (GRCh38). Multiplexed immunofluorescence imaging datasets and Histological images are available at https://zenodo.org/records/17153112 and https://zenodo.org/records/17153187. Published data for data comparison are available online at STOmicsDB Mouse Organogenesis Spatiotemporal Transcriptomic Atlas (MOSTA) dataset (E13.5_E1S2.MOSTA.h5ad). (https://db.cngb.org/stomics/mosta/spatial/) and Allen Mouse Brain Atlas (Developing Mouse Brain - Age: E13.5, Theiler Stage: TS21) at link: https://developingmouse.brain-map.org/). Patho-DBiT reference lymph node data is available on GEO under accession number GSE274641; sample GSM8454083).

Research involving human participants, their data, or biological material

Policy information about studies with human participants or human data. See also policy information about sex, gender (identity/presentation), and sexual orientation and race, ethnicity and racism.

Reporting on sex and gender	Not relevant to this study
Reporting on race, ethnicity, or other socially relevant groupings	Not relevant to this study
Population characteristics	Not relevant to this study
Recruitment	Not relevant to this study
Ethics oversight	De-identified archived formalin-fixed paraffin-embedded (FFPE) human reactive lymph node and lymphoma tissue blocks were obtained from Yale Pathology Tissue Services (YPTS). The tissue retrieval and distribution for research was conducted with the approval of the Yale University Institutional Review Board (Approved IRB #1401013259) and oversight by the Tissue Resource Oversight Committee. Written informed consent for participation in any cases where identification was collected alongside the specimen, was obtained from patients or their guardians, in accordance with the principles of the Declaration of Helsinki. Each sample was handled in strict compliance with HIPAA regulations, University Research Policies, Pathology Department diagnostic requirements, and Hospital by-laws.

Note that full information on the approval of the study protocol must also be provided in the manuscript.

Field-specific reporting

Please select the one below that is the best fit for your research. If you are not sure, read the appropriate sections before making your selection.

☒ Life sciences ☐ Behavioural & social sciences ☐ Ecological, evolutionary & environmental sciences

For a reference copy of the document with all sections, see nature.com/documents/nr-reporting-summary-flat.pdf

Life sciences study design

All studies must disclose on these points even when the disclosure is negative.

Sample size	The goal of this study was to develop a new spatial omics technology. Sample size calculation was not performed as samples sizes were chosen primarily based on experiment length, sequencing costs and tissue types. The current manuscript mainly described a new method for the joint profiling of the transcriptome and proteome within the same slide and at single-cell resolution. The number of samples included in the study are sufficient to serve as a proof-of-concept for the new technology.
Data exclusions	No data were excluded.
Replication	Experiments on the mouse embryo samples were replicated. After demonstrating the reproducibility of the new technology, the subsequent experiments were performed once to serve as a proof-of-concept for the new technology.
Randomization	There are no defined sample groups so randomization was not particularly applicable in this study
Blinding	There are no defined sample groups so blinding was not particularly applicable in this study

Reporting for specific materials, systems and methods

We require information from authors about some types of materials, experimental systems and methods used in many studies. Here, indicate whether each material, system or method listed is relevant to your study. If you are not sure if a list item applies to your research, read the appropriate section before selecting a response.

Materials & experimental systems

n/a	Involved in the study
☐	☒ Antibodies
☒	☐ Eukaryotic cell lines
☒	☐ Palaeontology and archaeology
☐	☒ Animals and other organisms
☒	☐ Clinical data
☒	☐ Dual use research of concern
☒	☐ Plants

Methods

n/a	Involved in the study
☒	☐ ChIP-seq
☒	☐ Flow cytometry
☒	☐ MRI-based neuroimaging

Antibodies

Antibodies used

Cat No Target Clone Source
4250098 Bcl-2 EPR17509 Akoya Biosciences
4450040 Beta-actin W16197A Akoya Biosciences
4550119 CD3e EP449E Akoya Biosciences
4550112 CD4 EPR6855 Akoya Biosciences
4250012 CD8 C8/144B Akoya Biosciences
4550114 CD11c 118/A5 Akoya Biosciences
4450047 CD14 EPR3653 Akoya Biosciences
4450018 CD20 L26 Akoya Biosciences
4450027 CD21 EP3093 Akoya Biosciences
4450017 CD31 EP3095 Akoya Biosciences
4550133 CD34 QBEND10 Akoya Biosciences
4250057 CD34 QBEND10 Akoya Biosciences
4450041 CD44 156-3C11 Akoya Biosciences
4250099 CD45 D9M8I Akoya Biosciences
4250023 CD45RO UCHL1 Akoya Biosciences
4250087 CD56 CAL53 Akoya Biosciences
4550113 CD68 KP1 Akoya Biosciences
4450078 CD79a D1X5C Akoya Biosciences
4550098 CD107a H4A3 Akoya Biosciences
4250097 CD141 E7Y9P Akoya Biosciences
4250079 CD163 EPR19518 Akoya Biosciences
4550122 Collagen IV EPR20966 Akoya Biosciences
4250021 E-cadherin 4A2C7 Akoya Biosciences
4450088 EpCAM D9S3P Akoya Biosciences
4550071 FOXP3 236A/E7 Akoya Biosciences
4250055 Granzyme B D6E9W Akoya Biosciences
4450046 HLA-A EP1395Y Akoya Biosciences
4550118 HLA-DR EPR3692 Akoya Biosciences
4250065 HLA-E MEM- E/02 Akoya Biosciences
4550117 ICOS D1K2T Akoya Biosciences
4550123 IDO1 V1NC3IDO Akoya Biosciences
4250062 IFNG EPR21704 Akoya Biosciences
4250073 iNOS SP126 Akoya Biosciences
4250019 Ki67 B56 Akoya Biosciences
4550058 LAG3 EPR20261 Akoya Biosciences
4450034 Mac2/Galectin-3 M3/38 Akoya Biosciences
4250083 MPO E1E7I Akoya Biosciences
4450020 Pan-Cytokeratin AE-1/AE-3 Akoya Biosciences
4550124 PCNA PC10 Akoya Biosciences
4550038 PD-1 D4W2J Akoya Biosciences
4550072 PD-L1 73-10 Akoya Biosciences
4250004 Podoplanin NC-08 Akoya Biosciences
4250094 Podoplanin NC-08 Akoya Biosciences
4450049 SMA 1A4 Akoya Biosciences
4250075 SOX2 SP76 Akoya Biosciences
4250061 TIGIT BLR047F Akoya Biosciences
4250067 TOX E6I3Q Akoya Biosciences
4450050 Vimentin 091D3 Akoya Biosciences
4250063 VISTA D1L2G Akoya Biosciences

PA5-28827 CXCL13 - ThermoFisher Scientific
552032 CXCR5 RF8B2 BD Biosciences
PA5-14259 BCL6 - ThermoFisher Scientific

Mouse antibodies (Also from Akoya Biosciences)

Antibody Clone Reporter
CD90 30-H12 RX001-A488
CD31 MEC13.3 RX002-ATTO550
TCR H57-597 RX003-Cy5
Ter119 TER-119 RX004-A488
CD44 * IM7 RX005-ATTO550
Empty
CD45 30-F11 RX007-A488
CD19 6D5 RX020-ATTO550
CD169 3D6.112 RX015-Cy5
CD45R/B220 RA3-6B2 RX010-A488
MHCII M5/114.15 RX014-ATTO550
CD3 17A2 RX021-Cy5
IgM RMM-1 RX013-A488
CD5 53-7.3 RX017-ATTO550
Ly6g 1A8 RX024-Cy5
CD38 90 RX019-A488
CD21/35 7E9 RX023-ATTO550
CD71 RI7217 RX027-Cy5
IgD 11-26c.2a RX016-A488
CD4 RM4-5 RX026-ATTO550
CD11c N418 RX030-Cy5
CD24 M1/69 RX022-A488
CD8a 53-6.7 RX029-ATTO550
CD49f GoH3 RX033-Cy5
CD11b M1/70 RX025-A488
Ki67 * B56 CX047-ATTO550

CellScape system used the Vistaplex Antibody panel: CD19, CD20, CD23, CD5, LEF1, CD38, CD138, Ki-67
CD3, CD4, CD8, CD45RO, CD45RA, FoxP3, GranzymeB, CD279 (PD1), CD274 (PD-L1), CD45
CD31, CD34, CD105, Col IV, Podoplanin, CD68, CD163, B-Catenin
DNA, ATP1A1, LaminB1, B2M. The following were not available used in Bruker's Vistaplex Antibody panel and were purchased from a commercial vendor.
Antibody Clone Vendor
CD5 4C7 Leica
CD23 1B12 Leica
LEF1 EPR2029Y Abcam

Details of antibody dilutions and imaging cycles are included in supplementary file on imaging panel details

Validation

Commercial antibodies were validated for reactivity by the vendors. Antibodies conjugated in-house for use in CODEX were conjugated following Akoya Biosciences' antibody conjugation protocol (https://www.akoyabio.com/wp-content/uploads/2020/04/Antibody-Screening-and-Custom-Conjugation.pdf and https://www.akoyabio.com/wp-content/uploads/AbConjugation-Kit-for-PhenoCode-Signature-Protocol.pdf)

Animals and other research organisms

Policy information about studies involving animals; ARRIVE guidelines recommended for reporting animal research, and Sex and Gender in Research

Laboratory animals

All mice tissue used were C57BL/6 mice obtained from a commercial vendor, Zyagen (San Diego, CA).

Wild animals

No wild animals were included in this study

Reporting on sex

Not relevant to this study

Field-collected samples

No field-collected samples were included in this study

Ethics oversight

The mouse tissue used in this study was obtained from a commercial vendor, Zyagen (San Diego, CA), which procured and handled the animals under their in-house institutional IACUC-approved protocols.

Note that full information on the approval of the study protocol must also be provided in the manuscript.

Plants

Seed stocks	No seed stocks were used in this study
Novel plant genotypes	No plants were included in this study
Authentication	Not relevant to study
